# Implementation Facilitation to Promote Emergency Department–Initiated Buprenorphine for Opioid Use Disorder

**DOI:** 10.1001/jamanetworkopen.2023.5439

**Published:** 2023-04-05

**Authors:** Gail D’Onofrio, E. Jennifer Edelman, Kathryn F. Hawk, Marek C. Chawarski, Michael V. Pantalon, Patricia H. Owens, Shara H. Martel, Richard Rothman, Mustapha Saheed, Robert P. Schwartz, Ethan Cowan, Lynne Richardson, Edwin Salsitz, Michael S. Lyons, Caroline Freiermuth, Christine Wilder, Lauren Whiteside, Judith I. Tsui, Jared W. Klein, Edouard Coupet, Patrick G. O’Connor, Abigail G. Matthews, Sean M. Murphy, Kristen Huntley, David A. Fiellin

**Affiliations:** 1 Department of Emergency Medicine, Yale School of Medicine, New Haven, Connecticut; 2 Department of Internal Medicine, Yale School of Medicine, New Haven, Connecticut; 3 Yale School of Public Health, New Haven, Connecticut; 4 Department of Psychiatry, Yale School of Medicine, New Haven, Connecticut; 5 Department of Emergency Medicine, Johns Hopkins University School of Medicine, Baltimore, Maryland; 6 Friends Research Institute, Baltimore, Maryland; 7 Department of Emergency Medicine, Icahn School of Medicine at Mount Sinai, New York, New York; 8 Institute for Health Equity Research, Department of Emergency Medicine, Icahn School of Medicine at Mount Sinai, New York, New York; 9 Department of Psychiatry, Icahn School of Medicine at Mount Sinai, New York, New York; 10 Department of Emergency Medicine, University of Cincinnati, Cincinnati, Ohio; 11 Center for Addiction Research, University of Cincinnati College of Medicine, Cincinnati, Ohio; 12 Department of Psychiatry and Behavioral Neuroscience, University of Cincinnati, Cincinnati, Ohio; 13 Department of Emergency Medicine, University of Washington School of Medicine, Seattle,; 14 Department of Medicine, University of Washington, Seattle; 15 The Emmes Company, Rockville, Maryland; 16 Weill Cornell Medical College, New York, New York; 17 National Institute on Drug Abuse, Rockville, Maryland

## Abstract

**Importance:**

Emergency department (ED)–initiated buprenorphine for the treatment of opioid use disorder (OUD) is underused.

**Objective:**

To evaluate whether provision of ED-initiated buprenorphine with referral for OUD increased after implementation facilitation (IF), an educational and implementation strategy.

**Design, Setting, and Participants:**

This multisite hybrid type 3 effectiveness-implementation nonrandomized trial compared grand rounds with IF, with pre-post 12-month baseline and IF evaluation periods, at 4 academic EDs. The study was conducted from April 1, 2017, to November 30, 2020. Participants were ED and community clinicians treating patients with OUD and observational cohorts of ED patients with untreated OUD. Data were analyzed from July 16, 2021, to July 14, 2022.

**Exposure:**

A 60-minute in-person grand rounds was compared with IF, a multicomponent facilitation strategy that engaged local champions, developed protocols, and provided learning collaboratives and performance feedback.

**Main Outcomes and Measures:**

The primary outcomes were the rate of patients in the observational cohorts who received ED-initiated buprenorphine with referral for OUD treatment (primary implementation outcome) and the rate of patients engaged in OUD treatment at 30 days after enrollment (effectiveness outcome). Additional implementation outcomes included the numbers of ED clinicians with an X-waiver to prescribe buprenorphine and ED visits with buprenorphine administered or prescribed and naloxone dispensed or prescribed.

**Results:**

A total of 394 patients were enrolled during the baseline evaluation period and 362 patients were enrolled during the IF evaluation period across all sites, for a total of 756 patients (540 [71.4%] male; mean [SD] age, 39.3 [11.7] years), with 223 Black patients (29.5%) and 394 White patients (52.1%). The cohort included 420 patients (55.6%) who were unemployed, and 431 patients (57.0%) reported unstable housing. Two patients (0.5%) received ED-initiated buprenorphine during the baseline period, compared with 53 patients (14.6%) during the IF evaluation period (*P* < .001). Forty patients (10.2%) were engaged with OUD treatment during the baseline period, compared with 59 patients (16.3%) during the IF evaluation period (*P* = .01). Patients in the IF evaluation period who received ED-initiated buprenorphine were more likely to be in treatment at 30 days (19 of 53 patients [35.8%]) than those who did not 40 of 309 patients (12.9%; *P* < .001). Additionally, there were increases in the numbers of ED clinicians with an X-waiver (from 11 to 196 clinicians) and ED visits with provision of buprenorphine (from 259 to 1256 visits) and naloxone (from 535 to 1091 visits).

**Conclusions and Relevance:**

In this multicenter effectiveness-implementation nonrandomized trial, rates of ED-initiated buprenorphine and engagement in OUD treatment were higher in the IF period, especially among patients who received ED-initiated buprenorphine.

**Trial Registration:**

ClinicalTrials.gov Identifier: NCT03023930

## Key Points

**Question:**

Does implementation facilitation (IF) increase adoption of emergency department (ED)–initiated buprenorphine and patient engagement in opioid use disorder treatment compared with grand rounds?

**Findings:**

In this nonrandomized hybrid type 3 effectiveness implementation trial conducted in 4 urban, academic EDs comparing 394 patients enrolled during a baseline evaluation period after grand rounds and 362 patients enrolled during an IF evaluation period, rates of ED-initiated buprenorphine and engagement in OUD treatment at 30 days were higher during the IF period.

**Meaning:**

These findings suggest that IF was associated with greater rates of ED-initiated buprenorphine and patient engagement in treatment at 30 days compared with grand rounds.

## Teaser

This hybrid type 3 effectiveness implementation trial evaluates whether provision of emergency department–initiated buprenorphine with outpatient referral for patients opioid use disorder is increased with an implementation facilitation strategy.

## Introduction

Opioid-associated deaths in the US exceeded 75 000 in the 12-month period ending October 2021.^1^ Emergency department (ED) visits related to opioid use disorder (OUD) have increased more than 100% in the past decade,^2^ especially during the COVID-19 pandemic.^3,4^ Despite evidence that methadone and buprenorphine reduce drug use, medical comorbidities, and mortality, most individuals with OUD remain untreated.^5,6^ Without treatment, ED patients with a nonfatal overdose are at increased risk of subsequent overdose and death, with a 1-year mortality rate near 5%^7^ and substantial mortality occurring within 1-month of the ED visit.^8^ Thus, there is a need to increase OUD treatment access, and the ED provides a unique setting to initiate OUD treatment and prevent opioid deaths.^9^

Our prior randomized clinical trial in patients with untreated OUD found that receiving ED-initiated buprenorphine with referral for ongoing buprenorphine in primary care increased the likelihood of addiction treatment engagement at 30 days compared with referral alone or a brief intervention with a facilitated referral.^10^ However, widespread adoption has lagged.^11^ Prior studies of implementation, predominately based on surveys, have reported a low level of readiness to provide buprenorphine among ED clinicians.^12,13,14^ One ED survey reported promising results using behavioral nudges to encourage the treatment of OUD but had a low response rate.^15^ Retrospective, observational ED studies, including academic and rural settings in the US and Canada, have demonstrated feasibility of initiating buprenorphine but did not include implementation outcomes.^16,17,18,19,20,21^

Therefore, we evaluated prospectively whether implementation facilitation (IF), an evidence-based implementation strategy,^22^ can increase adoption of ED-initiated buprenorphine with referral for ongoing OUD treatment (collectively referred to as ED-initiated buprenorphine) compared with traditional grand rounds didactic lectures.

## Methods

The full study protocol for this hybrid type 3 effectiveness-implementation nonrandomized trial was approved by the WCG IRB institutional review board, and reliance agreements were obtained locally at each site. A waiver of informed consent was granted by the institutional review board for ED and community clinician surveys and focus groups. Written consent was obtained for all patient participants. The original trial protocol is provided in Supplement 1; the trial protocol was amended, and the amended trial protocol and statistical analysis plan are provided in Supplement 2. This study is reported following the Consolidated Standards of Reporting Trials (CONSORT) reporting guideline.

### Overview

As described elsewhere,^23^ Project ED Health was a stepped-wedge hybrid type 3 implementation-effectiveness study (emphasizing implementation while collecting effectiveness data) conducted in 4 geographically diverse, urban, and academic EDs comparing grand rounds with IF to promote implementation of ED-initiated buprenorphine. The trial was initially planned as a modified stepped-wedge design, with sites sequentially crossing over to the IF period based on a randomly-assigned order. However the sites were engaged fully sequentially with no time overlaps between the sites or strategies (eFigure 1 in Supplement 3). We therefore adjusted to a pre-post study design comparing 2 evaluation periods: 12 months immediately following a grand rounds lecture (baseline evaluation period) and 12 months after completion of an intensive 6-month IF period (IF evaluation period). During each evaluation period, ED administrative record data and ED patient electronic health records (EHR) were extracted to assess the outcomes associated with the 2 evaluated implementation strategies. Observational cohorts of ED patients with OUD were enrolled during the 12-month evaluation periods at each site. The study timeline is shown in eFigure 1 in Supplement 3. The research was conducted between April 1, 2017, and November 30, 2020.

### Sites

Study ED sites, each with more than 60 000 annual visits, included Johns Hopkins Hospital in Baltimore, Maryland; Mount Sinai Beth Israel in New York, New York; University of Cincinnati Medical Center in Cincinnati, Ohio; and Harborview Medical Center in Seattle, Washington. The order of study sites engaging in and initiating the study protocol was randomized and blinded to all investigators.

### Implementation Strategies

#### Grand Rounds

The grand rounds intervention included a 60-minute in-person lecture attended by emergency medicine faculty, residents, and community clinicians, provided at each site (delivered by G.D.). The grand rounds lecture focused on (1) the opioid crisis in ED populations; (2) effective treatment of OUD, including ED-initiated buprenorphine; (3) harm reduction strategies, including dispensing or prescribing naloxone at discharge; and (4) importance of ED clinicians obtaining the DATA 2000 waiver (also known as an X-waiver) required to prescribe buprenorphine. The lecture was followed by a question-and-answer period. Specific implementation strategies were discussed, and online resources were shared.

#### Implementation Facilitation

To identify site-specific needs, lead study investigators (G.D., E.J.E., K.F.H., P.G.O., and D.A.F.) conducted mixed-methods formative evaluations,^24^ including surveys and focus groups with ED and community clinicians, and ED social workers and counselors, leadership, pharmacists, and patients with OUD, guided by the Promoting Action on Research Implementation in Health Services framework.^25,26,27^ Lead study investigators, as external facilitators, assisted and engaged local champions who tailored ED-initiated buprenorphine to each site. Continuing education, academic detailing, performance feedback, and program marketing were offered during an intensive 6 months of IF and continued throughout the 12-month IF evaluation period. Learning collaboratives were led by site champions, community partners, study investigators, and multidisciplinary national leaders in ED-initiated buprenorphine. Topics included development of clinical protocols, potential barriers and facilitators, and effective strategies for addressing stigma.

### Participants

#### ED and Community Clinicians and Leadership

ED participants included clinicians able to prescribe buprenorphine (ie, attending physicians, residents, advanced practice clinicians [APCs]), as well as nurses, counselors, social workers, pharmacists, and administrators. Community clinicians were physicians, APCs, administrators, counselors, and social workers from programs providing OUD treatment, including office-based practices and opioid treatment programs. All participants who had been employed at the given site for at least 6 months were invited to complete the surveys and participate in the focus groups.

#### Patients in Observational Cohorts

All ED patients were screened and recruited during rotating day and evening shifts by research associates. Potential participants who reported current opioid use completed an OUD structured diagnostic interview.^28^ Those with moderate to severe OUD were asked to provide a urine sample, which was analyzed with a rapid, point-of-care testing for opiates (eg, morphine, heroin), oxycodone, methadone, buprenorphine, fentanyl, amphetamine, methamphetamine, cocaine, benzodiazepines, barbiturates, 3,4-methylenedioxy-methamphetamine, and tetrahydrocannabinol. Patients who met all eligibility criteria provided written informed consent.

Patients were eligible if they were aged at least 18 years, met *Diagnostic and Statistical Manual of Mental Disorders* (*Fifth Edition*) (*DSM-5*) criteria for moderate-severe OUD,^10^ had an urine test result positive for opioids, and understood English. Because fentanyl testing was not routinely available and there was no fentanyl point-of-care rapid test approved by the US Food and Drug Administration, patients whose test results were exclusively positive for fentanyl and no other opioid were not eligible. Patients were ineligible if they were receiving OUD treatment in the past 30 days, were experiencing suicidal ideation, were cognitively impaired, presented from an extended care facility, or required opioids for pain or hospitalization. Patients enrolled during the baseline evaluation period could not enroll during the IF evaluation period.

### Outcomes

Outcomes were evaluated at the ED clinician and ED patient levels using objective and verifiable data. The primary implementation outcome of the observational cohorts study component, extracted from ED EHR data, was the rate of provision of ED-initiated buprenorphine (administered or prescribed) with referral for ongoing OUD treatment among patients enrolled during the baseline and IF evaluation periods. We hypothesized that IF, compared with grand rounds, will result in higher rates of ED-initiated buprenorphine with referral for ongoing OUD treatment. The primary effectiveness outcome, extracted from OUD treatment clinician and program clinical record data, was the rate of OUD treatment engagement on the 30th day after enrollment during the baseline and IF evaluation periods. OUD treatments consistent with the American Society of Addiction Medicine’s levels of care 1 to 4^29^ were considered in this outcome. We hypothesized that IF will result in higher rates of OUD treatment engagement on the 30th day after enrollment.

### ED Clinician–Level Data and Patient Assessments

Clinician level–data (physicians and APCs) for all patients receiving care in the EDs during the study period included administration and prescription of buprenorphine, the number of unique clinicians who administered or prescribed buprenorphine, and the number of ED visits during which naloxone was dispensed or prescribed. Data were extracted from each site’s EHR. ED leadership compiled lists of ED clinicians who obtained X waivers. Patient level–data for the observational cohorts included demographic and clinical characteristics, health status and health care utilization, and self-reported overdose events during 30 days prior to study enrollment, as well as their ED visit EHR data. Patient race and ethnicity was self-reported, categorized as American Indian or Alaskan Native, Asian, Black, Hispanic, Multiracial, White, and other, unknown, or refused. Race and ethnicity were assessed to characterize the cohorts of patients enrolled in the study.

### Statistical Power and Sample Size

Four geographically distinct urban, academic sites were selected to facilitate timely enrollment of the planned diverse sample size of 960 patients, 240 per site (120 enrolled during each of the evaluation periods). The planned sample size in the observational cohorts component was based on simulated data generated under a range of expected probabilities regarding hypothesized study outcomes and assuming potential intraclass correlations between 0 and 0.3 to achieve a statistical power greater than 0.80 to evaluate differences on the implementation and effectiveness primary outcomes.

### COVID-19 Disruption

Enrollment was suspended at 2 sites for 4 months in 2020. Study conduct followed COVID-19 research guidelines^30,31^ without protocol modifications. The planned total sample size of 960 patients in observational cohorts was not reached, primarily due to recruitment challenges at 1 site and the ED challenges associated with COVID-19.

### Statistical Analysis

ED administrative record data, including the numbers of X-waivered clinicians, ED visits with buprenorphine administered or prescribed, and ED visits with naloxone dispensed or prescribed during the baseline and IF evaluation periods were summarized descriptively. The 2 hypothesized outcomes of the observational cohorts study were analyzed using a logistic regression model evaluating the statistical significance of the differences between the baseline and IF evaluation periods and across the study sites using the LOGISTIC procedure in SAS statistical software version 9.4 (SAS Institute). As noted in the amended Statistical Analysis Plan in Supplement 2, we initially planned to conduct a generalized linear mixed model with fixed effects for evaluation period and calendar month and a random effect for site, given the stepped-wedge design with repeated measures for each block. However, given our modification to a pre-post study design, we adjusted our analytic approach to a logistic regression model with fixed effects for evaluation period and site.

Since a distinct data pattern emerged when analyzing the hypothesized IF effectiveness outcome, indicating that the effectiveness of IF was concentrated within the group of patients receiving ED-initiated buprenorphine during the IF evaluation period, an explanatory analysis of the differences in the rates of OUD treatment engagement on day 30 after enrollment between patients who received vs those who did not receive ED-initiated buprenorphine was conducted using a logistic regression.^32^ Finally, a time series analysis evaluating a linear effect of time instead of comparing 2 evaluation periods (baseline vs IF) using locally weighted running line smoother (LOESS) was conducted to evaluate whether potential other time trends in implementation of ED-initiated buprenorphine practices may have influenced the study outcomes independently of the IF strategies provided during the study. The amended research protocol and statistical analysis plan are available in Supplement 2. *P* values were 2-sided, and statistical significance was set at *P* = .05. Data were analyzed from July 16, 2021, to July 14, 2022.

## Results

### Implementation

Across all sites, grand rounds were attended by ED physicians, nurses, pharmacists, and community clinicians, including 165 ED attendings and residents. Five lead study investigators facilitated 10 site investigators (6 ED physicians and 4 community physicians) to engage 80 ED clinicians and 34 community individuals, including administrators, physicians, APCs, nurses, pharmacists, social workers, and counselors, through meetings, surveys, and 31 focus groups during the baseline evaluation period. Overall, eighteen 1-hour video-teleconferencing learning collaboratives were conducted between November 2018 and June 2020.

Each ED developed site-specific protocols, employed facilitators, and developed community partners for facilitated referrals.^33^ Facilitated referral included a day and time slot for follow-up with an office-based practice or OTP within 96 hours. A prescription was provided for daily buprenorphine until that appointment. Overdose education and naloxone distribution was included in all protocols, and clinicians were encouraged to obtain an X-waiver.

**Figure 1.**
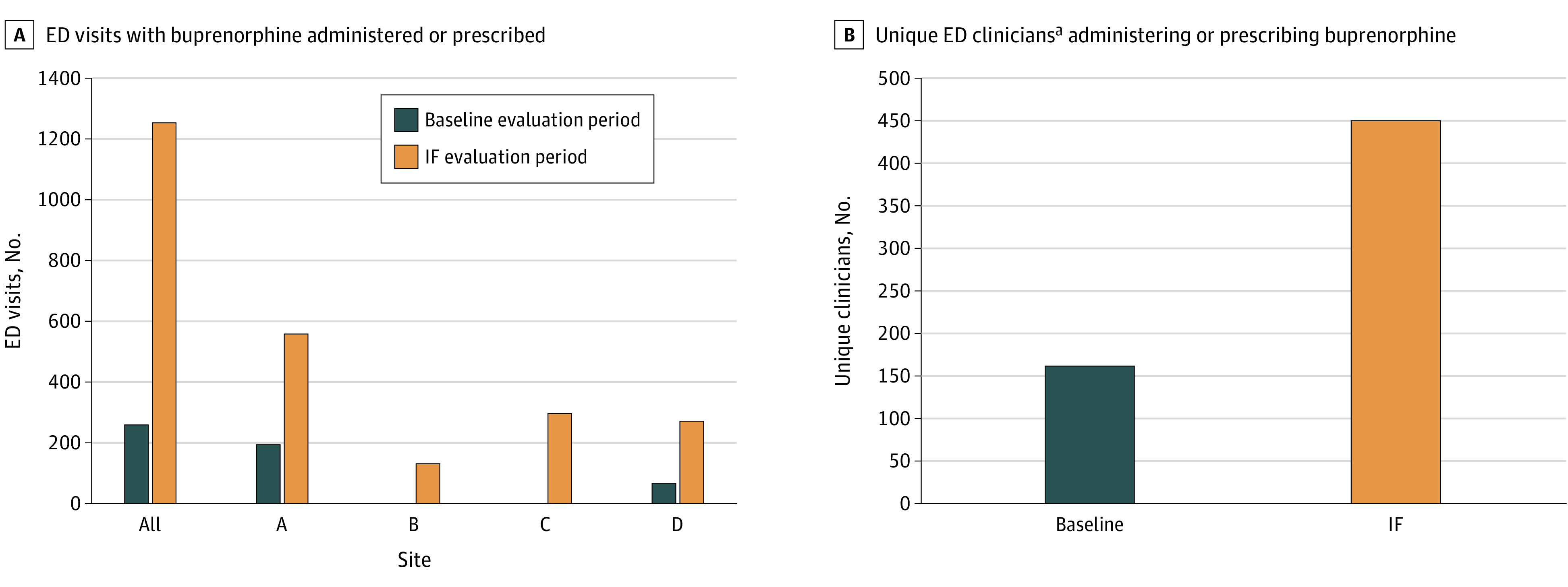
Number of Emergency Department (ED) Visits with Buprenorphine Administered or Prescribed Overall and by Site Data extracted from electronic health records. ^a^Includes attending physicians, residents, and advanced practice clinicians.

Across all sites, the number of ED clinicians with an X-waiver was higher in the IF period (196 clinicians; 161 physicians, 35 APCs) compared with the baseline period. (11 clinicians; 10 physicians, 1 APC). The IF period also had a higher number of ED visits at which buprenorphine was administered or prescribed (1256 vs 259, Figure 1), unique clinicians prescribing buprenorphine (449 vs 162), and number of ED visits with naloxone dispensed or prescribed (1091 vs 535). The LOESS plots (eFigure 2 and eFigure 3 in Supplement 3) indicate that there were no gradual over time changes during the baseline period, but sudden increases of ED-initiated buprenorphine during the IF period were observed.

### Observational Cohorts

**Figure 2.**
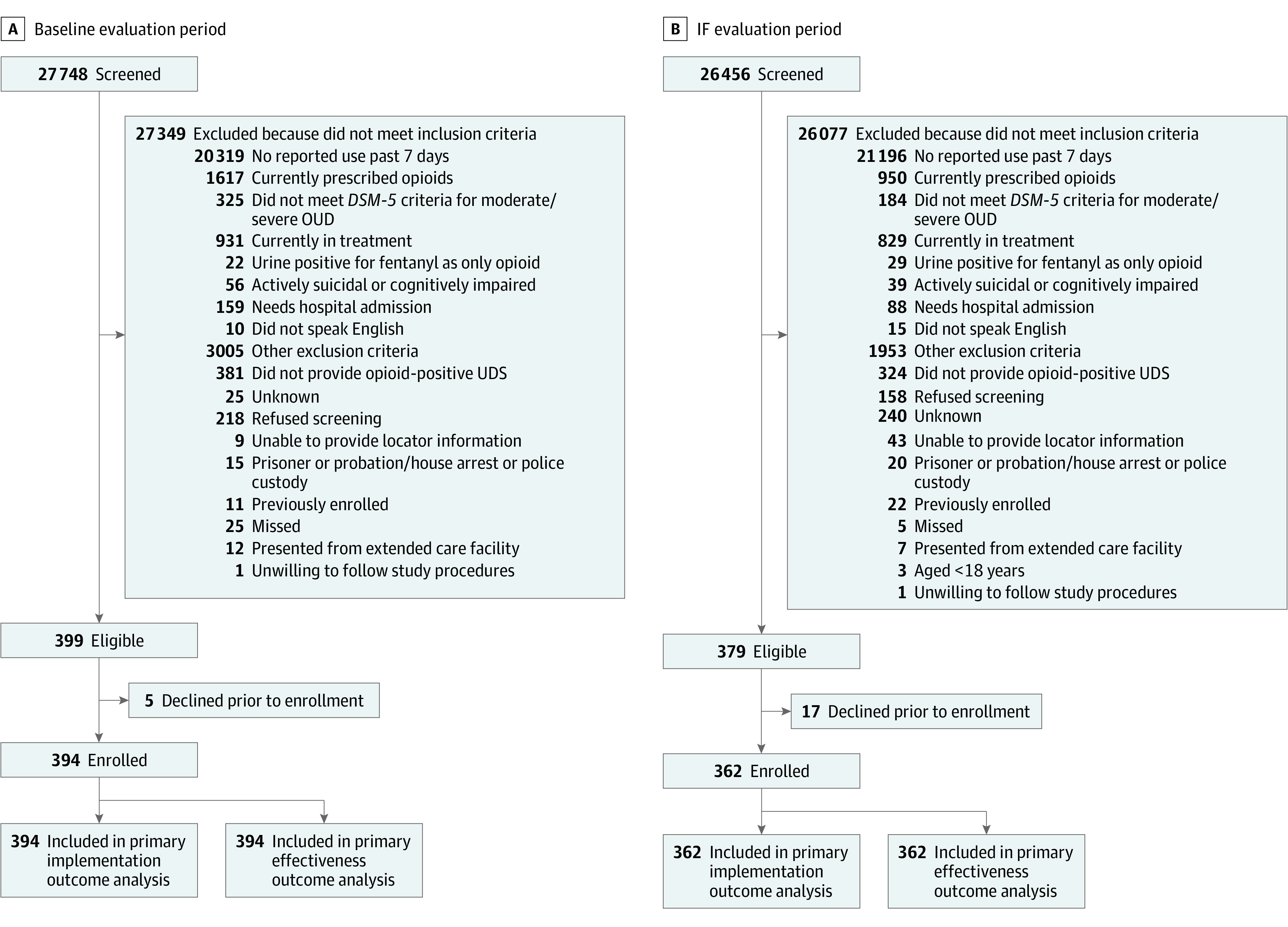
Trial Flowchart for the Baseline Evaluation Period

A total of 756 patients (mean [SD] age, 39.3 [11.7] years; 540 [71.4%] male patients) were enrolled across all sites. During the baseline evaluation period, 27 748 ED patients were screened universally during recruitment hours and 394 patients were enrolled and analyzed (Figure 2). During the IF evaluation period, 26 456 ED patients were screened and 362 patients were enrolled and analyzed. The Table lists the patients’ sociodemographic and clinical characteristics. The enrolled patients represented socioeconomically, racially, and ethnically diverse populations, including 223 Black patients (29.5%), 88 Hispanic patients (11.6%), and 394 White patients (52.1%); 420 patients (55.6%) were unemployed; and 431 patients (57.0%) reported unstable housing. A total of 214 patients (28.3%) reported having experienced an overdose in the past 30 days, with 164 patients (21.5%) requiring a medical intervention (Table). Most patients (541 patients [71.6%]) had Medicaid insurance, and 421 patients (55.7%) reported having no usual source of care or using the ED as their usual source of care. More than one-third of patients reported ever receiving psychiatric treatment, including 277 patients (36.6%) who received inpatient treatment and 335 patients (44.3%) who received outpatient treatment. The mean (SD) score for the Patient Depression Questionnaire 9-Item (PHQ-9) was 12.5 (7.2), consistent with moderate depression severity.^34^

**Table.** Baseline Demographic and Clinical Characteristics of Patients

Characteristic	Patients, No. (%)		
	Baseline evaluation period (n = 394)	IF evaluation period (n = 362)	Total (n = 756)
Sex			
Men	273 (69.3)	267 (73.8)	540 (71.4)
Women	121 (30.7)	95 (26.2)	216 (28.6)
Race			
American Indian or Alaskan Native	12 (3.0)	15 (4.1)	27 (3.6)
Asian	3 (0.8)	4 (1.1)	7 (0.9)
Black	110 (27.9)	113 (31.2)	223 (29.5)
Multiracial	25 (6.3)	12 (3.3)	30 (4.0)
White	206 (52.3)	188 (51.9)	394 (52.1)
Unknown or refused	47 (11.9)	24 (6.6)	71 (9.4)
Ethnicity			
Hispanic or Latino	49 (11.9)	39 (10.8)	88 (11.6)
Not Hispanic or Latino	345 (87.6)	323 (89.2)	668 (88.4)
Age, mean (SD) y	38.9 (11.7)	39.7 (11.7)	39.3 (11.7)
Education			
<High school diploma	140 (35.5)	126 (34.8)	266 (35.2)
High school graduate or equivalent	130 (33.0)	146 (40.3)	276 (36.5)
Some college, no degree	74 (18.8)	62 (17.1)	136 (18.0)
≥College degree	50 (12.7)	28 (7.7)	78 (10.3)
Employment			
Working	63 (16.0)	66 (18.2)	129 (17.1)
Unemployed	228 (57.9)	192 (53.0)	420 (55.6)
Disabled (permanently or temporarily)	80 (20.3)	62 (17.1)	142 (18.8)
Other	23 (5.8)	42 (11.6)	65 (8.6)
Unstable housing			
Spent ≥1 night in the past 12 mo^a^			
Any unstable housing or homelessness	275 (69.8)	262 (72.4)	537 (71.0)
Shelter for persons experiencing homelessness	102 (25.9)	108 (29.8)	210 (27.8)
On the street or public place not intended for sleeping	191 (48.4)	192 (53.0)	383 (50.7)
Welfare hotel or SRO	43 (10.9)	48 (13.3)	91 (12.0)
Doubled-up in someone else’s house or apartment	190 (48.2)	135 (37.3)	325 (42.9)
Emergency, temporary, transitional, or halfway house	36 (9.1)	35 (9.7)	71 (9.4)
Currently living in^a^			
Any unstable housing or homelessness	222 (56.3)	209 (57.7)	431 (57.0)
Shelter for persons experiencing homelessness	41 (10.4)	41 (11.3)	82 (10.8)
On the street or public place not intended for sleeping	94 (23.9)	98 (27.1)	192 (25.4)
Welfare hotel or SRO	3 (0.8)	6 (1.7)	9 (1.2)
Emergency, temporary, transitional, or halfway house	4 (1.0)	9 (2.5)	13 (1.7)
Doubled-up in someone else’s house or apartment	102 (25.9)	77 (21.3)	179 (23.7)
Health insurance			
Any	344 (87.3)	296 (81.8)	640 (84.7)
Private or commercial	45 (11.4)	24 (6.6)	69 (9.1)
Medicare	31 (7.9)	37 (10.2)	68 (9.0)
Medicaid	287 (72.8)	254 (70.2)	541 (71.6)
Other	7 (2)	6 (2)	13 (2)
Has a primary medical practitioner, No./total No. (%)	149/393 (37.9)	131/361 (36.3)	280/75 (37.0)
Usual source of care, No./total No. (%)			
Private physician office	57/393 (14.5)	59/361 (16.3)	116/754 (15.3)
Clinic	116/393 (29.5)	96/361 (26.6)	212/754 (28.0)
ED or none	217/393 (55.2)	204/361 (56.5)	421/754 (55.7)
Positive urine test results during enrollment			
Opiates	328 (83.2)	283 (78.2)	611 (80.8)
Fentanyl	200 (50.8)	216 (59.7)	416 (55.0)
Oxycodone	159 (40.4)	139 (38.4)	298 (39.4)
Methadone	43 (10.9)	56 (15.5)	99 (13.1)
Buprenorphine	144 (36.5)	169 (46.7)	313 (41.4)
Cocaine	177 (44.9)	165 (45.6)	342 (45.2)
Methamphetamine	146 (37.1)	141 (39.0)	287 (38.0)
Amphetamine	114 (28.9)	102 (28.2)	216 (28.6)
Benzodiazepines	101 (25.6)	126 (34.8)	227 (30.0)
Barbiturates	7 (1.8)	1 (0.3)	8 (1.1)
Tetrahydrocannabinols	153 (38.8)	128 (35.4)	281 (37.2)
MDMA	21 (5.3)	26 (7.2)	47 (6.2)
Overdose history, No./total No. (%)			
≥1 Overdose in the past 30 d	108/393 (27.5)	106/361 (29.3)	214/754 (28.3)
Report a medical intervention overdose in past 30 d	84/393 (21.4)	79/361 (21.8)	163/754 (21.5)
Injection drug use reported in past month	232/394 (58.9)	185/361 (51.2)	417/755 (55.1)
Mental health history			
Psychiatric evaluation during the ED visit	11/394 (2.8)	15/361 (4.2)	26/755 (3.4)
Lifetime psychiatric treatment			
Inpatient	161/392 (40.9)	116/361 (32.1)	277/753 (36.6)
Outpatient	185/393 (47.0)	150/361 (41.6)	335/754 (44.3)
Any psychiatric treatment in the past 30 d	60/393 (15.2)	42/361 (11.6)	102/754 (13.5)
PHQ-9 score, mean (SD)^b^	12.9 (6.9)	12.1 (7.4)	12.5 (7.2)

Abbreviations: ED, emergency department; IF, implementation facilitation; MDMA, 4-methylenedioxy-methamphetamine; PHQ-9, Patient Depression Questionnaire 9-Item; SRO, single room occupancy.

^a^ Multiple responses could be given.

^b^ The PHQ-9 is the major depressive disorder module of the full Patient Health Questionnaire.

**Figure 3.**
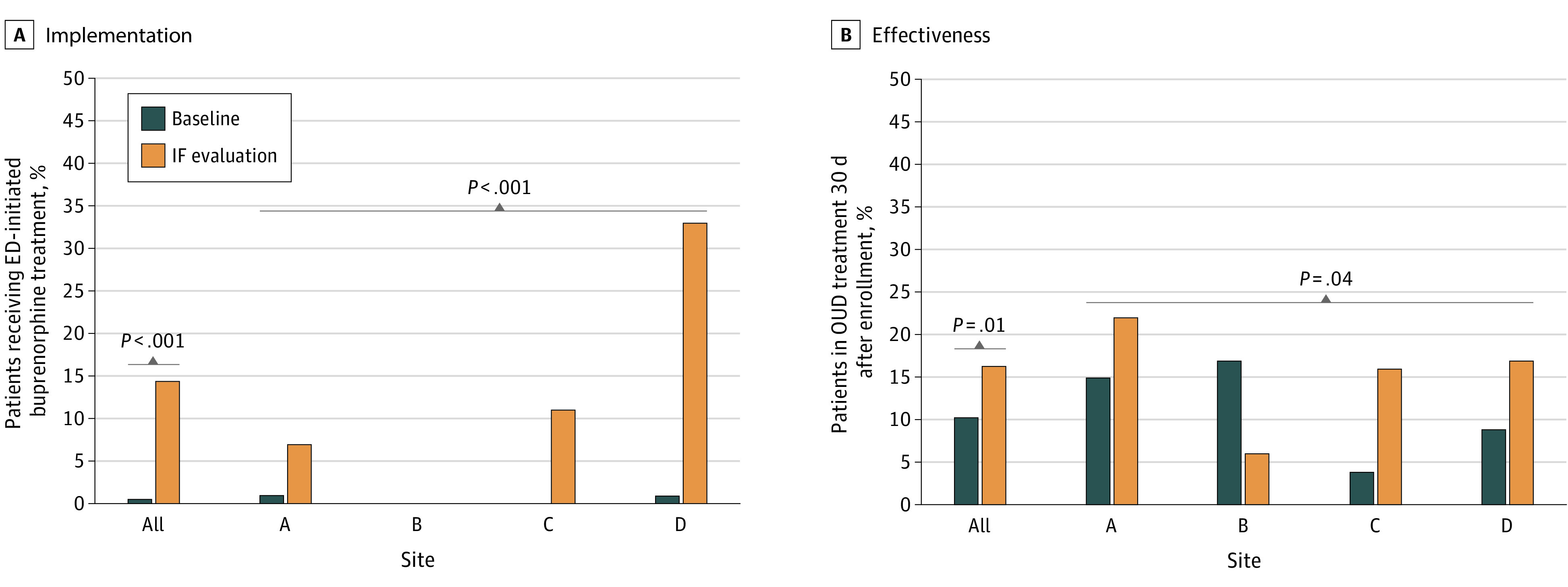
Observational Outcomes in Implementation and Effectiveness of Emergency Department (ED)–Initiated Buprenorphine Strategies IF indicates implementation facilitation; OUD, opioid use disorder.

In these enrolled ED cohorts, provision of ED-initiated buprenorphine was higher in the IF period (53 patients; 14.6%; 95% CI, 11%-18%) compared with the baseline period (2 patients, 0.5%; P <.001). From the baseline to the IF evaluation periods, site-specific rates of ED-initiated buprenorphine were 1 of 104 patients (1.0%) to 7 of 94 patients (7.6%) at site A, 0 of 42 patients to 0 of 65 patients at site B, 0 of 120 patients to 11 of 98 patients (11.2%) at site C, and 1 of 128 patients (0.8%) to 35 of 105 patients (33.3%) at site D. The overall site effect was statistically significant (*P* < .001). No changes in provision of ED-initiated buprenorphine were observed at site B, and rates of ED-initiated buprenorphine increased most at site D (Figure 3A).

The rates of engagement in OUD treatment on day 30 after enrollment were higher during the IF period (59 of 362 patients; 16.3%) compared with the baseline period (40 of 394 patients; 10.2%, P=.01) From the baseline to the IF evaluation periods, site-specific rates of 30-day OUD treatment engagement were 16 of 104 patients (15.4%) to 21 of 94 patients (22.3%) at site A, 7 of 42 patients (16.7%) to 4 of 65 patients (6%) at site B; 5 of 120 patients (4.2%) to 16 of 98 patients (16.3%) at site C; and 12 of 128 patients (9.4%) to 18 of 105 patients (17.1%) at site D. The overall site effect was statistically significant (*P* = .04). Higher rates of engagement occurred at sites A, C, and D, while lower rates of patient engagement in treatment was observed at site B (Figure 3B).

**Figure 4.**
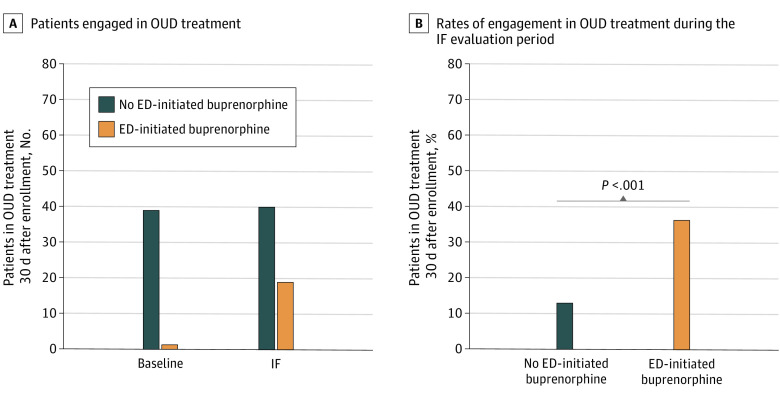
Effectiveness of Emergency Department (ED)–Initiated Buprenorphine for Opioid Use Disorder (OUD) Treatment

The increase in the rates of engagement in OUD treatment at 30 days occurred predominantly among patients who received ED-initiated buprenorphine intervention (Figure 4A), and patients enrolled during the IF evaluation period were significantly more likely to be in treatment at 30 days if they received ED-initiated buprenorphine (19 of 53 patients [35.8%]) than if they did not (40 of 309 patients [12.9%]; *P* < .001) (Figure 4B). Site-specific rates of engagement in OUD treatment for patients who received ED-initiated buprenorphine vs those who did not were 3 of 7 patients (42.9%) vs 18 of 87 patients (20.7%) at site A, 0 patients vs 4 of 65 patients (6.1%) at site B, 3 of 11 patients (27.3%) vs 13 of 87 patients (14.9%) at site C, and 13 of 35 patients (37.1%) vs 5 of 70 patients (7.1%) at site D. The overall site effect was not statistically significant (*P* = .06).

## Discussion

This hybrid type 3 effectiveness-implementation nonrandomized trial is the first multisite study, to our knowledge, to apply and prospectively evaluate a robust multicomponent implementation strategy, IF, aimed at increasing the adoption of ED-initiated buprenorphine in 4 geographical regions of the US. We observed that IF strategies were associated with greater uptake of ED-initiated buprenorphine than the traditional educational grand rounds presentation. The numbers of X-waivered ED clinicians, ED visits with buprenorphine administered or prescribed, unique clinicians prescribing buprenorphine, and ED visits with naloxone dispensed or prescribed were higher in the IF evaluation period at all sites, based on EHR data. In the observational cohorts, enrolled patients were diverse in gender, race, ethnicity, and socioeconomic status, with most reporting unstable housing and use of the ED as their usual access to care. While the proportion of patients receiving ED-initiated buprenorphine in the enrolled observational cohorts during the IF evaluation period was modest, those who did receive ED-initiated buprenorphine were more likely to be engaged in OUD treatment 30 days later compared with patients who did not receive ED-initiated buprenorphine. This finding suggests that further emphasis on increasing adoption of ED-initiated buprenorphine could be beneficial to improving patient outcomes.

While all sites integrated their buprenorphine protocols into care pathways and increased the number of patients administered or prescribed buprenorphine, as evidenced by data extracted from the ED EHR, there were notable between-site differences in the observational cohorts. No change in the rate of ED-initiated buprenorphine provision was observed at site B, and site D had the greatest increase in ED-initiated buprenorphine. Site B did not achieve their planned enrollment, possibly due to their proximity to a large methadone treatment center and high rates of intermittent use of methadone in the community, which may have precluded eligibility for ED buprenorphine initiation. Of note, clinicians at site B did administer or prescribe buprenorphine in 131 ED visits not included in the observation cohort enrollments during the IF evaluation period, compared with 0 at baseline. Thus, there was adoption of buprenorphine initiation by clinicians. Site D was the last IF site and may have benefited from the improved IF delivery resulting from participating in learning collaboratives and accumulated experiences and practice at the earlier sites.

Our previously published reports on barriers and facilitators of ED-initiated buprenorphine,^24^ including information gleaned from patient perspectives,^35^ discovered during our formative evaluation were and are critical to the success of facilitating practice change. These comprehensive strategies, including recruitment of champions, eliciting buy-in from departmental and institutional leadership, establishment of community partners for follow-up care, and protocol development informed by existing staff at all levels tailored to individual sites, are of great value to EDs in the early adoption phase and are associated with increased likelihood of adoption of ED-initiated buprenorphine.^24^

The rates of adoption of ED-initiated buprenorphine reported in this study may have been influenced over time by professional and organization guidelines and reports, such as the 2019 National Academy of Medicine consensus report on medications for OUD^5^ and the American College of Emergency Physicians (ACEP) 2021 consensus recommendations.^36^ However, findings from a concurrent study of large numbers of EDs, the ACEP E-QUAL Opioid Initiative Network,^37^ suggest that without specific implementation strategies, the number of patients receiving ED-initiated buprenorphine will continue to be low. An assessment of ED capabilities conducted in 300 small community and rural EDs found that only 6.3% of EDs reported having an ED-initiated buprenorphine protocol and 9.7% of EDs were in the process of developing a protocol, highlighting the need for implementation strategies to support practice change.^37^

ED-initiated buprenorphine is a relatively new practice compared with the 2 decades that the medication has been available to treat OUD. Implementation likely requires strategies at the clinician, patient, health care system, and regulatory levels, and our findings suggest adoption would benefit from additional interventions, such as mandated performance measures. ED clinicians respond to quality benchmarks and critical review for time-sensitive and life-saving interventions, such as door to reperfusion times for myocardial infarction.^38^ Adoption may also benefit from new resources, such as MDCalc,^39^ and the BUP initiation application,^40^ that integrate diagnostic questionnaires and treatment algorithms available on smart phones and embedded in EHR clinical decision tools.^41,42^ A 2022 study^43^ that investigated a clinical decision support tool for ED-initiated buprenorphine embedded in an EHR without other implementation strategies did not find an increase in patient rates of initiating buprenorphine but did find an increase in the number of physicians who initiated buprenorphine in the ED.

In addition, the use of long-acting formulations of buprenorphine and removal of barriers, such as insurance preauthorization, may impact the effectiveness of ED-initiated buprenorphine.^43^ Removal of regulatory barriers, such as the recent removal of the requirement for an X-waiver,^44^ may also improve adoption. Recently published studies retrospectively evaluating the implementation of ED-initiated buprenorphine have reported promising outcomes associated with use of active navigation^45^ and peer recovery specialists,^46^ highlighting that increases in adoption of ED-initiated buprenorphine would require additional strategies at the individual, institutional, and policy levels. Finally, programs addressing the social determinants of health, such as housing, medication payment, and logistical help for transportation to pharmacies, may improve the implementation and effectiveness of ED-initiated buprenorphine. Future implementation of ED-initiated buprenorphine programs may benefit from new technologies and regulatory changes that reduce barriers to OUD care to improve quality of care and save lives.

### Limitations

This study has some limitations. The study did not reach the planned sample size in the observational cohorts, and inclusion of only 4 sites limits generalizability of the findings. The study design cannot fully disentangle potential time trends in implementation of ED-initiated buprenorphine practices independently of the IF strategies provided during the study. However, time series analyses indicate that there was no change over time during the baseline period, but substantially higher rates during the IF period. This provides additional support for our primary analytical model results. While the increase in the number of individuals receiving ED-initiated buprenorphine in the observational cohorts was modest in the IF period, the study did not collect information on the number of patients who refused buprenorphine in the ED. Refusal of ED-initiated buprenorphine when offered by the ED clinicians could have resulted in the overall modest rates of receiving this intervention.

## Conclusions

The findings of this hybrid type 3 effectiveness-implementation nonrandomized trial suggest that multicomponent IF strategies were associated with increased rates of ED-initiated buprenorphine. Furthermore, patients receiving this intervention were engaged in continued OUD treatment at higher rates than those who did not receive ED-initiated buprenorphine at 30 days after initiation.

## Notes

Supplement 1.
Trial Protocol

Supplement 2.
Amended Trial Protocol and Statistical Analysis Plan

Supplement 3. **eFigure 1.** Study Timeline **eFigure 2.** Time Series Analysis Plot Across All Sites **eFigure 3.** Time Series Analysis Plots for Each Study Site

Supplement 4.
Data Sharing Statement
