# Supplementary material for: Implementation Facilitation to Promote Emergency Department–Initiated Buprenorphine for Opioid Use Disorder
Source: JAMA Netw Open. 2023 Apr 5;6(4):e235439. doi: 10.1001/jamanetworkopen.2023.5439 (PMC10077107; doi:10.1001/jamanetworkopen.2023.5439)
Supplement: Supplement 1. — Trial Protocol [file jamanetwopen-e235439-s001.pdf]

Protocol CTN 0069, v 3.0 to v 4.0		
Protocol Section # and Title	Description of Changes	Change Category/Rationale
Staffing	Addition of Dr. Kathryn Hawk to the list of Co-Investigator staff.<br><br>Addition of Casey Nelson and Kayla Chaney to list of NIDA CTN Clinical Coordinating Center and Data and Statistics Center staff.<br><br>Removal of Dr. Kathryn Hawk from the list of Executive Committee staff.	Minor
1.0 List of Abbreviations	Addition of "ED Visit Review (EDR)"	Minor – not previously added
4.4.1 Exploratory Aims	Removal of "as well as clinical FTE" from implementation section.	Minor – clinical FTE no longer being collected
6.4 ED Providers	Revision of the following text, "Due to pending legislation, it is conceivable that nurse practitioners (NPs) and physician assistants (Pas) may become eligible to prescribe under DATA 2000."<br><br>New text, "We will include all ED providers credentialed to practice in the site ED that are capable of prescribing BUP. This includes physicians, nurse practitioners (NPs) and physician assistants (PAs).[23,24]	Minor - legislation approved to allow NPs and PAs to prescribe BUP
7.2 Exclusion Criteria (criterion #9)	Addition of the following text, "Note: A patient may NOT enroll in both the baseline evaluation and IF evaluation periods."	Minor - added for further clarification
8.5 Exploratory Outcomes	Removal of "as well as clinical FTE" from implementation section.	Minor – clinical FTE no longer being collected

Protocol CTN 0069, v 3.0 to v 4.0		
Protocol Section # and Title	Description of Changes	Change Category/Rationale
9.1 Screening and Informed Consent Procedures Baseline and IF Evaluation Periods	Addition of the following text in the section title, “Baseline and IF Evaluation Periods”	Minor – added for further clarification
9.1.1 Screening	“Enrollment period” changed to “evaluation period”<br><br>“Potential study patients” changed to “Patients”<br><br>Revision of the following text, “Potential study patients who report any opioid use in the past month will complete a brief (10 minute) structured diagnostic interview (DSM-5) to evaluate for the presence of moderate/severe OUD.”<br><br>New text, “Potential study patients who report any opioid use in the past month will complete a 7-day TLFB. If opioid use is reported during the past 7 days a brief (10 minute) structured diagnostic interview (DSM-5) to evaluate for the presence of moderate/severe OUD is administered.”	Minor – changed for consistency<br><br>Minor – changed for consistency<br><br>Minor – added for further clarification
9.1.2 Informed Consent Procedures (Patient Participants)	Previous protocol version indicates that sites will request IRB approval to use a compound authorization form to serve as a combined consent and HIPAA disclosure form. This section update updated to say that each site **will** utilize the IRB-approved compound authorization form.	Minor – changed to reflect approval

Protocol CTN 0069, v 3.0 to v 4.0		
Protocol Section # and Title	Description of Changes	Change Category/Rationale
9.4 Compensation	Addition of the following text, "…but may be completed as a telephone visit if a participant is unable to attend visit. A urine sample will not be collected if the visit is completed via telephone. "	Minor – added for further clarification
10.1.1 Table 2	"Patient Participant Eligibility Summary" changed to "Patient Eligibility Summary"<br><br>"Written Informed Consent" changed to "Written Compound Informed Consent"	Minor – changed to reflect form name update<br><br>Minor – changed for consistency
10.2.4 Patient Eligibility Summary and Compound Informed Consent and Inclusion/Exclusion	"Patient Participant Eligibility Summary" changed to "Patient Eligibility Summary"<br><br>"Participants" changed to "patients"	Minor – changed to reflect form name update<br><br>Minor – changed for consistency
10.2.16 ED Visit Review	Revision of the following text, "The ED Visit Review form collects information about the index ED visit. It is completed by the RA without patient participant input."<br><br>New text, "The ED Visit Review (EDR) form collects information about study and clinical processes surrounding the index ED visit. It is completed by the Senior RA without patient input, in part by performing a medical record abstraction. EDR data may be independently reviewed for accuracy. "	Minor – added for further clarification
10.2.17 Critical Action Checklist	Addition of the following text, "…by a site staff member that is blinded to the index visit."	Minor – added for further clarification

Protocol CTN 0069, v 3.0 to v 4.0		
Protocol Section # and Title	Description of Changes	Change Category/Rationale
10.2.20 Adverse Events (AEs) and Serious Adverse Events (SAEs)	Addition of the following text, “Only death events that occur within the 30-day follow-up period will be recorded on the AE/SAE form set.”	Minor – added for further clarification
11.2.1 Overview of the Implementation Facilitation	Addition of the following text, “The LN will modify the Kirchner manual during the conduct of the study based on their facilitation experiences.”	Minor – added for further clarification
11.4 Qualitative Data Collection and Analysis	Timeframe for second set of focus groups changed from month 4 to approximately months 4 – 5.<br><br>Addition of the following text, “However, data collected during focus groups and during the formative evaluation is part of an iterative process, and therefore additional focus groups, one-on-one phone interviews and email correspondence take place as needed.”<br><br>Added “approximately” to the number of focus group attendees and number of focus group events.	Minor – added to broaden timeframe; does not affect outcomes<br><br>Minor – added for further clarification<br><br>Minor – added to broaden focus group composition; does not affect outcomes
11.6 Verbal Consent for Focus Group Participants	“ED providers” changed to “ED and Community providers”<br><br>Addition of the following text, “Focus group participants will also be made aware that they will complete a brief demographic survey. “	Minor – added for further clarification<br><br>Minor – added for further clarification
11.7 Conduct of Focus Groups	“Dr. Edelman in conjunction with Drs. D’Onofrio and Fiellin…” changed to “Trained and qualified personnel…”	Minor – changed to allow for other qualified personnel to lead focus groups

Protocol CTN 0069, v 3.0 to v 4.0		
Protocol Section # and Title	Description of Changes	Change Category/Rationale
11.10 Additional Components of Implementation Facilitation	Drs. Edelman, D'Onofrio and Fiellin's names replaced by "the external facilitators in collaboration with trained and qualified personnel."	Minor – changed to allow for other qualified personnel to assist with implementation facilitation
12.12 Interim Monitoring	Revision of the following text, "However, a power and sample size recalculation will be performed at the end of year one based on the primary outcome rates observed in the baseline evaluation period."<br><br>New text, "However, a power and sample size recalculation will be performed based on the primary outcome rates and enrollment rates observed in the baseline evaluation period. The power and sample size recalculation will be done no earlier than the end of the first site's baseline evaluation period."	Minor – changed to reflect current practice
12.15 Exploratory Aims	Removal of "and clinical FTE" from implementation section.	Minor – clinical FTE not being collected
13.0 Training	Drs. Edelman, D'Onofrio, Hawk and O'Connor names replaced by "trained personnel"	Minor – changed to allow for other qualified personnel to assist with provider training
14.4 Informed Consent	Text revised to reflect approval of IRB waiver for written informed consent.	Minor – changed to reflect approval
14.8.1 Data collection and data completeness monitoring	Removed the requirement for research assistants to access medical records within 72 hours for the index visit.	Minor – changed to allow adequate time for submission and review of the medical record
14.12 Adverse Events (AEs) and Serious Adverse Events (SAEs)	Addition of the following text, "Only death events that occur within the 30-day follow-up period will be recorded on the AE/SAE form set."	Minor – added for further clarification

To be used to copy and paste text for IRB submission only.

COPY

NIDA CTN PROTOCOL 0069

Opioid Use Disorder in the Emergency Department (ED-HEALTH)

Version 4.0

May 22, 2018

Lead Investigator (LI):

Gail D'Onofrio, MD, MS
New England Consortium Node
Department of Emergency Medicine,
Yale School of Medicine

Co-Lead Investigator (Co-LI):

David A. Fiellin, MD
New England Consortium Node
Department of Internal Medicine
Yale School of Medicine

Co-Investigators (CI):

Yale University
Marek Chawarski, PhD
Departments of Psychiatry & Emergency Medicine

E. Jennifer Edelman, MD, MHS
Department of Internal Medicine

Kathryn Hawk, MD, MHS
Department of Emergency Medicine

Patrick O'Connor, MD
Department of Internal Medicine

Michael Pantalon, PhD
Department of Emergency Medicine

Susan Busch, PhD
School of Public Health

Leslie Curry, PhD
School of Public Health

Project Director:

Patricia Hansen Owens, MS
Department of Emergency Medicine

Assistant Project Director:

Shara Martel, MPH
Department of Emergency Medicine

CCTN Protocol Coordinators:

Kristen Huntley, PhD
David Liu, MD

NIDA CTN Clinical Coordinating Center and Data and Statistics Center:

Eve Jelstrom, CRNA, MBA
CCC Co-Principal Investigator

Dagmar Salazar, MS
CCC Protocol Specialist

Kayla Chaney
CCC Protocol Specialist

Mat Makowski, PhD
DSC Statistician

Lauren Yesko
DSC Project Director

Casey Nelson, MPH
DSC Data Manager

COPY

Executive Committee:

Roger D. Weiss, MD
New England Consortium Node
Harvard Medical School

Kathleen Carroll, PhD
New England Consortium Node
Yale School of Medicine

Dennis Donovan, PhD
Pacific Northwest Node
University of Washington

John Rotrosen, MD
New York Node
New York University

Ryan P. McCormack, MD
New York Node
NYU Langone Medical Center

COPY

TABLE OF CONTENTS

1.0 LIST OF ABBREVIATIONS 9
2.0 STUDY SYNOPSIS 11
2.1 Primary Implementation and Effectiveness Aims 12
3.0 INTRODUCTION 13
3.1 Background and Significance 13
3.2 Innovation 13
3.3 Public Health Impact 14
3.4 Sustainability 14
4.0 AIMS, OBJECTIVES & HYPOTHESES 15
4.1 Overview 15
4.2 Primary Aims 15
4.3 Secondary Aims and Hypothesis 15
4.3.1 Implementation Aims 15
4.3.2 Implementation Hypotheses 16
4.3.3 Effectiveness Aims (at 30-day follow-up assessment) 17
4.3.4 Effectiveness Hypotheses (at 30-day follow-up assessment) 17
4.4 Exploratory Aims 17
4.4.1 Study Patient, Emergency Department and Provider Characteristics 17
4.4.2 Cost effectiveness 18
5.0 STUDY DESIGN 19
5.1 Figure 1: Overview: Study Activities 19
5.2 Implementation Facilitation Leads to Practice Change 20
5.2.1 Table 1: Components of Implementation Facilitation 21
5.3 Duration of Study 21
5.3.1 Study Timeline Estimated Dates 21
6.0 STUDY SETTINGS 22
6.1 Number of ED Sites 22
6.2 ED Characteristics 22
6.3 Rationale for ED Criteria 23
6.4 ED Providers 23
6.5 Community Opioid Treatment Providers and Programs 23
6.6 Rationale for Community Opioid Treatment Providers and Program Criteria 23

COPY

7.0 STUDY POPULATION 24
7.1 Inclusion Criteria 24
7.2 Exclusion Criteria 24
7.3 Feasibility 25
8.0 OUTCOME MEASURES 26
8.1 Primary Implementation Outcome Measure 26
8.2 Primary Effectiveness Outcome Measure 26
8.3 Secondary Implementation Outcome Measures 26
8.4 Secondary Effectiveness Outcome Measures 27
8.5 Exploratory Outcomes 27
9.0 STUDY PROCEDURES 29
9.1 Screening and Informed Consent Procedures Baseline and IF Evaluation Periods 29
9.1.1 Screening 29
9.1.2 Informed Consent Procedures (Patient Participants) 29
9.1.3 Eligibility Confirmation and Enrollment 30
9.2 Participant Withdrawal 30
9.3 Participant 30-day Follow-up Assessments 30
9.4 Compensation 30
10.0 STUDY PATIENT ASSESSMENTS 31
10.1 Baseline Evaluation and IF Evaluation Periods 31
10.1.1 Table 2: Schedule of Study Activities and Assessments (Study Patient) by Study Time Period 32
10.2 Study Patient Participant Assessments 33
10.2.1 ED Health Quiz 33
10.2.2 DSM-5 33
10.2.3 Urine Drug Screen (Toxicology Testing) 33
10.2.4 Patient Eligibility Summary and Compound Informed Consent and Inclusion/Exclusion 33
10.2.5 Demographics and Additional Characteristics 33
10.2.6 Locator Information Form 33
10.2.7 Other Substance Use 34
10.2.8 Timeline Follow-back (TLFB) 34
10.2.9 Health Services Utilization Inpatient and Health Services Utilization Outpatient [29] 34
10.2.10 Health Status 34

COPY

10.2.11 Overdose Events 34
10.2.12 EuroQol (EQ-5D) 34
10.2.13 Crime and Criminal Justice 34
10.2.14 Healthcare Visit Logistics 35
10.2.15 Engagement in Treatment Survey 35
10.2.16 ED Visit Review 35
10.2.17 Critical Action Checklist 35
10.2.18 ED Visits and Hospitalizations 35
10.2.19 Study Completion 35
10.2.20 Adverse Events (AEs) and Serious Adverse Events (SAEs) 35
10.2.21 Protocol Deviation Form 36
11.0 IMPLEMENTATION FACILITATION (IF) STUDY PROCEDURES 37
11.1 Standard Dissemination Practice - Education 37
11.2 Implementation Facilitation 37
11.2.1 Overview of the Implementation Facilitation 37
11.2.2 Formative Evaluation 37
11.3 Quantitative Data Collection and Analysis 38
11.4 Qualitative Data Collection and Analysis 39
11.5 Development of Focus Group Questions 40
11.6 Verbal Consent for Focus Group Participants 40
11.7 Conduct of Focus Groups 40
11.8 Administration of BUP During IF Evaluation Period 41
11.9 Referral for Continuation of MAT 41
11.10 Additional Components of Implementation Facilitation 41
11.11 Provider Intervention to Enhance Motivation for Treatment 44
12.0 STATISTICAL ANALYSES 45
12.1 Definition of the Primary Effectiveness Outcome 45
12.2 Definition of the Primary Implementation Outcome 45
12.3 Experimental Design 45
12.4 Recruitment and Enrollment 46
12.5 Hypothesized Treatment Effect Sizes 46
12.5.1 Table 3: Hypothesized Probabilities of Success 46
12.6 Power Analyses to Justify Sample Size 46
12.6.1 Table 4: Scenarios Used to Simulate Power 47

COPY

12.7 Potential for Sites to have Baseline Probability of 0 for Primary Implementation Outcome......47
12.7.1 Table 5: List of Figures and their Contents for the Power Analysis......48
12.8 ChangeToOne Approach......48
12.8.1 Figure 2: ChangeToOne Power Analysis; Alternative Cases......49
12.8.2 Figure 3: ChangeToOne Confidence Intervals for p1, p2; Alternative Cases......50
12.8.3 Figure 4: “ChangeToOne” test size; Null Cases......50
12.8.4 Figure 5: “ChangeToOne” Confidence Intervals, p1, p2; Null Cases......51
12.9 Bayesian Approach......51
12.9.1 Summary of Power Simulations......51
12.10 Missing Data......51
12.11 Controlling Type 1 Error in Primary Analyses......52
12.12 Interim Monitoring......52
12.13 Planned Secondary Analyses......52
12.14 Statistical Methods......53
12.14.1 Primary Outcome Analysis......53
12.14.2 Effect Modification of Primary Analysis......54
12.14.3 Secondary Implementation Outcomes......54
12.14.4 Effectiveness Outcomes......55
12.15 Exploratory Analyses......56
13.0 TRAINING......58
14.0 REPORTING AND MONITORING......59
14.1 Statement of Compliance......59
14.2 Regulatory Files......59
14.3 Research Advisory Panel of California (California sites only)......59
14.4 Informed Consent......59
14.5 Health Insurance Portability and Accountability Act (HIPAA)......60
14.6 Investigator Assurances......60
14.7 Financial Disclosure......60
14.8 Clinical Monitoring......61
14.8.1 Data collection and data completeness monitoring......61
14.9 Study Documentation......62
14.10 Safety Monitoring......62
14.10.1 Data and Safety Monitoring Board (DSMB)......62

COPY

14.10.2 Reporting and Management 62
14.10.3 Confidentiality 63
14.11 Certificate of Confidentiality 63
14.12 Adverse Events (AEs) and Serious Adverse Events (SAEs) 64
15.0 DATA MANAGEMENT AND PROCEDURES 66
15.1 Design and Development 66
15.1.1 Data Collection 66
15.1.2 Data Acquisition and Entry 66
15.1.3 Site Responsibilities 66
15.1.4 Data Center Responsibilities 66
15.1.5 Data Editing 67
15.1.6 Data Transfer/Lock 67
15.2 Data Training 67
15.3 Data Quality Assurance 67
16.0 STUDY TIMELINE 68
17.0 PUBLICATIONS AND OTHER RIGHTS 69
18.0 SIGNATURES 70
19.0 APPENDIX: BAYESIAN ANALYSIS 71
19.1 Figure B5: Bayes power; Alternative Cases 72
19.2 Figure B6: Bayes Credibility Intervals for p1, p2; Alternative Cases 72
19.3 Figure B7: Bayes Credibility Intervals for p1-p2; Alternative Cases 73
19.4 Figure B8: Bayes Test Size; Null Cases 74
19.5 Figure B9: Bayes Credibility Intervals for P1, P2; Null Cases 74
19.6 Figure B10: Bayes Credibility Intervals P1-P2; Null Cases 75
20.0 REFERENCES 76

COPY

1.0 LIST OF ABBREVIATIONS

Abbreviation	Definition
**BNI**	Brief Negotiation Interview
**BUP**	Buprenorphine or buprenorphine/naloxone
**CCC**	Clinical Coordinating Center
**CCTN**	Center for the Clinical Trials Network
**CoC**	Certificate of Confidentiality
**CRF**	Case Report Form
**CTN**	Clinical Trials Network
**DHHS**	Department of Health and Human Service
**DSC**	Data and Statistics Center
**DSMB**	Data Safety Monitoring Board
**DSM-5**	Diagnostic and Statistical Manual of Mental Disorders, Fifth Edition
**eCRF**	Electronic Case Report Form
**eSVR**	Electronic Site Visit Report
**ED**	Emergency Department
**EDC**	Electronic Data Capture
**EDR**	ED Visit Review
**EHR**	Electronic Health Record
**ERC**	Ethics Review Committee
**GCP**	Good Clinical Practice
**HIPAA**	Health Insurance Portability and Accountability Act
**HSP**	Human Subject Protection
**IF**	Implementation Facilitation
**IRB**	Institutional Review Board
**IV**	Intravenous
**LI**	Lead Investigator
**LN**	Lead Node
**MAT**	Medication Assisted Treatment
**ME**	Medical Examiner
**MI**	Motivational Interviewing
**MM**	Medical Monitoring
**NA**	Narcotic Anonymous
**NIDA**	National Institute on Drug Abuse

COPY

Abbreviation	Definition
**NMUPO**	Non-Medical Use of Prescription Opioids
**ORCA**	Organizational Readiness to Change Assessment
**OTP**	Opioid Treatment Program
**OUD**	Opioid Use Disorder
**PARiHS**	Promoting Action on Research Implementation in Health Services
**PC**	Primary Care
**PI**	Principal Investigator
**PT**	Patient
**PPT**	Participant/study participant
**QA**	Quality Assurance
**RA**	Research Associate
**RTS**	Regulatory Tracking System
**SAE**	Serious Adverse Event
**SAMHSA**	Substance Abuse and Mental Health Services Administration
**SBIRT**	Screening, Brief Intervention, Referral and Treatment
**SD**	Standard Deviation
**TLFB**	Time-Line Follow-Back
**WIRB**	Western Institutional Review Board

COPY

2.0 STUDY SYNOPSIS

AIMS

The study will evaluate the impact of (1) Implementation Facilitation (IF) on rates of provision of Emergency Department (ED)-initiated buprenorphine/naloxone (BUP) treatment with referral for ongoing medication-assisted treatment (MAT) and the (2) effectiveness of IF on patient engagement in formal addiction treatment at 30 days.

DESIGN

The proposed project uses a Hybrid Type 3 Effectiveness-Implementation framework and a modified stepped wedge design. In a Hybrid Type 3 Effectiveness-Implementation study the primary research question is the implementation strategy's impact on uptake. In addition, the Hybrid Type 3 design allows one to assess the implementation strategy's impact on related effectiveness outcomes. The study will be conducted at four EDs with a high prevalence of patients with untreated opioid use disorder (OUD), an existing research infrastructure and a potential network of community opioid treatment providers and programs.

POPULATION

The study populations will include:

1) ED providers and staff involved in the treatment of patients with OUD;

2) Community opioid treatment providers and program staff involved in providing care for patients with OUD referred from the ED;

3) Approximately 960 ED patients with moderate to severe OUD.

ELIGIBILITY for Participating in Implementation Facilitation

ED staff: All staff involved in the process of ED initiation of BUP and/or OUD referral.

Community opioid treatment providers and program staff: Providers and staff involved in the provision of office-based BUP and at opioid treatment programs (OTPs).

ED patients: (Criteria described below)

PATIENT INCLUSION/EXCLUSION CRITERIA

Patients 18 years or older who are treated in the ED during study screening hours with DSM-5 criteria for moderate to severe OUD and provide an opioid positive urine sample will be eligible to participate. Note: Patients who test positive for fentanyl *only* are not eligible. Patients will be excluded if they have a medical or psychiatric condition requiring hospitalization at the index ED visit, acutely suicidal or severely cognitively impaired precluding informed consent, presents from an extended care facility, requires continued prescription opioids for a pain condition, be a prisoner or in police custody at time of index ED visit, currently (during the past 30 days) enrolled in formal addiction treatment including by court order, inability to provide 2 contact numbers, unwilling to follow study procedures, prior enrollment in the current study or non-English speaking.

METHODS

A Hybrid Type 3 Effectiveness-Implementation design will be used to evaluate the impact of an IF strategy (consisting of a bundle of implementation activities) on the provision of ED-initiated BUP with referral for ongoing MAT to ED patients with moderate or severe OUD (implementation outcome) and patient engagement in treatment at 30 days (effectiveness outcome) compared with a baseline evaluation period. The baseline evaluation period occurs after a standard educational dissemination activity using a “grand rounds” format. A modified stepped wedge design[1-4] will be used to randomize the order of the four EDs receipt of IF. IF is a multi-component intervention that assesses key stakeholder's need to provide a tailored implementation strategy using an external facilitator, local champions, provider education, academic detailing, stakeholder engagement, performance monitoring and feedback, formative evaluations, a learning collaborative and program marketing to implement ED-initiated BUP with referral for ongoing MAT.[1,5] The IF strategies are iterative and continue into the IF evaluation period.

2.1 Primary Implementation and Effectiveness Aims

In a Hybrid Type 3 Effectiveness-Implementation study the primary research question tests the implementation strategy’s impact on uptake. In addition, the Hybrid Type 3 design allows one to assess the implementation strategy’s impact on related effectiveness outcomes. In the current study the primary research question is the impact of IF on the provision of ED-initiated BUP with referral for ongoing MAT. The effectiveness outcome, in this case patient engagement in formal addiction treatment on the 30th day post enrollment, is also assessed but is not considered co-primary.

IMPLEMENTATION

To compare the baseline evaluation and IF evaluation periods on rates of provision of ED-initiated BUP with referral for ongoing MAT. This is the main outcome of the study for the purposes of analysis. (Section 12.11)

Hypothesis: There will be higher rates of provision of ED-initiated BUP with referral for ongoing MAT in the IF evaluation period compared with the baseline evaluation period

EFFECTIVENESS

To compare the baseline evaluation and IF evaluation periods on rates of patient engagement in formal addiction treatment on the 30th day post enrollment.

Hypothesis: There will be a higher proportion of patients in the IF evaluation period engaged in formal addiction treatment on the 30th day post study enrollment than the baseline evaluation period.

The rates of engagement in formal addiction treatment on the 30th day post enrollment during the baseline and IF evaluation periods will be compared within each site and for the entire study cohort. Engagement in formal addiction treatment will be defined as enrollment and receiving formal addiction treatment on the 30th day post enrollment, assessed by direct contact with the facility and/or treating clinician. Formal addiction treatment will be those treatments consistent with the American Society of Addiction Medicine’s levels of care (1-4), and will include a range of clinical settings including office-based providers of BUP or naltrexone, OTPs, intensive outpatient, inpatient, or residential treatments. Patients do not need to be receiving MAT on the 30th day post enrollment to be considered engaged in formal addiction treatment. Participation in a self-help program such as Narcotics Anonymous (NA) alone will not be considered as engagement in formal addiction treatment.

3.0 INTRODUCTION

3.1 Background and Significance

Untreated opioid use disorder (OUD) remains a major public health problem in the United States despite the availability of effective medication assisted treatments (MATs).[6] Patients with untreated OUD frequently receive care in EDs for treatment of acute and comorbid conditions, opioid overdose and withdrawal, and as a conduit to addiction treatment. EDs typically provide referrals to community services, including opioid treatment programs (OTPs), for OUD treatment rather than directly initiate treatment for this chronic and relapsing condition with medications such as buprenorphine (BUP). We recently demonstrated that ED-initiated BUP with referral for ongoing MAT was superior to such referrals, resulting in nearly twice the percentage of patients who were engaged in formal addiction treatment on the 30th day post enrollment and less illicit opioid use.[7] We propose to use an Implementation Facilitation strategy to accelerate dissemination and promote sustainable implementation of ED-initiated BUP with referral for ongoing MAT. We also propose to build upon prior efficacy work and examine effectiveness of the approach to improve engagement in formal addiction treatment on the 30th day post enrollment. The outstanding unmet need addressed by this project is an urgent public health priority to expand access to MAT for patients with untreated OUD by increasing the use of ED-initiated BUP with referral for ongoing MAT. We hypothesize that this will increase ED provider provision of BUP, patient engagement in formal addiction treatment and reduce illicit opioid use. Implementation Facilitation,[5] an evidence based and reproducible strategy for changing healthcare, holds exceptional promise as a way to increase ED-initiated BUP with referral for ongoing MAT.

3.2 Innovation

We seek to expand access to MAT through the use of the ED, a setting with a high prevalence of untreated OUD traditionally only used as a site for referral rather than initiation of care for this chronic disease. We use a novel strategy from Implementation Science, Implementation Facilitation (IF), to address site-specific needs and support the adoption of ED-initiated BUP with referral for ongoing MAT.[8] To rigorously evaluate the effectiveness of the IF approach, we will conduct a modified stepped wedge design, in which EDs are randomized to the calendar time of receiving the IF and all sites are evaluated repeatedly following a standard dissemination intervention (education only) component and following the IF component. The proposed study will evaluate both the implementation process and its subsequent effectiveness. Additionally, this research will produce training protocols and materials for ED providers to learn and initiate BUP treatment, improved patient screening/evaluation and engagement algorithms, including innovative use of electronic health record screening and decision support tools and tablet-based software programs to facilitate ED diagnosis, evaluation and treatment provision. Upon successful completion of the proposed study, the study sites will have improved capabilities to initiate ED BUP treatment, and improved tools to engage in further training and dissemination efforts regionally and nationally.

3.3 Public Health Impact

EDs are often the source of care for patients with OUD including overdose.[6] ED-based care can impact short-term (e.g., 30 day) engagement in care. Successful completion of this research will provide much needed models and resources to support implementation of ED-initiated BUP and promote engagement in addiction treatment and help to establish EDs as a standard site for initiation of MAT for OUD, paralleling models of treatment for other chronic diseases such as diabetes, asthma and hypertension, thus expanding access to MAT.

3.4 Sustainability

We have opted to use an implementation science framework and IF to specifically address the issue of sustainability. To the extent possible, we will use the IF process to identify and implement real-world and long-term solutions to BUP initiation in the ED with continuing care in the community. This will result in processes and procedures that will remain after the research has been completed and that can be transferred to other settings not involved in the study. If successful the IF strategy would provide a new, reproducible paradigm for clinical experts and policy makers to help expand access to MAT. The study will generate processes, procedures, and deliverables for dissemination to EDs, office-based physicians and OTPs nationally. Several of the CTN nodes and their corresponding EDs are ideally suited for an evaluation of ED-initiated BUP with referral for ongoing MAT. This intervention has demonstrated efficacy in a single site and is ideal for simultaneous evaluation of implementation and effectiveness in multiple sites. We will build on our extensive experience and the current national and state policy momentum for increasing access to MAT of OUD. The CTN has long been a venue to evaluate the provision of treatment services in clinical settings. This protocol provides a unique opportunity to expand access to effective treatment for OUD via ED settings nationally. We will share our products and lessons learned with other CTN nodes, and at Emergency Medicine, Addiction Medicine, Primary Care and community-based treatment provider professional societies and meetings.

4.0 AIMS, OBJECTIVES & HYPOTHESES

4.1 Overview

We have previously demonstrated that ED-initiated BUP with referral for ongoing BUP treatment is superior to referral alone for engaging and retaining patients in formal addiction treatment at 30 days.[7] Thus, ED-initiated BUP offers an innovative strategy to expand urgently needed access to MAT for patients with moderate/severe OUD.[6] Research is needed to develop strategies to efficiently disseminate and implement ED-initiated BUP with referral for ongoing MAT in real-world ED settings. IF is an evidenced-based strategy that provides a bundle of activities (Table 1) to increase the skill set of ED providers and can improve the adoption of effective treatments.[8] We propose an Effectiveness-Implementation Hybrid Type 3 study using a modified stepped wedge design.[1-4,9,10] Four sites will receive the same sequence of interventions: the baseline evaluation period after the initial education component, the IF phase, and continuation of facilitation into the IF evaluation period. Study participants include ED patients with OUD as well as ED and community providers. The timing of initiation of the study activities at each site will be randomly offset by 3 month increments to accommodate logistical constraints of simultaneous implementation at all sites.

4.2 Primary Aims

To compare the baseline evaluation and IF evaluation periods on:

IMPLEMENTATION:

Rates of provision of ED-initiated BUP with referral for ongoing MAT.

Hypothesis: There will be higher rates of provision of ED-initiated BUP with referral for ongoing MAT in the IF evaluation period compared with the baseline evaluation period.

EFFECTIVENESS:

Rates of patient engagement in formal addiction treatment on the 30th day post enrollment

Hypothesis: There will be a higher proportion of patients in the IF evaluation period engaged in formal addiction treatment on the 30th day post enrollment than in the baseline evaluation period.

4.3 Secondary Aims and Hypothesis

4.3.1 Implementation Aims

To compare the baseline evaluation and IF evaluation periods on:

1. Provider fidelity to a critical action checklist relating to the provision of ED-initiated BUP with referral for ongoing MAT

2. Rates of enrolled patients receiving an appointment for opioid treatment provider/program upon ED discharge

3. Number of ED providers receiving DATA 2000 training

4. Number of clinicians providing ED-initiated BUP with referral for ongoing MAT

5. ED provider readiness and preparedness ruler scores to initiate BUP and provide referral for ongoing MAT

6. ED Organizational Readiness to Change Assessment (ORCA) scores[11] relating to ED-initiated BUP with referral for ongoing MAT

7. Community opioid treatment provider/program readiness and preparedness ruler scores to continue MAT for patients with OUD who have received ED-initiated BUP

8. Community opioid treatment provider/program Organizational Readiness to Change Assessment (ORCA) scores relating to receiving patients with OUD who have received ED-initiated BUP

4.3.2 Implementation Hypotheses

1. There will be higher fidelity to the critical action checklist relating to the provision of ED-initiated BUP with referral for ongoing MAT in the IF evaluation period compared with the baseline evaluation period

2. A greater rate of patients in the IF evaluation period will receive an appointment with an opioid provider/program upon ED discharge compared with the baseline evaluation period

3. A greater number of providers will undergo DATA 2000 training in the IF evaluation period compared with the baseline evaluation period

4. A greater number of clinicians will provide ED-initiated BUP with referral for ongoing MAT in the IF evaluation period compared with the baseline evaluation period

5. ED provider readiness and preparedness ruler scores will reflect a greater readiness and preparedness for provision of BUP and referral for ongoing MAT in the IF evaluation period compared with the baseline evaluation period

6. ED ORCA scores will reflect a greater organizational readiness to change with respect to provision of ED-initiated BUP in the IF evaluation period compared with the baseline evaluation period

7. Community opioid treatment provider/program provider readiness and preparedness ruler scores to continue MAT for patients with OUD who have received ED-initiated BUP will be greater in the IF evaluation period compared with the baseline evaluation period

8. Community opioid treatment provider/program ORCA scores relating to receiving patients with OUD who have received ED-initiated BUP will be greater in the IF evaluation period compared with the baseline evaluation period.

4.3.3 Effectiveness Aims (at 30-day follow-up assessment)

To compare the baseline evaluation and IF evaluation periods on:

1. Self-reported days of illicit opioid use (past 7 days) as measured by Time-Line Follow-Back (TLFB) methods at 30 days
2. Overdose events (past 30-days) captured by participant self-report, state medical examiner records, National Death Index and review of medical records
3. HIV risk taking behaviors (past 30 days) as measured by HIV Risk Taking Behavior Scale
4. Healthcare service utilization (past 30 days)
5. Rates of illicit opioid negative urines at 30 days

4.3.4 Effectiveness Hypotheses (at 30-day follow-up assessment)

Patients in the IF evaluation period compared with baseline evaluation period will:

1. Report fewer days with illicit opioid use in the past 7 days
2. Experience fewer overdose events (past 30 days)
3. Report fewer HIV risk taking behaviors (past 30 days)
4. Utilize fewer inpatient OUD services (past 30 days)
5. Have greater rates of illicit opioid negative urine tests at 30 days

4.4 Exploratory Aims

4.4.1 Study Patient, Emergency Department and Provider Characteristics

We will evaluate a limited set of study patient, ED and ED provider characteristics for their potential effect on the primary outcomes.

IMPLEMENTATION

ED characteristics such as size, location, existing addiction treatment services, and follow-up resources as well as the range and number of addiction treatment services in the catchment area of the ED will be evaluated. Provider characteristics such as age, gender, years and level of training will be evaluated.

EFFECTIVENESS

Study patient characteristics such as type of opioid used (prescription vs. heroin) mode of use (IV/PO/intranasal), frequency of use, demographics, pain intensity and interference, and reason for ED presentation such as overdose, seeking treatment or visit related to substance use, comorbid substance use and referral to office-based versus opioid treatment program will be evaluated. The overall effectiveness of ED-initiated BUP compared with other interventions during both baseline evaluation and IF evaluation periods will be evaluated.

4.4.2 Cost effectiveness

We will perform an incremental cost effectiveness analyses (CEA) and calculate the incremental cost per unit increase in engagement in formal addiction treatment comparing patients in the IF evaluation period with those in the baseline evaluation period.

We will perform incremental cost effectiveness analyses (CEA) to evaluate if effectiveness benefits are worth the additional costs.

COPY

5.0 STUDY DESIGN

5.1 Figure 1: Overview: Study Activities

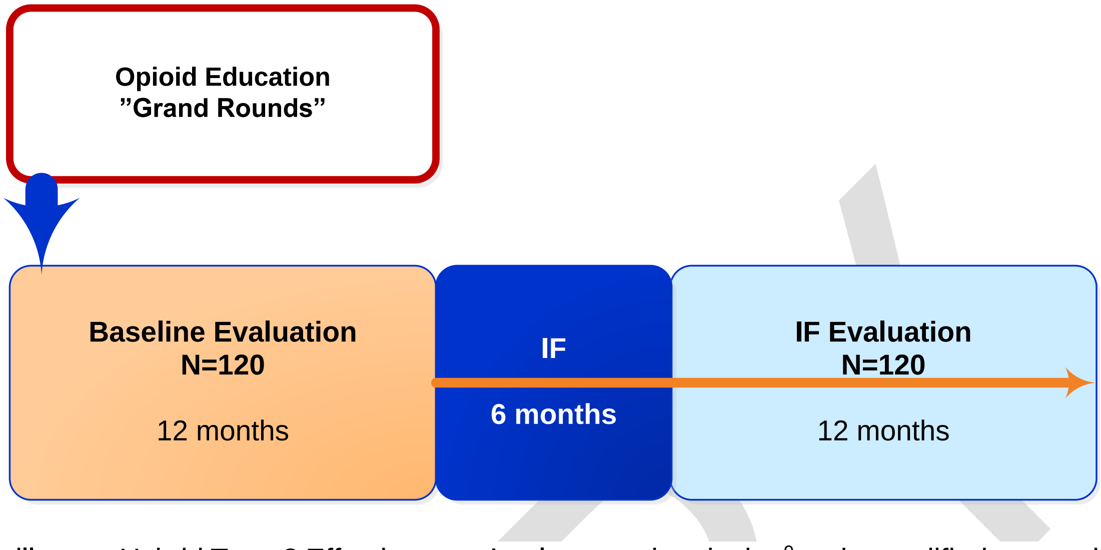

We will use a Hybrid Type 3 Effectiveness-Implementation design[9] and a modified stepped wedge design[1-4,10] to evaluate the effect of the IF on ED-initiated BUP with referral for ongoing MAT and its effectiveness. A Hybrid Type 3 design is recommended when there is strong face validity and strong initial evidence for the clinical and implementation interventions that would support generalizability; there is strong "implementation momentum" for adoption of the clinical intervention and evidence that the intervention being tested is feasible and supportable. In light of our prior work and the emphasis nationally on addressing the opioid epidemic, a Hybrid Type 3 design is warranted. The advantages of this design include a balanced provision of resources and support across all study sites, enrollment of comparable cohorts of participants during baseline evaluation and IF evaluation phases across all sites, and the completion of the proposed study in a short period of time (48 months). This overall short study time will minimize the potential impact of population related time trends and other time related factors such as changes in healthcare practice and policy. The randomized order of the timing of the IF at each site, offset by 3 month increments, is used to accommodate logistical constraints of a simultaneous implementation at all sites. The stepped wedge design is increasingly used to promote the implementation of evidence-based practice and is appropriate for evaluating interventions when there is evidence that the intervention is likely to be beneficial and when practical considerations require a sequential deployment of an intervention.[4,10] At each of the four study sites, there will be a 12-month baseline evaluation period after the standard dissemination activity via a "grand rounds" education session, followed by a 6-month IF phase, followed by a 12-month IF evaluation period. A 6-month IF phase is required to allow adequate time to conduct the multiple components of the IF (see Table 1). An evaluation period of 12 months is necessary to enroll enough patients for adequate sample size. The number of patients should be similar during the baseline evaluation and the IF evaluation periods. The strategies included in the IF are iterative and continue throughout the IF and IF evaluation periods. The proportion of eligible study participants in each time period who are engaged in formal addiction treatment on the 30th day post enrollment will be assessed within each site and for the entire study cohort.

Implementation Science and the Promoting Action on Research Implementation in Health Services (PARiHS) framework can guide efforts to promote the uptake of ED-initiated BUP with referral for ongoing MAT

Implementation Science, defined by the National Institute of Health as **the study of methods to promote the integration of research findings and evidence into healthcare policy and practice,**[12] provides an organized approach and tools for addressing the gap between the need and provision of ED-initiated BUP and ongoing MAT. We will use a proven Implementation Science strategy, Implementation Facilitation (sometimes referred to as Practice Facilitation) which provides a bundle of interlinked implementation activities. The goal will be to support ED providers and to promote uptake of ED-initiated BUP and referral for ongoing MAT among eligible patients. We will initially conduct a formative assessment of site-specific organizational, provider, and patient factors potentially impacting uptake of provision of ED-initiated BUP to refine the IF.

The Promoting Action on Research Implementation in Health Services (PARiHS) framework was first published in 1998 and refined in 2008.[13-16] The recent revisited framework identifies four elements for determining successful implementation of an evidence-based practice into clinical care:

1. Nature of the **innovation**;
2. **Recipients** of the facilitation, including people affected by and who influence implementation of the innovation;
3. Qualities of the local and outer (i.e., social, policy, regulatory and political infrastructures) **context** in which the evidence is being introduced and enacted upon; and
4. **Facilitation** (i.e., the implementation intervention), the active process of promoting implementation by assessing and responding to the recipients and associated context.[4,13-15] Johnson and colleagues expanded the PARiHS domains to allow for a more specific application[17] and has informed the development of a measure of organizational readiness to change, the Organizational Readiness to Change Assessment (ORCA),[11] that we use, after tailoring the items to address issues related to ED-initiated BUP.

5.2 Implementation Facilitation Leads to Practice Change

IF is defined as a "process of 'helping individuals and teams to understand what they need to change and how they need to change it in order to apply evidence to practice."[18] IF is an effective intervention[5] that includes a "deliberate process of interactive problem solving and support that occurs in the context of a recognized need for improvement and supportive interpersonal relationship."[17] A recent systematic review and meta-analysis found that IF has a positive impact on guideline adoption in primary care.[5] A central aspect of IF includes the active role of the facilitator(s) working in partnership with relevant stakeholders, and other implementation intervention components.[17,18] Importantly, IF involves a "formative evaluation" to identify the specific and dynamic needs of stakeholders and the context (i.e., ED) for implementation of evidence-based practices.[18] This "diagnostic" formative evaluation informs the initial tailoring and refinement of the IF, which includes the "bundle" of services[18] (Table 1) tailored to meet site-specific needs. The formative evaluation process is iterative and occurs during the 6 month IF period and continues throughout the IF evaluation period.[19] Tailored implementation strategies have been found to be more effective than non-tailored interventions in changing practice[20] and have been applied to changing primary care and mental health treatment delivery.[8,21] IF has been used by large healthcare organizations including the Veterans Health Administration and is endorsed by the Agency for Health Care Research and Quality as a way to assist practices in becoming Primary Care Medical Homes.[22]

5.2.1 **Table 1**: Components of Implementation Facilitation

Component	Description
**External Facilitator**	Outside content expert who assists site
**Local Champion**	Local site stakeholder who promotes change
**Provider Education and Academic Detailing**	Provision of unbiased peer education
**Stakeholder Engagement**	Aligning goals of implementation and those impacted
**Tailor Program to Site**	Addressing site-specific needs based on formative assessment
**Performance Monitoring and Feedback**	Assess implementation of treatment efforts and inform site of results
**Formative Evaluation**	Quantitative and qualitative determination of impact
**Establish a Learning Collaborative**	Shared learning opportunities tailored to stakeholders
**Program Marketing**	Efforts designed to increase attention to availability of ED-initiated BUP

5.3 Duration of Study

Each ED site will need 2 months of startup time to hire the research associates and prepare for study procedures, and will enroll study patients for 12 months during the baseline evaluation period. Study patients will then be evaluated 30 days post enrollment. The initial IF phase will last 6 months and will be followed by 12 months of study patient enrollment in the IF evaluation period and enrolled study patients will be evaluated 30 days post study enrollment. We anticipate completing study patient enrollment and follow up by 42 months. We proposed an additional 6 months for analysis of the data and NIDA CTN presentation (see study timeline section 5.3.1).

5.3.1 Study Timeline Estimated Dates

<table>
<tr><th></th><th colspan="12">Study Year 1</th><th colspan="12">Study Year 2</th><th colspan="12">Study Year 3</th><th colspan="12">Study Year 4</th></tr>
<tr><td>M</td><td>1</td><td>2</td><td>3</td><td>4</td><td>5</td><td>6</td><td>7</td><td>8</td><td>9</td><td>10</td><td>11</td><td>12</td><td>13</td><td>14</td><td>15</td><td>16</td><td>17</td><td>18</td><td>19</td><td>20</td><td>21</td><td>22</td><td>23</td><td>24</td><td>25</td><td>26</td><td>27</td><td>28</td><td>29</td><td>30</td><td>31</td><td>32</td><td>33</td><td>34</td><td>35</td><td>36</td><td>37</td><td>38</td><td>39</td><td>40</td><td>41</td><td>42</td><td>43</td><td>44</td><td>45</td><td>46</td><td>47</td><td>48</td></tr>
<tr><td>DATE</td><td>2/17</td><td>3/17</td><td>4/17</td><td>5/17</td><td>6/17</td><td>7/17</td><td>8/17</td><td>9/17</td><td>10/17</td><td>11/17</td><td>12/17</td><td>1/18</td><td>2/18</td><td>3/18</td><td>4/18</td><td>5/18</td><td>6/18</td><td>7/18</td><td>8/18</td><td>9/18</td><td>10/18</td><td>11/18</td><td>12/18</td><td>1/19</td><td>2/19</td><td>3/19</td><td>4/19</td><td>5/19</td><td>6/19</td><td>7/19</td><td>8/19</td><td>9/19</td><td>10/19</td><td>11/19</td><td>12/19</td><td>1/20</td><td>2/20</td><td>3/20</td><td>4/20</td><td>5/20</td><td>6/20</td><td>7/20</td><td>8/20</td><td>9/20</td><td>10/20</td><td>11/20</td><td>12/20</td><td>1/21</td></tr>
<tr><td>ED1</td><td colspan="2">Start</td><td colspan="12">Baseline evaluation period</td><td colspan="6">IF</td><td colspan="12">IF evaluation period</td><td>F</td><td colspan="15"></td></tr>
<tr><td>ED2</td><td colspan="2"></td><td colspan="2">Start</td><td colspan="12">Baseline evaluation period</td><td colspan="6">IF</td><td colspan="12">IF evaluation period</td><td>F</td><td colspan="13"></td></tr>
<tr><td>ED3</td><td colspan="4"></td><td colspan="2">Start</td><td colspan="12">Baseline evaluation period</td><td colspan="6">IF</td><td colspan="12">IF evaluation period</td><td>F</td><td colspan="11"></td></tr>
<tr><td>ED4</td><td colspan="6"></td><td colspan="2">Start</td><td colspan="12">Baseline evaluation period</td><td colspan="6">IF</td><td colspan="12">IF evaluation period</td><td>F</td><td colspan="9"></td></tr>
<tr><td>All</td><td colspan="42"></td><td colspan="6">Analysis</td></tr>
<tr><td colspan="49">KEY: IF= IMPLEMENTATION FACILITATION Phase, F=FOLLOW-UP ASSESSMENTS, M=MONTH</td></tr>
</table>

COPY

6.0 STUDY SETTINGS

6.1 Number of ED Sites

Four EDs will participate as enrolling sites for the study. This allows for conduct of the study within the 48-month time period giving ample time for patient enrollment into the study during the baseline evaluation and IF evaluation periods at each site assuming a high prevalence of untreated moderate/severe OUD. It also allows for adequate time to initiate and continue a meaningful IF at each site.

6.2 ED Characteristics

Participating EDs should have the following characteristics and respond to the Data Call regarding:

- Large prevalence of patients with untreated OUD so that the target of 10-12 patients enrolled into the study per month can be met. Sites must provide number of ICD 9/10 codes for the past year related to overdose and opioid dependence, abuse and unspecified use/opioid use disorders
- An electronic health record that can be queried daily to weekly
- Wireless internet access as information entered on tablets will be uploaded to a secure study server
- Prior clinical research experience (report funded projects, enrollment, retention, etc.)
- An Emergency Physician with experience as Principal Investigator (PI) and with time to devote to the project
- No current routine use of ED-initiated BUP
- Ability to have BUP on their formulary and available to the ED
- Ability to present a plan for patient flow and space utilization
- Have or are able to hire appropriate staff to conduct the study
- Have sufficient referral network for patients needing MAT that could potentially accommodate referrals in 96 hours
- Be in a state that allows for MAT through its Medicaid program
- Have accessible pharmacies to fill BUP prescriptions
- Have an active state prescription monitoring program

COPY

6.3 Rationale for ED Criteria

The volume of patients seen in the ED must be adequate to ensure a steady stream of eligible patients for recruitment. A diverse, representative sample of patients and participants is desirable to enhance the external validity of the study. Geographical diversity of ED settings are desirable and research experience and successful participation in prior studies is essential as it provides evidence of the feasibility of study implementation. Patient flow and space utilization are challenging in the ED setting. The Research Associates (RAs) will preferably have experience working in ED settings, and therefore can be quickly integrated into the ED staffing team.

6.4 ED Providers

We will include all ED providers credentialed to practice in the site ED that are capable of prescribing BUP. This includes physicians, nurse practitioners (NPs) and physician assistants (PAs).[23,24]

6.5 Community Opioid Treatment Providers and Programs

Each ED may use multiple locations to refer patients for ongoing MAT. This would include opioid treatment programs and office-based providers.

Participating community opioid treatment providers and programs should have the following characteristics:

- At least one office-based provider of BUP not currently at their limit according to DATA 2000 provisions, and one OTP without active waiting lists.
- Programs and/or providers with the ability to accept patients with a variety of insurance plans (including Medicaid) within 96 hours of ED-initiated BUP.
- Treatment providers and/or programs located within the general vicinity of where ED patients reside.

6.6 Rationale for Community Opioid Treatment Providers and Program Criteria

These criteria ensure that there will be at least one of each type of provider in the community to whom patients who receive ED-initiated BUP can be referred. We will work to engage all office-based providers of BUP and OTPs in each ED's catchment area in receipt of patients with ED-initiated BUP.

COPY

7.0 STUDY POPULATION

Patients with OUD enrolled in effectiveness evaluation

7.1 Inclusion Criteria

All patients enrolled into the study must:

1. Be 18 years or older
2. Treated in the ED during study screening hours
3. Meet DSM-5 diagnostic criteria for moderate to severe opioid use disorder
4. Have a urine toxicology test that is positive for opioids (opiates, oxycodone, buprenorphine, or methadone.) For patients with acute pain conditions requiring opioid administration, urines will need to be obtained prior to ED opioid medication administration.

7.2 Exclusion Criteria

All patients enrolled into the study must not:

1. Have a medical or psychiatric condition that requires hospitalization at the index ED visit
2. Be actively suicidal or severely cognitively impaired precluding informed consent
3. Present from extended care facility (e.g., skilled nursing facility)
4. Require continued prescription opioids for a pain condition
5. Be a prisoner or in police custody at the time of index ED visit
6. Currently have (past 30 days) enrolled in formal addiction treatment, including by court order
7. Inability to provide reliable locator information including 2 contact numbers
8. Be unwilling to follow study procedures (e.g., unwilling to provide permission to contact referral provider/program or return for 30-day assessment)
9. Have prior enrollment in the current study

   Note: A patient may **NOT** enroll in both the baseline evaluation and IF evaluation periods.

10. Not able to speak English sufficiently to understand the study procedures and provide written informed consent to participate in the study

COPY

7.3 Feasibility

An annual ED visit of 60,000 or greater, should provide an adequate volume of participants available for screening and enrollment along with the requisite ≧ 300 unique ICD codes related to opioid poisoning and dependence as reported in the site selection data call document. To ensure that recruitment and enrollment is done consistently and accurately with sequential patients, avoiding convenience sampling, each site is expected to have the equivalent of 3.0 full time Research Assistants (RAs) that will provide cross coverage over their assigned shifts. The number of patients screened at each site will vary based on the number of overall ED visits at each site. Each site will need to document the potential to enroll 10-12 patients into the study per month. This will allow the study to enroll approximately 960 patients overall into the study.

COPY

8.0 OUTCOME MEASURES

8.1 Primary Implementation Outcome Measure

Aim: To compare the baseline evaluation and IF evaluation periods on rates of provision of ED-initiated BUP with referral for ongoing MAT.

Hypothesis**:** There will be higher rates of provision of ED-initiated BUP with referral for ongoing MAT in the IF evaluation period compared with the baseline evaluation period.

The rates of provision of ED-initiated BUP with referral for ongoing MAT will be based on:

1. Proportion of enrolled patients who receive ED-initiated BUP with referral for ongoing MAT or
2. Computed from ED data on the numbers of providers and their patients who received ED-initiated BUP with referral for ongoing MAT

   As estimating a very low frequency outcome by sampling is not reliable, computation will be used if #1 results in zeros or small ratios. This entails reviewing existing ED records and counting the numbers of ED providers assigned to patients with OUD, and the number of patients who received ED-initiated BUP with referral for ongoing MAT during the study period. (See analysis section)

8.2 Primary Effectiveness Outcome Measure

Aim: To compare the baseline evaluation and IF evaluation periods on rates of patient engagement in formal addiction treatment on the 30th day post study enrollment.

Hypothesis**:** There will be a higher proportion of patients in the IF evaluation period engaged in formal addiction treatment on the 30th day post study enrollment than in the baseline evaluation period.

The rates of engagement in formal addiction treatment at the 30th day post study enrollment during the baseline and IF evaluation periods will be compared within each site and for the entire study cohort. Engagement in formal addiction treatment will be defined as enrollment and receiving formal addiction treatment on the 30th day post study enrollment, assessed by direct contact with the facility and/or treating clinician. Formal addiction treatment will be those treatments consistent with the American Society of Addiction Medicine’s levels of care (1-4), and will include a range of clinical settings including office-based providers of BUP or naltrexone, OTPs, intensive outpatient, inpatient, or residential treatments. Patients do not need to be receiving MAT on the 30th day post enrollment to be considered engaged in formal addiction treatment. Participation in a self-help program such as NA alone will not be considered as engagement in formal addiction treatment. Additional analyses evaluating the effects of patient characteristics will also be conducted.

8.3 Secondary Implementation Outcome Measures

To compare the baseline evaluation and IF evaluation periods on:

1. Provider fidelity to a critical action checklist relating to the provision of ED-initiated BUP with referral for ongoing MAT

2. Rates of enrolled patients receiving an appointment for opioid treatment provider/program upon ED discharge

3. Number of ED providers receiving DATA 2000 training

4. Number of clinicians providing ED-initiated BUP with referral for ongoing MAT

5. ED provider readiness and preparedness ruler scores to initiate BUP and provide referral for ongoing MAT

6. ED Organizational Readiness to Change Assessment (ORCA) scores[11] relating to ED-initiated BUP with referral for ongoing MAT

7. Community opioid treatment provider/program readiness and preparedness ruler scores to continue MAT for patients with OUD who have received ED-initiated BUP

8. Community opioid treatment provider/program Organizational Readiness to Change Assessment (ORCA) scores relating to receiving patients with OUD who have received ED-initiated BUP

8.4 Secondary Effectiveness Outcome Measures

To compare the baseline evaluation and IF evaluation periods on:

1. Self-reported days of illicit opioid use (past 7 days) measured by TLFB methods at 30 days
2. Overdose events (past 30 days) captured by participant self-report, state medical examiner records, National Death Index and review of medical records
3. HIV risk taking behaviors (past 30 days) as measured by HIV Risk Taking Behavior Score
4. Healthcare service utilization (past 30 days) measured by Health Services Utilization Form
5. Rates of illicit opioid negative urines at 30 days

8.5 Exploratory Outcomes

We will evaluate a limited set of study patient, ED and ED provider characteristics for their potential effect on the implementation and effectiveness outcomes.

IMPLEMENTATION

ED characteristics such as size, location, existing addiction treatment services and follow up resources as well as the range and number of addiction treatment services in the catchment area of the ED will be evaluated. Provider characteristics such as age, gender, years and level of training will be evaluated .

EFFECTIVENESS

Patient characteristics such as type of opioid used (prescription vs. heroin) mode of use (IV/PO/intranasal), frequency of use, demographics, pain intensity and interference, and reason for ED presentation such as overdose, seeking treatment or visit related to substance use, comorbid substance use, and referral to office-based versus opioid treatment program treatment will be evaluated. The overall effectiveness of ED-initiated BUP compared with other interventions during both baseline and IF evaluation periods will be evaluated.

COST EFFECTIVENESS

We will perform an incremental cost effectiveness analyses (CEA) and calculate the incremental cost per unit increase in engagement in formal addiction treatment comparing patients in the IF evaluation period with those in the baseline evaluation period.

We will perform incremental cost effectiveness analyses (CEA) to evaluate if effectiveness benefits are worth the additional costs.

COPY

9.0 STUDY PROCEDURES

9.1 Screening and Informed Consent Procedures Baseline and IF Evaluation Periods

9.1.1 Screening

Patients will be recruited throughout the entire evaluation period at each ED site. Research Associates (RAs) assigned to the study will work in shifts to ensure cross coverage to screen all ED patients who are potentially eligible for the study. The RA will use the ED log to identify all patients seen in the ED and will eliminate patients with obvious exclusions such as under police custody. The RA will keep a log of all patients excluded and the reasons for exclusion. Patients will be evaluated by an RA for eligibility.

Patients will be asked for verbal consent to complete a set of screening assessments starting with a screener that includes questions about illicit opioid use in the past 30 days embedded in a general health and substance use screener that also includes questions about safety, tobacco and alcohol use.[25,26] We have successfully used this strategy of embedding questions about opioid use in a general health survey as a method to screen for drug and alcohol use. Embedded questions have also been noted by the World Health Organization to improve the reliability of self-reported behavior. The screener will contain questions regarding heroin/fentanyl and non-medical use of prescription opioids (ED Health Quiz screener).

Potential study patients who report any opioid use in the past month will complete a 7-day TLFB. If opioid use is reported during the past 7 days a brief (10 minute) structured diagnostic interview (DSM-5) to evaluate for the presence of moderate/severe OUD is administered. Potential study patients who do not meet criteria for moderate/severe OUD will not be asked to participate and will be given a handout recommending that they abstain from drug use and a list of local referral options at the discretion of their ED providers. Those who meet criteria for moderate/severe OUD will be informed that they may qualify for a study if they are willing to produce a urine sample If the urine tests are positive for any opioid (*fentanyl only are not eligible*), the patient indicates he/she is able to provide contact information for 2 reliable contacts and the patient meets all eligibility criteria on the Patient Participant Eligibility Summary form, patients will be offered participation and informed consent will be obtained.

9.1.2 Informed Consent Procedures (Patient Participants)

We will use the Western Institutional Review Board (WIRB) as the IRB of record for the study and comply with all necessary requirements at each site. Each site will utilize the IRB-approved compound authorization form that serves as a combined informed consent and HIPAA disclosure form allowing study access to protected health information in the patient’s ED medical record. Because of the time constraints of completing the assessment and intervention in the ED, and because of the relatively low risks associated with the study, the informed consent form will be as brief as possible within the constraints of adequate human subjects’ protections. The compound authorization document will include a description of the following key elements including: the assessment interview and questionnaires; the follow-up interviews; risks and benefits of study procedures; alternatives to participation in the study; confidentiality; payment for participation; a statement that participation is voluntary and that they may freely withdraw participation at any time; and information about whom to contact with questions or in case of emergency. The form will also include assurances of confidentiality (including a Certificate of Confidentiality) and that the decision to participate will in no way influence other aspects of the patient’s treatment.

In addition, potential participants will be instructed concerning the importance of telling the truth and that compensation and continuation in the study has nothing to do with their drug use or test results. Initial screening questions will be asked with verbal consent, however, all eligible study patients will be asked for written consent to participate using the IRB-approved compound authorization form.

9.1.3 Eligibility Confirmation and Enrollment

Once the eligibility is confirmed and written informed consent (compound authorization) is obtained from the study patient, he/she will be considered enrolled in the study. The enrollment procedures will be captured through a centralized process managed by the CTN Data and Statistics Center (DSC). Study patients who do not complete all screening assessments or who are otherwise found to be ineligible for participation in the study will be considered screen failures. Section 10.0 describes all study assessments.

9.2 Participant Withdrawal

At any time, participants may decide that they no longer wish to continue to participate in the study.

9.3 Participant 30-day Follow-up Assessments

Follow-up assessments are expected to be conducted at 30-days post study enrollment in-person by the ED study staff. Assessment of the effectiveness study outcome, enrollment in formal addiction treatment on the 30th day post enrollment will be based on direct contact with the treatment provider/program provided by the patient. Printed appointments for the follow-up will be given to the study patient participant prior to discharge. In addition, the RA will send a reminder in the mail/text/email/or social media (based on study participant preference) about 7 days prior to the scheduled follow-up visit with an additional reminder phone call the day before the scheduled follow-up visit.

9.4 Compensation

Because of the expected difficulty of maintaining high follow-up rates in the study population, adequate compensation for time and inconvenience is critical. Compensation of $50 for completing the enrollment process at the initial ED visit, and $50 for the 30-day post enrollment follow-up visit. Follow up is expected to be conducted in person in the ED or at an offsite research location but may be completed as a telephone visit if a participant is unable to attend visit. A urine sample will not be collected if the visit is completed via telephone.

COPY

10.0 STUDY PATIENT ASSESSMENTS

10.1 Baseline Evaluation and IF Evaluation Periods

The screening and follow-up assessments for this study are brief balancing the value of comprehensive data against feasibility and to minimize assessment reactivity that can obscure treatment effects.[27] The practical issue is that extensive study patient participant level assessments are likely to interfere with the rapid pace of clinical treatment in the ED setting. A cumbersome assessment process is also likely to impede the successful completion of the study through an adverse effect on recruitment, and would not be part of real world clinical practice. Excluding collection of study patient participant characteristics and locator information, the patient baseline data will include a brief instrument assessing health status, healthcare utilization, overdose events, past 7-day alcohol and drug use including opioids using the Time-line Follow Back (TLFB)[28] method, use of other substances, the EuroQol (EQ-5D) and other cost data necessary to compare costs with the costs and benefits of other funded interventions. Table 2 provides the schedule of study assessments. The total expected time burden for the screening assessments is less than 30 minutes. Assessments collected at 30 days post study enrollment will be similar.

COPY

10.1.1 Table 2: Schedule of Study Activities and Assessments (Study Patient) by Study Time Period

Instrument/Activity	Time	Done by	Baseline Evaluation Period (12 Months)			IF (6 Months)	IF Evaluation Period (12 Months)		
			Screening	Enroll-ment	30 Day Follow-up		Screening	Enroll-ment	30 Day Follow-up
			(Index ED Visit 1)		(Visit 2)		(Index ED Visit 1)		(Visit 2)
ED Health Quiz	2’	RA	X				X		
DSM-5	5’	RA	X				X		
Urine Drug Screen	5’	RA	X				X		
Patient Eligibility Summary	2’	RA	X				X		
Written Compound Informed Consent	10’	RA		X				X	
Demographics and Additional Characteristics	1’	RA		X				X	
Locator Information form	2’	RA		X				X	
Other Substance Use	1’	RA		X				X	
Timeline Follow-Back (TLFB)	10’	RA		X	X			X	X
Health Services Utilization (Inpatient and Outpatient)	6’	RA		X	X			X	X
Health status (HRBS/PHQ9/PEG)	3’	RA		X	X			X	X
Overdose	1’	RA		X	X			X	X
EuroQol-5D	2’	RA		X	X			X	X
Crime and Criminal Justice	1’	RA		X	X			X	X
Urine Drug Screen	5’	RA			X				X
Healthcare Visit Logistics	1’	RA			X				X
Engagement in Treatment	5’	RA			X				X
ED Visit Review		RA		X				X	
Critical Action Checklist		RA*		X				X	
ED Visits and Hospitalizations		RA			X				X
Study Completion		RA			X				X
Serious Adverse Event (Death)		RA	As Needed				As Needed		
Protocol Deviations		RA	As Needed				As Needed		
**Total Duration in Minutes for Participant Interaction:**			**14’**	**37’**	**34’**		**14’**	**37’**	**34’**

* Site staff, with PI input as needed.

10.2 Study Patient Participant Assessments

10.2.1 ED Health Quiz

Individuals will meet with an RA to be evaluated for study eligibility. This assessment will be conducted after verbal consent, and before enrollment into the study. It will include questions about illicit opioid use in the past 30 days embedded in a general health and substance use screener that also includes questions about safety, tobacco and alcohol use.

10.2.2 DSM-5

The DSM-5 criteria are assessed during the screening period to determine a current diagnosis of moderate or severe opioid use disorder. This assessment will be completed electronically and will be automatically scored.

10.2.3 Urine Drug Screen (Toxicology Testing)

Urine testing will be performed for the presence of the following drugs: opioids, oxycodone, benzodiazepines, cocaine, methamphetamine, amphetamine, 3.4-methylenedioxy-methamphetamine (MDMA), tetrahydrocannabinol (THC), barbiturate, methadone, buprenorphine and fentanyl. The urine drug screen is collected during the screening phase and at 30-days post study enrollment along with the other assessments. The fentanyl urine drug screen is being used for research purposes only. Urine testing supplies (e.g., dip stick) will be provided to the sites.

10.2.4 Patient Eligibility Summary and Compound Informed Consent and Inclusion/Exclusion

The Patient Eligibility Summary form collects information regarding eligibility during the screening phase, before written informed consent is obtained. After written compound informed consent, individual inclusion and exclusion criteria listed is assessed to confirm documentation of eligibility. Only patients who continue to meet study eligibility criteria will be allowed to continue to the enrollment phase.

10.2.5 Demographics and Additional Characteristics

The demographics forms collect information about demographic characteristics of the study participant, including age, gender, cultural/ethnic group, educational level, marital status, and type of insurance.

10.2.6 Locator Information Form

A locator form is used to obtain information to assist in finding study participants during the baseline evaluation and IF evaluation periods. This form collects contact information including the participant’s current address, email address and phone numbers. In order to facilitate locating participants if direct contact efforts are unsuccessful, addresses and phone numbers of family/friends who may know how to reach the participant are collected. This information will be collected at enrollment and updated at the follow-up visit, or when the participant reports a change in locator information. No information from this form will be used in data analyses.

10.2.7 Other Substance Use

Selected questions from the ASSIST-lite will be used to assess severity of drug and alcohol problems over the past 3 months and will be asked at enrollment only. Questions related to opioid use have been eliminated to avoid duplication.

10.2.8 Timeline Follow-back (TLFB)

The Timeline Follow-back procedure (Sobell & Sobell, 1992[28]; Fals-Stewart, 2000[34]) will be used to elicit the patient participant's self-reported use, quantity, and route of administration of opioids, marijuana, stimulants, benzodiazepines and alcohol. At enrollment, substance use is reported by the patient participant for the 7-day period prior to informed consent.[28] At the 30 day follow-up visit, substance use is reported for the 7 days prior to study day 30.

10.2.9 Health Services Utilization Inpatient and Health Services Utilization Outpatient [29]

A brief, structured interview regarding health care utilization (inpatient and outpatient) will be used, which collects information on the type and amount of services received. This includes ED visits, hospitalizations, primary medical care visits (excluding those for buprenorphine treatment and self-help sources of support (e.g., NA)). It is collected at enrollment and at 30 days post study enrollment.

10.2.10 Health Status

The Health Status form collects information on HIV and Hepatitis C status, HIV risk (CTN HRBS), Pain (PEG), and psychological health (PHQ-9)[31] usual care and reason for ED visit. The HIV Risk-taking Behaviour Scale (HRBS)[32] is a brief, 12-item questionnaire measuring the behavior of people who inject drugs that puts them at risk of either contracting/transmitting HIV. Also embedded within is the Patient Health Questionnaire (PHQ-9)[31] used to assess depressive symptoms. Health status questions are collected at enrollment and at 30 days post study enrollment.

10.2.11 Overdose Events

We will ask participants about past 30-day opioid-related overdose events.[33] Assessment of past 30-day overdose events will be completed at enrollment and at 30 days post study enrollment. In addition, Site PIs will search the National Death Index, and local electronic medical records for overdose events.

10.2.12 EuroQol (EQ-5D)

The EuroQol (EQ-5D)[30] is a structured interview that collects general health information applicable to a wide range of health conditions and treatments, providing a simple descriptive profile and a single index value for health status. The EQ-5D is collected at enrollment and at 30 days post study enrollment.

10.2.13 Crime and Criminal Justice

This form captures data on incarceration, recent crimes, and recent contact with the law and is collected at enrollment and at 30 days post study enrollment.

10.2.14 Healthcare Visit Logistics

The Healthcare Visit Logistics form asks cost data questions at 30 days post study enrollment.

10.2.15 Engagement in Treatment Survey

At 30 days post enrollment, participants will be asked to report OUD treatment received on their 30th day post enrollment target date (Day 30, with the ED enrollment visit being Day 0). Data will be reported on the Engagement in Treatment: Patient Survey. The effectiveness outcome will be confirmed with the addiction treatment provider. Engagement is based on enrollment in treatment on the 30th day post enrollment (Enrollment is considered Day 0). The Engagement in Treatment: Facility survey includes the type of treatment the participant is receiving, i.e., methadone, buprenorphine and/or naltrexone treatment, detoxification, residential or inpatient treatment. Date of admission is recorded as well as the level of treatment received according to ASAM Levels of care, such as Level I: Outpatient Treatment; Level II: Intensive outpatient treatment (including partial hospitalization); Level III: Residential/Inpatient Treatment; Level IV: Medically managed intensive inpatient treatment or Other-specified.

10.2.16 ED Visit Review

The ED Visit Review (EDR) form collects information about study and clinical processes surrounding the index ED visit. It is completed by the Senior RA without patient input, in part by performing a medical record abstraction. EDR data may be independently reviewed for accuracy.

10.2.17 Critical Action Checklist

The critical action checklist provides documentation of best practices for ED-initiated buprenorphine including: urine toxicology and liver function tests obtained; patient participant meets criteria for DSM-5 moderate-severe opioid use disorder; urine positive for opioid; formal assessment of Clinical Opioid Withdrawal Scale (COWS) score; ED-initiated BUP provided; BUP education and induction instructions provided; and referral for ongoing medication assisted treatment. Data is acquired for this form by performing a medical record abstraction by a site staff member that is blinded to the index visit.

10.2.18 ED Visits and Hospitalizations

The ED Visits and Hospitalizations form collects information about the index ED visit and any visits or hospitalizations between the index and follow-up. Data is acquired for this form by performing a medical record abstraction.

10.2.19 Study Completion

The participant's status with regard to study visits will be recorded at the end of the study, providing information on whether the participant completed the follow-up visit at day 30, and providing a location to document withdrawal of consent or other reasons for not completing the follow-up visit.

10.2.20 Adverse Events (AEs) and Serious Adverse Events (SAEs)

All deaths, regardless of cause, will be captured for this study. These events may be identified when possible through using state medical examiner records, National Death Index, and review

COPY

of medical records. Appropriate information surrounding the event will be documented on the AE/SAE forms set. Only death events that occur within the 30 day follow-up period will be recorded on the AE/SAE form set.

10.2.21 Protocol Deviation Form

This form should be entered into the electronic data capture system whenever a protocol deviation occurs. This form will document a description of the deviation, how it occurred, the corrective action taken to resolve the specific deviation, as well as a description of the plan implemented to prevent future occurrences of similar deviations.

COPY

11.0 IMPLEMENTATION FACILITATION (IF) STUDY PROCEDURES

11.1 Standard Dissemination Practice - Education

A major goal of the current study is to determine whether an IF strategy increases the provision of ED-initiated BUP with referral for ongoing MAT. We will compare the uptake of ED-initiated BUP following a standard dissemination practice – provider education using a Grand Rounds-style educational format provided at the beginning of the baseline evaluation period.

To achieve this goal, we will arrange for a grand rounds presentation at each participating ED site during the institution's usual Emergency Medicine teaching conference, during the start-up 2 months at each site. Traditionally this is a 50-minute lecture with time for questions and comments. The content of the lecture will cover the scope of the opioid problem, ED specific facts related to the epidemic, potential models of intervention including the results of publications outlining the efficacy of ED-initiated BUP.[7] We will ensure that results are presented regarding the 30 day outcomes of various strategies including referral, brief intervention and facilitated referral and ED-initiated BUP with referral for ongoing MAT in an office-based primary care setting.

11.2 Implementation Facilitation

IF activities will begin in the IF period and continue through the IF evaluation period.

11.2.1 Overview of the Implementation Facilitation

IF will be based on a manualized program developed by Kirchner and colleagues[8] that has had significant impact on implementing healthcare practices in clinical settings. The LN will modify the Kirchner manual during the conduct of the study based on their facilitation experiences. Building on the mixed-methods analysis conducted during the formative evaluation, we will use the PARiHS framework to tailor the IF for site-specific needs. The facilitators and barriers identified by administrators, providers, and patients and will be characterized according to the PARiHS sub-elements of patient and clinical experience (communication, knowledgeable and empathetic providers), receptive context (resources to provide addiction treatments), and culture (value of team-based approach) identified. As described below, PARiHS will be used to further explicate and design the IF, guide the ongoing formative evaluation, and revise the strategy in an iterative manner to improve implementation success. The individual components (Table 1) of IF are described below.

11.2.2 Formative Evaluation

We will conduct a three-stage formative evaluation using mixed-methods[35] (qualitative and quantitative) to identify **evidence, context**, and **facilitation-related factors** impacting the provision of ED-initiated BUP with referral for ongoing MAT in the community and use these data to tailor, refine, monitor and evaluate the effectiveness of the IF. Given that formative evaluation techniques will be used during the IF period continuing into the IF evaluation period we will describe the methods in detail here and refer back to this section as needed.

Formative evaluation is a widely accepted implementation assessment approach designed to identify influences on the development, progress and effectiveness of implementation efforts.[19]

To increase the rate of provision of ED-initiated BUP and referral for ongoing MAT, we will use this formative evaluation to understand:

1. Site-specific practices
2. Determinants of these practices
3. Barriers and facilitators to practice change, and
4. Perspectives regarding IF. Using the PARiHS framework, we will employ a mixed-methods approach with 1) quantitative methods, using the Organizational Readiness to Change Assessment modified to focus on ED initiation of BUP (ED ORCA)[11] and Provider Readiness to Change scales, followed by 2) qualitative methods to develop an understanding of evidence, context and facilitation-related factors impacting the practice of ED-initiated BUP from the perspectives of diverse stakeholders including ED patients, nurses, social workers, physicians, NPs, PAs, pharmacists, and administrative directors (physician and nursing), and office-based physicians and OTPs representatives. We will provide feedback to all providers regarding the care of patients with OUD during the IF period.

11.3 Quantitative Data Collection and Analysis

The ORCA[11] instrument is based on the PARiHS framework, to evaluate evidence, context and facilitation-related factors impacting implementation ED-initiated BUP and referral for ongoing MAT. The ORCA has been applied to the evaluation of interventions intended to promote evidence-based practices, including addiction treatment, and predicts implementation efforts.[36-38] Baseline ORCA, and provider readiness and preparedness scores will be used to determine evidence and context related strengths and weaknesses in organizational readiness to implement BUP and referral and to tailor the IF. Other process measurements will include the number of ED providers attending the initial educational session, participating in the BUP training courses, obtaining a DATA 2000 waiver, proportion of eligible patients receiving ED-initiated BUP, provider skill and adherence to critical actions on initiating BUP treatment with referral to ongoing MAT; and organization data including integration of materials into EHR and proportion of patients with ED-initiated BUP who are successfully linked to office-based BUP providers and/or OTPs. EHRs will be queried for each enrolling shift to determine potential ICD 10 codes identifying patients that were potentially eligible for ED-initiated treatment and/or referral.

ED Organizational Readiness: We will use the ORCA with wording to reflect ED-initiated BUP to measure factors impacting the rates of ED-initiated BUP. This 5-minute survey is based on the PARiHS framework and asks the respondent to rate local factors related to evidence, context and facilitation on a 5-point Likert scale from strongly disagree to strongly agree. Facilitation questions will be omitted from the baseline assessment since this part of the intervention will not have taken place.

Community Providers Organizational Readiness: We will use the ORCA to measure factors impacting the care of the study participants with ED-initiated BUP. This asks the respondent to rate local factors related to evidence, context and facilitation on a 5-point Likert scale from strongly disagree to strongly agree. Facilitation questions will be omitted from the baseline assessment since this part of the intervention will not have taken place.

ED Provider Readiness and Preparedness: We will use change rulers, among appropriate providers, to assess readiness and preparedness to provide ED-initiated BUP.[39] Stage of change

assessments have been validated and have been used in the field of addiction and mental health to assess readiness to adopt evidence-based treatments.[40-43]

The change rulers will independently assess, on a 0-10 scale each provider's:

1. Readiness to provide the intervention
2. Preparedness to provide the intervention

Community Treatment Provider Readiness: We will use change rulers, among appropriate providers, to assess readiness and preparedness to provide care for study participants who have received ED-initiated BUP among office-based providers of BUP and representatives of OTPs.[39]

The change rulers will independently assess, on a 0-10 scale each provider's:

1. Readiness to provide the intervention
2. Preparedness to provide the intervention

Readiness rulers will be completed at the beginning and end of the IF period and after the 12 month IF evaluation period. ED and community provider ORCA assessments, including parts 1 and 2 (evidence and context assessment), will be completed at the beginning of the IF period. An ORCA that includes a facilitation assessment will be administered at the end of the IF period and at the end of the IF evaluation period, 12 months later. All readiness rulers and ORCA documents will be administered as survey instruments to ED and community providers and staff.

11.4 Qualitative Data Collection and Analysis

At each of the four ED study sites, we will conduct focus groups with a purposeful sample of key stakeholders at multiple distinct stages of our project: during the first month of the IF period, approximately at the 4th-5th months of the IF period and nearing completion of the IF evaluation period. However, data collected during focus groups and during the formative evaluation is part of an iterative process, and therefore additional focus groups, one-on-one phone interviews and email correspondence take place as needed. Purposeful sampling is a well-established method in qualitative studies and is designed to identify study participants who have direct experience with or knowledge of the phenomenon of interest, in this case OUDs and ED-initiation of BUP with referral for ongoing MAT. We have chosen to use focus groups given their suitability for generating data from multiple perspectives regarding the organizational and individual level factors impacting complex processes whereby the group interaction is anticipated to stimulate unique ideas.[35] We will enroll a variety of participants including ED patients, nurses, social workers, physicians, NPs, PAs, pharmacists, physician and nursing directors at each ED site and office-based BUP providers and representatives from OTPs to allow for evaluation of processes from multiple perspectives (triangulation). Focus groups will be conducted with approximately 4-8 study participants and representation from each of the stakeholder categories.[44] We will conduct focus groups and utilize the Rapid Assessment Process,[44] a type of participatory action research using intensive, team interaction and multiple cycles of data collection followed by data review and analysis. It is estimated that approximately 8-10 events will occur until themes begin to repeat. This process allows for results to be used for planning, monitoring and evaluating activities when prolonged fieldwork usually associated with traditional qualitative research is not possible. Recognizing our aggressive time schedule we will have ample time to schedule times early so that we are ready to conduct them immediately upon entry into the IF period.

11.5 Development of Focus Group Questions

The focus group guides will be informed by the PARiHS framework and include "grand tour" questions designed to establish rapport and elicit open-ended responses. Probes will be used to understand specific details of those experiences and allow for clarification of ideas. We will design and pilot test these guides with study participants and key informants from the participating NIDA CTN nodes and refine as indicated.

11.6 Verbal Consent for Focus Group Participants

Focus group participants comprised of ED and Community providers, staff, and patient s will be provided with an IRB-approved verbal consent including all significant elements of the study. We will work with the EDs to provide staff assurances that their participation in the research will in no way affect their employment status either positively or negatively. Focus groups will be voluntary and information collected for research purposes will not become part of staff's personnel records. The verbal consent for the ED and Community staff will outline these assurances. For the recording of the focus group sessions, participants will be made aware during the verbal consent process that the sessions will be digitally recorded and, for patient s, that the nature of these sessions will involve participants speaking about information regarding their health status and opioid use. Focus group participants will also be made aware that they will complete a brief demographic survey.

11.7 Conduct of Focus Groups

Trained and qualified personnel will conduct focus groups at each ED site during the IF period and the IF evaluation period. The focus groups will be recorded and professionally transcribed for qualitative analysis. Study participants will also complete a brief demographic survey. Using directed content analysis[49], we will analyze the data with a multi-disciplinary group with experience in qualitative methods, which will include Addiction Medicine physicians, ED Physician, and a health service researcher. Data will be entered into and organized using Atlas.ti software, or something comparable.

We will share our results with study participants (participant confirmation) to ensure that we have accurately understood and represented stakeholder's perspectives and experiences. Consistent with prior studies, we will plan to generate a summary of the findings to share with participants via email and conference calls for their feedback. We will use data reduction strategies to sharpen, sort, focus, discard and organize data in a way to draw inferences regarding the implementation interventions in place at each ED site. These data will directly inform the site-specific IF and be used to evaluate the facilitation process and outcomes. This will be an iterative process. We will develop a template summary of data at each ED organized by evidence, context and facilitation-related factors. At the completion of all 4 ED sites IF periods we will create a matrix across all sites and respondents to understand the major issues with regard to implementation across sites that can be disseminated to the Emergency Medicine community.

11.8 Administration of BUP During IF Evaluation Period

The following procedures will be incorporated into the training of ED providers during the IF period. We will assess fidelity to the procedures (adherence) using a critical action checklist. The checklist will include confirmation of documentation of: urine toxicology and liver function tests obtained; patient participant meets criteria for DSM-5 moderate-severe opioid use disorder; urine positive for opioid; formal assessment of Clinical Opioid Withdrawal Scale (COWS); ED-initiated BUP provided; BUP education and induction instructions provided; and referral for ongoing medication assisted treatment. Education regarding BUP and Induction is provided; and Appointment for ongoing MAT provided. Providers will be instructed to induce patients onto BUP using an induction and stabilization protocol that has been well tolerated by patients in our previous study.[7] Buprenorphine induction will take place in the ED or unobserved, and should be based on the study patient's level of opioid withdrawal as measured by the COWS. Depending on the study patient's level of physical dependence, the goal of the induction will be 8mg on the first day and 16mg on subsequent days. Study patients will receive a dose in the ED if they exhibit moderate to severe withdrawal on the COWS and will leave with a prescription for the daily doses needed prior to their scheduled follow-up appointment, and their significant others, will be provided with instructions for unobserved induction if their COWS score indicates less than mild withdrawal (Mild 5-12) at the time of the ED visit. Unobserved induction has become common practice[50] and was used successfully in our prior study of ED-initiated BUP. Unobserved induction will also have a goal of 8mg on the first day. In an effort to maximize retention and abstinence achievement during the induction we will instruct ED providers to provide study patients with a scheduled appointment for follow up with an office-based BUP provider or an OTP within 96 hours of their ED visit.

11.9 Referral for Continuation of MAT

1. Baseline evaluation period: participants will be referred based on existing clinical practice at each site. The study will not inform practice.

2. IF and IF evaluation period: During the IF, linkages will be established with all community stakeholders offering MAT including office-based providers of BUP and OTPs. Referral options will be offered according to insurance status, study patient preferences and availability.

11.10 Additional Components of Implementation Facilitation

EXTERNAL FACILITATORS

IF involves external facilitators working with local champions to facilitate multiple inter-related interventions designed to promote implementation of the evidence-based clinical practice tailored to the clinic specific needs and applied as needed over the course of the study protocol. The external facilitators in the current study assisted by trained and qualified personnel are expected to be a content experts who possesses superior communication and interpersonal skills and flexibility who will provide training, coach and mentor local champions, and encourage the exchange of ideas within and among sites.

LOCAL CHAMPIONS

The external facilitators in collaboration with trained and qualified personnel will spend the baseline phase of the project working with the local site PIs to identify local champions related to the ED and to community opioid treatment providers and programs. It is essential that local ED

champions work clinically in the ED and are seen as opinion leaders by the staff. The local champion(s) will be a respected physician(s) with a self-declared interest in treatment of OUD who expresses interest in promoting ED-initiated BUP with referral for ongoing MAT.[51] Following an in-person orientation and trainings the external facilitators in collaboration with trained and qualified personnel will provide external facilitation including bimonthly phone meetings for the duration of the IF. The Project Director or designee will take notes during these meetings, capturing information on challenges, barriers, facilitators and strategies. This information will be integrated into the formative evaluation, described in section 11.0.

PROVIDER EDUCATION AND ACADEMIC DETAILING

Academic detailing involves trained clinician consultants visiting other clinicians to share unbiased information about patient assessment and treatment with the goal of improving quality of care.[8] All providers involved in the implementation will be offered dedicated educational sessions on OUD and provision of BUP training, specifically tailored to each provider's tasks based on the initial formative evaluation and potentially modify, remove or add strategies to enhance implementation.

We will address practical issues such as efficient use of the electronic medical record for prompts, easily available procedures and check lists such as DSM-5 criteria for moderate/severe dependence, the COWS to determine whether BUP can be administered at the time of visit or as a take home, follow up procedures for appointments and patient monitoring strategies by ED follow up nurses, etc. We will share protocols for integration that have been developed at the Yale-New Haven Hospital and elsewhere. The external facilitators in collaboration with trained and qualified personnel will be primarily responsible for providing the content or identifying local site content experts who can deliver interactive training sessions. Training strategies will be based on adult learning theory and include didactic presentations on the effectiveness and safety of prescribing BUP and skill's based practice sessions. These sessions will focus on small group activities to promote the education of providers regarding the use of Motivational Interviewing (MI), brief interventions, and medication.

We will focus on frequent brief presentations at regular scheduled provider meetings and educational offerings such as faculty meetings, during the routine weekly educational lectures, simulation sessions and retreats. We will provide meals/refreshments at trainings and tools and web-based resources such as pcssmat.org. We will offer opportunities and facilitate completing the DATA 2000 waiver for BUP prescribing. In addition, we will use the faculty/other provider list serves to provide continual information and feedback.

STAKEHOLDER ENGAGEMENT

Stakeholder Engagement will take place at the administrative, provider, and patient levels. Efforts at increasing engagement will be informed by the focus groups conducted during the initial formative evaluation and supported by the efforts of the local champions. This work will be informed by the Normalization Process Model[52] whereby the external facilitators will work to have the screening, diagnosis and practices associated with OUDs embedded into the everyday ED processes. Thus, normalizing the care into everyday real world practice.

TAILORING THE PROGRAM TO LOCAL SITE

Tailoring the Program to Local Site will occur as a result of the formative evaluations and will be informed by local site PIs and local champions.

PERFORMANCE MONITORING AND FEEDBACK

This will involve regular assessment of individual clinician performance and providing information about that performance.[8] We will work with ED directors and other members of the ED staff to identify the optimal outcomes to be tracked as well as how often and in what format feedback will be provided based on ED and provider-level data. Outcomes will include the rate of BUP prescribed for eligible patients and follow up information regarding the patient's enrollment in treatment as well as individual provider adherence to critical actions on a checklist for prescribing BUP. These continual improvement activities will be integrated into the department's continuous quality improvement and feedback initiatives, similar to other clinical quality and safety measures such as door to balloon times used for patients who present to the ED with acute coronary syndromes. Emergency providers are accustomed to receiving daily operating reports such as patient length of stay, door to doctor and time to decision making. It is part of their world, and this will be integrated into their processes. The external facilitators in collaboration with trained and qualified personnel will provide additional training or educational booster sessions for sites with low implementation and those requesting such services.

FORMATIVE EVALUATION

a) **Implementation-focused formative evaluation** will occur during the implementation and focus on the discrepancies between the implementation plan and its operationalization. Examples of processes that will be examined include: 1) number and types of educational training sessions attended by staff, 2) number of staff who view the audit and feedback reports, and 3) number of champions that attend facilitation meetings.

b) **Progress-focused formative evaluation** meetings**,** led by trained and qualified personnel, will monitor achievement of implementation goals and performance targets to identify blocked progress, allowing steps to be taken to optimize the intervention. These meetings will focus on barriers and strategies to address identified barriers. Non-attendance of site participants will be documented and outreach through individual facilitation meetings, calls or emails will be initiated to assess for stalled progress and offer assistance.

c) **Interpretive formative evaluation** uses the data collected from the other formative evaluations and information collected at the end of the project regarding the participant experiences to clarify the meaning of successful or failed implementation and to enhance understanding of IF's impact. At the conclusion of the IF evaluation period (12 months), we will conduct an interpretive evaluation that will assess stakeholder views regarding (a) value of ED-initiated BUP with referral for ongoing MAT, (b) satisfaction or dissatisfaction with various aspects of IF, (c) reasons for ED level action or inaction with respect to ED-initiated BUP with referral for ongoing MAT, (d) additional barriers and facilitators, and (e) recommendations for further refinements. Information will also assess stakeholders' beliefs regarding IF's success and overall ''worth''.[19]

LEARNING COLLABORATIVE

A Learning Collaborative will be formed by inviting each of the sites' local champions, and other key stakeholders, to participate in monthly calls to promote shared learning regarding issues promoting and hindering implementation of addiction treatment. The local champions and key stakeholders will set the agenda and the calls will be facilitated by the external facilitators, in collaboration with trained and qualified personnel, and provide a dedicated time to discuss site-specific updates, challenges and possible solutions for implementation of addiction services. In addition, a listserv, similar to one established by Dr. Fiellin for the PCSS-buprenorphine,[53] will be

developed for use by all sites starting at the beginning of their IF phase. The external facilitators will make resources, protocol templates, materials and presentations available through the CTNs and ED professional organizations and other websites so they will be widely accessible at the end of the project.

PROGRAM MARKETING

Program Marketing will be conducted to promote awareness of the ED-initiated BUP clinical practice among all emergency providers and other ED providers, patients and community physician ED referrals, once we have completed the IF period in each site. The goal of the marketing will be to promote ED-initiated BUP with referral for ongoing MAT, and designed to initiate a motivational discussion between patients and providers. The ultimate marketing approach will be tailored to the needs of the stakeholders and include direct (e.g., “in-services” to discuss the treatment services and referral processes, flyers) and indirect (e.g., informal conversation) approaches. We will work with ED leaders and community based providers and programs to sustain all projects and spread the learning to other spokes related to the institutions, including newsletters, etc.

11.11 Provider Intervention to Enhance Motivation for Treatment

We will conduct skills based workshops open to all providers in the ED in an effort to motivate patients to accept a referral using our previous published brief intervention for severe opioid use disorder.[54] This intervention uses the principles of MI and is adapted from our work with hazardous and harmful alcohol use.[55,56] The active components involve: (1) Establishing rapport by asking permission to discuss the patient’s opioid use (2) Providing feedback, including stating concern for their drug use, and offering education regarding the use of medication-assisted treatment, such as BUP, and any harm reduction strategies; (3) Enhancing motivation by asking the patient how ready he/she is to accept a referral on a scale from 1-10, 1 being not ready at all and 10 being completely ready. If the patient chooses a number between 2-10, then we ask why he/she did not choose a lower number which in essence asks them why he/she wants to change. We then use reflective listening to elicit motivational statements for acceptance of a referral to a treatment program. During the training, we will use real case scenarios that were collected from our previous study. In addition to the skills based workshop we will provide access to our web-based, training program www.yale.edu/sbirt, Coach Vicky, which provides case-based practice of this 4-step brief intervention with a virtual patient with alcohol problems.[54]

12.0 STATISTICAL ANALYSES

This study aims to test two complementary hypotheses:

1. Implementation outcome: Across the entire study cohort, there will be a higher proportion of patients receiving ED-initiated BUP with referral for ongoing MAT in the IF evaluation period than in the baseline evaluation period.

2. Effectiveness outcome: Across the entire study cohort, there will be a higher proportion of patients engaged in formal addiction treatment on the 30th day post enrollment in the IF evaluation period than in the baseline evaluation period.

12.1 Definition of the Primary Effectiveness Outcome

The primary effectiveness outcome measure is based on a binary variable reflecting engagement in addiction treatment on the 30th day post enrollment. Engagement in addiction treatment will be defined as enrollment and receiving formal addiction treatment on the 30th day post enrolment, assessed by direct contact with the facility and/or treating clinician. Formal addiction treatment will be those treatments consistent with the American Society of Addiction Medicine's levels of care (1-4), and will include a range of clinical settings including office-based providers of BUP or naltrexone, OTPs, intensive outpatient, inpatient, or residential treatments.

12.2 Definition of the Primary Implementation Outcome

The primary implementation measure is based on a binary variable reflecting ED provider documentation of ED-initiated BUP with referral for ongoing MAT in the EHR as accessed by the RA.

12.3 Experimental Design

The experimental design will be a modified stepped wedge, as shown in Figure 2. The proposed study will be conducted at 4 ED sites, with a randomized order of when each site starts the protocol. Resource limitations preclude the use of more than 4 sites. At each of the study sites, approximately 25-30 of diverse ED providers will be engaged in the IF activities and evaluation research. The proposed sequence of baseline evaluation, IF and subsequent IF evaluation activities will take approximately 30-33 months at each site. Site selection criteria (e.g., a large prevalence of patients with OUD, population representative of US population, with sufficient referral networks for patients needing formal addiction treatment) will ensure the feasibility of providing the planned implementation research and generalizability of the study findings.

We plan to enroll a minimum of 240 (~120 during both the baseline evaluation period and IF evaluation periods = 240) patients with moderate/severe OUD at each of the 4 study sites, or approximately 960 patients total. The adequacy of this sample size to grant sufficient power for the two alternative hypotheses is discussed below. Enrollment will be monitored to ensure balance between the two enrollment periods at each site.

12.4 Recruitment and Enrollment

Patients with untreated moderate/severe OUD comprise in general less than 2% of the overall ED patient population. In our prior work conducted at a large inner city ED (Yale-New Haven Hospital), within 39 months we screened 71,742 patients to identify approximately 1200 patients with recent illicit opioid use. We estimate that each of the study sites will have an approximate volume of 5000 to 7000 ED patients per month, and will be able to enroll 10-12 study participants with moderate/severe OUD per month during the two 12-month enrollment periods.

12.5 Hypothesized Treatment Effect Sizes

We anticipate that during the baseline evaluation period, the rates of ED-initiated BUP treatment for OUD (primary implementation outcome) will be very low at each of the study sites (less than 15% of all eligible patients). Based on our prior work and other published research, we estimate that the rates of participation in a formal addiction treatment on the 30th day post enrollment (effectiveness outcome) will be approximately 25% for patients with OUD enrolled in the baseline evaluation period.

We anticipate that during the IF evaluation period, at each of the study sites, at least 25% of all eligible patients with OUD will receive ED-initiated BUP with referral for ongoing MAT. Based on our prior research, we also anticipate that approximately 40-50% of patients with OUD enrolled during the IF evaluation period will be engaged in formal addiction treatments at 30 days. The expected treatment effects of this section are summarized in Table 3 below.

12.5.1 **Table 3**: Hypothesized Probabilities of Success

Outcome	Baseline Evaluation Period	IF Evaluation Period
**Implementation**	< 15%	25%
**Effectiveness**	25%	40-50%

12.6 Power Analyses to Justify Sample Size

The power simulation to assess the adequacy of the sample size followed the method of Parzen[57] to simulate many vectors of (0,1) random variates with specified probabilities, a specified positive intraclass correlation (ICC) ρ, and assuming 240 patients enrolled at each of the 4 sites. The first 120 elements of each vector all had a common probability p_1, while the second 120 elements had a common probability p_2, where (p_1, p_2) are assumed probabilities of success in the baseline and IF evaluation periods, respectively. Each vector thus specifies in its first half a site's outcomes in the baseline evaluation period, while in the second half it specifies outcomes in the IF evaluation period. Four such vectors (one for each site) comprised the data for a single iteration of the simulation. Each element in each vector was assigned a month using the Study Timeline, shown in Figure 2[1]. (However, month by itself played no role in determining the probability of success of

[1] Treatment assignment is partially confounded with time of enrollment in the proposed design. If scheduling does not go the way it is supposed to (e.g., if site 1 IF period onset is delayed by 3 weeks), confounding will be complete.

any vector elements.) For each setting of the parameters (p_1, p_2, ρ), we generated 10,000 replicates (that is, 40,000 vectors) for simulation analysis.

The Stepped-Wedge model described in section 12.14.1 was used to analyze simulated data. Simulated power was taken to be the proportion of the 10,000 glimmix runs that had a significant type 3 p-value for arm (in this document the baseline and IF evaluation periods are referred to as "arms") at level 0.05, one-tailed. There is no attempt to control for type I error across these two outcomes.

We simulated power for four parameter sets (scenarios), under both alternative and null hypotheses as indicated in Table 4.

12.6.1 **Table 4**: Scenarios Used to Simulate Power

Scenario	Hypothesis	P1 (Baseline)	P2 (IF)
**Implementation outcome with low p1**	Alt	0.05	0.25
**Implementation outcome with high p1**	Alt	0.15	0.25
**Efficacy outcome with low p2**	Alt	0.25	0.40
**Efficacy outcome with high p2**	Alt	0.25	0.50
**Implementation outcome with low p1**	Null	0.05	0.05
**Implementation outcome with high p1**	Null	0.15	0.15
**Efficacy outcome**	Null	0.25	0.25

12.7 Potential for Sites to have Baseline Probability of 0 for Primary Implementation Outcome

We inspected the power under these scenarios in Table 4 as a function of an assumed ICC. Except for the "Implementation outcome with low p1" scenario, which had very low baseline probability, the power curves (not shown) exhibit the check-mark shape (i.e., power that first decreases, then increases as ICC gets larger), typical of Stepped Wedge design power curves[2]. However, the "Implementation outcome with low p1" scenario power curve declines steadily as ICC increases. Extensive further simulations (also not shown) suggested that the declining power curve of the "Implementation outcome with low p1" was due to the fact that, when ICC is large and baseline probability is low, there are many sites with all-zero data at baseline. Although data sets with this property generally converge, they often have nonpositive definite estimated G matrices. The G matrix contains the estimated covariance parameters. For the simulations under discussion, this means that the estimated variance of the site random effect is zero. According to SAS documentation, if the G matrix is not positive definite, there is not enough variation in the response to attribute any variation to the random effect, while controlling for other factors in the model. Although there is nothing inherently wrong with the results when this occurs, one approach is to remove the corresponding random effect from the model. One of the advantages of using SAS procedures GLIMMIX or PROC MIXED is that the same answers result with or without the zeroed term in the RANDOM statement. (We confirmed that removing the site random effect from the model when it is estimated to be zero does not change the p-value of the treatment effect.)

[2] The check-mark shape is presumably due to the two sources of power in Stepped Wedge designs: across-site comparisons and within-site comparisons. The latter contributes more to power when the ICC is high.

This issue suggests that low baseline probability, when coupled with high ICC, increases the rate of non-positive-definite G matrices in GLIMMIX, and this, while not causing GLIMMIX to actually fail, apparently degrades power.

Because in this study it is likely the implementation outcome will have a low probability in the baseline evaluation period coupled with a large ICC, other analytical methods were identified that had better power characteristics than the Glimmix approach outlined above. One method is to substitute historical data for the relevant site(s) for the data in the "all zeros" arm. Problems with this approach are (1) there is no guarantee that the historical data will not be "all zeros", and (2) it is difficult to simulate the effect of this policy beforehand. Below we show two other potential solutions: (1) the "ChangeToOne" policy and (2) Bayesian analysis.

We present graphs showing behavior of proposed analysis methods under various scenarios. For the convenience of the reader, Table 5 lists in one place the graphs and their content. Figures with a "B" concern Bayesian analyses and are presented and discussed in the Appendix Bayesian Analysis. Terms displayed in Table 5 are clarified in the text.

12.7.1 **Table 5:** List of Figures and their Contents for the Power Analysis

Figure	Method	Hypothesis	Measure Displayed
**2**	ChangeToOne	Alt	Power
**3**	ChangeToOne	Alt	Conf intervals for p1, p2
**4**	ChangeToOne	Null	Test size
**5**	ChangeToOne	Null	Conf intervals for p1, p2
**B5**	Bayes	Alt	Power
**B6**	Bayes	Alt	Cred intervals for p1, p2
**B7**	Bayes	Alt	Cred intervals for p1-p2
**B8**	Bayes	Null	Test size
**B9**	Bayes	Null	Cred intervals for p1, p2
**B10**	Bayes	Null	Cred intervals for p1-p2

12.8 ChangeToOne Approach

Under the ChangeToOne policy, if a particular site-arm has "all zeros" data (that is, if all results for that site are zero in either the baseline or IF evaluation period), a randomly-chosen one of the zeros is turned into a one. It is hoped that moving the data back from the edge of the outcome space will spare the analysis method from having to deal with phenomena such as estimated variances of zero. Note that, under the alternative that the control arm has a lower success probability than the treated arm, this change will tend to move the arms closer together, and thus can be considered conservative. This rather naïve approach actually turns out to have a surprisingly beneficial effect on "Implementation Outcome with low p1", without appreciably

changing power for the other scenarios (in which the probability of an “all zero” site-arm is very low, so the “change” policy has an effect only seldom).

Figure 2 shows power as a function of ICC for the 4 “Alt” scenarios of Table 4. A horizontal reference line at 0.8 (80% power) separates the (0.05, 0.25 – Implementation Outcome with low p1) and (0.25, 0.50 – Efficacy Outcome with high p2) scenarios from the other two, which have low power. The rate of convergence failure was very low in these simulations: considerably less than 1% for each scenario (not shown). Each data point in Figure 1 is labeled with the rate of non-zero variance estimates. This is low when the ICC = 0 (in which case the variance of the site random effect is actually zero), but fairly high elsewhere.

12.8.1 **Figure 2:** ChangeToOne Power Analysis; Alternative Cases

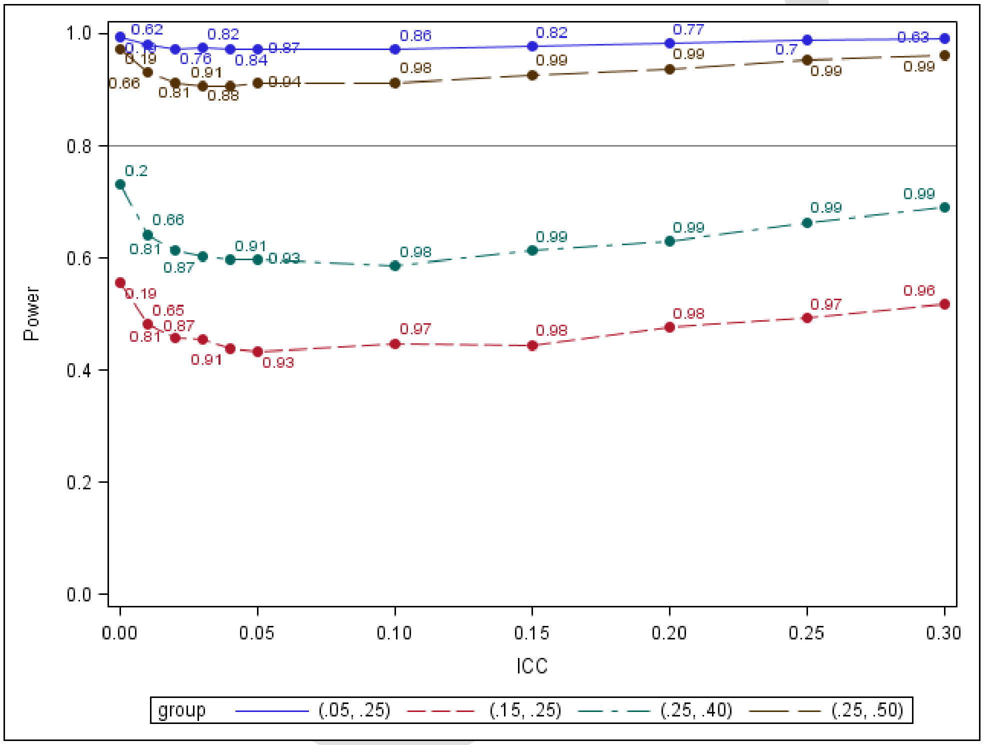

We use LSMEANS to aid intuition concerning the precision to expect from these analyses. For the current application, the LSMEAN statement estimates, for each arm separately, the probability of success at the average time (roughly month 22). More specifically, this is a transform of a linear combination of the fixed effects. Random site effects are taken into account (via the variance of the estimated betas) when calculating confidence limits. Figure 3 shows the estimated probability, with 95% confidence intervals, for the baseline arm (black solid) and the IF arm (red dashed). The point estimates and confidence limits are averaged across the iterations of the simulation. Increasing ICC widens the confidence intervals considerably.

12.8.2 **Figure 3**: ChangeToOne Confidence Intervals for p1, p2; Alternative Cases

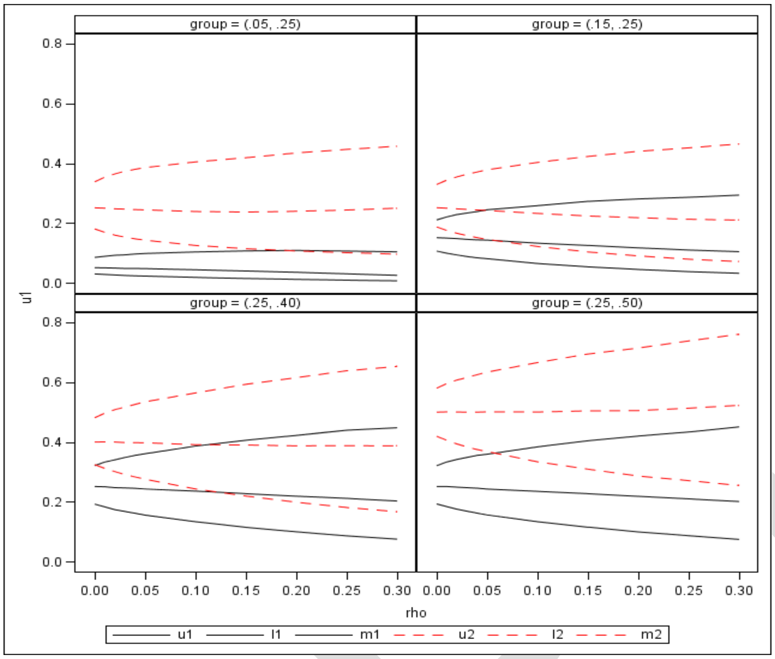

Figure 4 gives simulated test size (i.e. under the null hypothesis) at $\alpha = 0.05$, 1-tailed for the last 3 scenarios of Table 4, calculated on the basis of 10,000 iterations. A horizontal reference line marks 0.05, and the Y-axis scale goes from 0 to 0.1. Test sizes are tolerable, if not perfect.

12.8.3 **Figure 4**: "ChangeToOne" test size; Null Cases

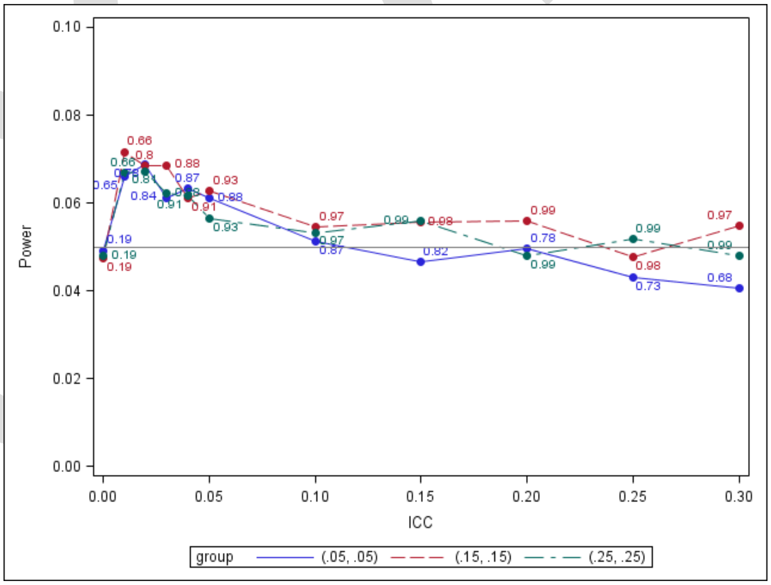

Figure 5 gives LSMEAN estimates of the probabilities of success in the "null" scenarios of Table 4. As expected, the two arms agree as to success probabilities in this case. Again, increasing ICC widens the confidence intervals.

12.8.4 **Figure 5**: "ChangeToOne" Confidence Intervals, p1, p2; Null Cases

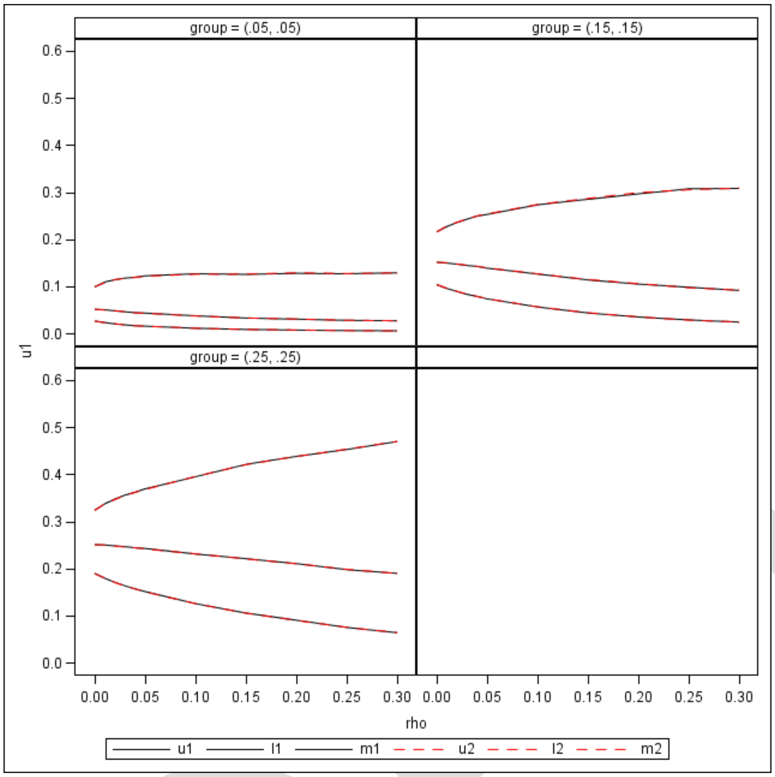

12.9 Bayesian Approach

We also considered a Bayesian approach, which has its own attractions and drawbacks. We detail these in an Appendix Bayesian Analysis. We propose to use the "ChangeToOne" approach for the primary analysis, and relegate the Bayesian approach to a secondary supporting analysis that we do not discuss here.

12.9.1 Summary of Power Simulations

Under the most optimistic scenarios investigated (Implementation Outcome with low p1 and Efficacy Outcome with high p2) and ICC < 0.3 (and perhaps higher), power for the envisioned design exceeds 0.8 under the ChangeToOne policy and Bayesian approach. Other scenarios featuring less separation between groups have lower power.

Having sites with "all zeros" in the baseline arm degrades power if you take no corrective action. The "ChangeToOne" policy seems a simple and attractive remedy.

A Bayesian approach with diffuse priors leads to conclusions similar to those from the ChangeToOne policy (see Appendix Bayesian Analysis).

Except for the credibility intervals for $p_2 - p_1$, increasing ICC degrades precision.

12.10 Missing Data

Patients lost to follow-up will not be counted as successes for either primary outcome, and, because counted as failures, will not generate missing data. Accordingly, we ignore the possibility

of loss to follow-up in the power calculations, and will assume that losses are failures for the purposes of the actual data analysis.

Several strategies will be implemented to minimize the likelihood and the rate of potential missing data in the proposed study. Timely data entry combined with frequent, planned, and scheduled evaluation of data completeness reports will trigger protocols for tracking and obtaining missing data. In our previous research, we developed patient tracking and communication protocols that resulted in very low rates of missing data. These filed tested procedures with proven effectiveness, with the necessary site specific adjustments, will be disseminated across all study sites.

12.11 Controlling Type 1 Error in Primary Analyses

In this study, we have defined two primary aims, one for implementation and one for efficacy. As this study is designed as a Hybrid Type 3 Effectiveness-Implementation study, the implementation is the main outcome, and so when measuring success of the study, the implementation outcome will take precedent over the effectiveness outcome, which will be considered secondary. Therefore, considering the effectiveness aim as secondary, it is not necessary to put in procedures to control the type 1 error across multiple outcomes. We also do not anticipate multiple-comparison adjustments when performing secondary analyses, but instead will be mindful of the multiple testing problem, thus report secondary findings as noteworthy hypothesis-generating results only when their p-values are considerably smaller than 0.05.

12.12 Interim Monitoring

No interim looks at primary or secondary outcomes are planned for this study. However, a power and sample size recalculation will be performed based on the primary outcome rates and enrollment rates observed in the baseline evaluation period. The power and sample size recalculation will be done no earlier than the end of the first site's baseline evaluation period.

12.13 Planned Secondary Analyses

We plan to perform the following secondary analyses:

Implementation Outcomes to compare the baseline evaluation and IF evaluation periods.

- Fidelity to a critical action checklist relating to the provision of ED-initiated BUP with referral for ongoing MAT in eligible patients
- Rates of enrolled patients with OUD receiving an appointment for opioid treatment provider/program upon ED discharge
- Number of ED providers receiving DATA 2000 training
- Number of clinicians providing ED-initiated BUP with referral for ongoing MAT
- ED provider readiness and preparedness ruler scores to initiate BUP and provide referral for ongoing MAT
- ED Organizational Readiness to Change Assessment (ORCA) scores relating to ED-initiated BUP with referral for ongoing MAT

- Community opioid treatment provider/program readiness and preparedness ruler scores to continue MAT for patients with OUD who have received ED-initiated BUP
- Community opioid treatment provider/program Organizational Readiness to Change Assessment (ORCA) scores relating to receiving patients with OUD who have received ED-initiated BUP.

Effectiveness Outcomes to compare the baseline evaluation and IF evaluation periods:

- Self-reported days of illicit opioid use (past 7-days) as measured by TLFB methods at 30 days
- Overdose events (past 30 days) captured by participant self-report, state medical examiner records, National Death Index and review of medical records.
- HIV risk taking behaviors (past 30 days) as measured by HIV Risk Taking Behavior Score
- Healthcare service utilization (past 30 days) measured by Health Services Utilization Form
- Rates of illicit opioid negative urines at 30 days

Data for the secondary implementation and effectiveness outcomes will be collected by trained RAs and using standardized assessment instruments (see Table 2) via face to face interviews with ED providers and patients with OUD enrolled in the proposed implementation research. The RAs will use tablet based software application to read questions and record responses directly to Advantage eClinical. Urine drug screen results will be obtained by testing urine samples using instant/rapid tests and entering the test result directly into Advantage eClinical via the tablet software application.

12.14 Statistical Methods

12.14.1 Primary Outcome Analysis

The study primary outcome is the implementation outcome. This outcome is defined as the rate of ED-initiated BUP with referral for ongoing MAT. The following base model will be used to compare the IF evaluation period to the baseline evaluation period in regards to the rates of ED-initiated BUP with referral for ongoing MAT.

$$\mathbf{logit}(\boldsymbol{p_{si}}) = \boldsymbol{\alpha} + \boldsymbol{\beta t_{si}} + \boldsymbol{\gamma f_{si}} + \boldsymbol{r_s}$$

where:

p_{si} is the probability of success of patient *i* at site *s*.

t_{si} is the calendar time of enrollment of patient *i* at site *s*. In the simulations reported below, *t* is expressed in integer months.

f_{si} is the indicator of whether patient *i* at site *s* is in the baseline (f=0) or IF (f=1) periods

r_s is the random effect of site s, where $r_s \sim N(0, \sigma)$

The following SAS code fragment estimates this model:

```
proc glimmix data = simul method = quad;
class arm (ref=”1”);
model z / trials = arm month / dist = binomial link = logit solution;
random intercept / subject = site;
estimate "trt effect" arm 1 -1 / cl;
lsmeans arm / cl ilink;
run;
```

In this model the γ estimate represents the estimated difference in the logit of the probability of success. The model will test a one-tailed hypothesis at a 0.05 level. The null and alternative hypotheses are:

$$H_0: \gamma = 0$$

$$H_a: \gamma > 0.$$

12.14.2 Effect Modification of Primary Analysis

Several covariates may have an effect on the primary outcome. At a minimum, sex, race and ethnicity will be considered as covariates in the model. They will be entered into the model in a stepwise manner using a p-value cutoff of 0.05. The most complicated model with the three minimal covariates would be:

$$\mathbf{logit}(\boldsymbol{p_{si}}) = \boldsymbol{\alpha} + \boldsymbol{\beta t_{si}} + \boldsymbol{\gamma f_{si}} + \boldsymbol{\theta_1} * \boldsymbol{sex_{si}} + \boldsymbol{\theta_2} * \boldsymbol{race_{si}} + \boldsymbol{\theta_3} * \boldsymbol{ethnicity_{si}}$$

$$+ \ \boldsymbol{\theta_4} \ * \boldsymbol{f_{si}} * \ \boldsymbol{sex_{si}} + \ \boldsymbol{\theta_5} \ * \boldsymbol{f_{si}} * \ \boldsymbol{race_{si}} + \ \boldsymbol{\theta_6} \ * \boldsymbol{f_{si}} * \ \boldsymbol{ethnicity_{si}} + \boldsymbol{r_s}$$

where sex, race and ethnicity are indicators for their respective variables. Additional appropriate covariates and parameters may also be considered in the above model.

12.14.3 Secondary Implementation Outcomes

- Fidelity (as measured by the check list score) to a critical action checklist relating to the provision of ED-initiated BUP with referral for ongoing MAT in eligible patients will be compared in the IF evaluation period to the baseline evaluation period. This will be done using standard models including mixed models, or other appropriate methodology.

COPY

- Rates of enrolled patients with OUD receiving an appointment for opioid treatment provider/program upon ED discharge. The IF evaluation period will be compared to the baseline evaluation period using the model described for the primary analysis in section 12.14.1.

- Number of ED providers receiving DATA 2000 training will be compared between IF evaluation period and baseline evaluation period using appropriate methodology. Permutation tests will be considered for this analysis.

- Number of clinicians providing ED-initiated BUP with referral for ongoing MAT will be compared in the IF evaluation period to the baseline evaluation period using count models. Models considered will be Poisson, zero-inflated Poisson, negative binomial and zero-inflated negative binomial. An offset will be considered for how long the provider has been trained.

- ED provider readiness and preparedness ruler scores to initiate BUP and provide referral for ongoing MAT will be compared between IF evaluation period and baseline evaluation period. This will be done using standard models including mixed models or other appropriate methodology. If the data do not follow a Gaussian distribution a modified Wilcoxon test [58] will be used.

- ED Organizational Readiness to Change Assessment (ORCA) scores relating to ED-initiated BUP with referral for ongoing MAT will be compared between IF evaluation period and baseline evaluation period. This will be done using standard models including mixed models or other appropriate methodology. If the data do not follow a Gaussian distribution a modified Wilcoxon test will be used.

- Community opioid treatment provider/program readiness and preparedness ruler scores to continue MAT for patients with OUD who have received ED-initiated BUP will be compared between IF evaluation period and baseline evaluation period. This will be done using standard models including mixed models or other appropriate methodology. If the data do not follow a Gaussian distribution a modified Wilcoxon test will be used.

- Community opioid treatment provider/program Organizational Readiness to Change Assessment (ORCA) scores relating to receiving patients with OUD who have received ED-initiated Bup will be compared between IF evaluation period and baseline evaluation period. This will be done using standard models including mixed models or other appropriate methodology. If the data do not follow a Gaussian distribution a modified Wilcoxon test will be used.

12.14.4 Effectiveness Outcomes

- The primary effectiveness outcome is defined as the rates of patient engagement in formal addiction treatment on the 30th day post enrollment. The model described in section 12.14.1 and 12.14.2 will be used to analyze this outcome.

- Self-reported days of illicit opioid use (past 7-days) as measured by TLFB methods at 30 days will be compared between IF evaluation period and baseline evaluation period using count models. Models considered will be Poisson, zero-inflated Poisson, negative binomial and zero-inflated negative binomial.

- Overdose events (past 30 days) captured by participant self-report, state medical examiner records, National Death Index and review of medical records will be compared using count models. Models considered will be Poisson, zero-inflated Poisson, negative binomial and zero-inflated negative binomial. An offset will be considered for how long a participant was in a given period if less than 30 days.

- HIV risk taking behaviors (past 30 days) as measured by HIV Risk Taking Behavior Score will be compared between IF evaluation period and baseline evaluation period. This will be done using standard models such as mixed models or other appropriate methodology. If the data do not follow a Gaussian distribution a modified Wilcoxon test will be used.

- Healthcare service utilization (past 30 days) measured by Health Services Utilization Form will be compared using count models. Models considered will be Poisson, zero-inflated Poisson, negative binomial and zero-inflated negative binomial. An offset will be considered for how long a participant was in a given period if less than 30 days.

- Rates of illicit opioid negative urines at 30 days. The IF evaluation period will be compared to the baseline evaluation period using the model described for the primary analysis in section 12.14.1.

For all secondary analyses, covariates such as sex, race and ethnicity will be considered if appropriate and feasible. Specifically, if model based approaches are used covariates will be considered. If non-parametric methods are used then the tests may be done at different covariate levels.

12.15 Exploratory Analyses

We will also evaluate a limited set of patient and provider characteristics for their potential effect on successful implementation and effectiveness outcomes. Study participant characteristics to be evaluated are:

- Gender
- Race/Ethnicity
- Health insurance status
- Age
- Primary drug (heroin vs prescription opioids)
- Reason for presentation such as seeking treatment for OUD or overdose
- Referral to office-based BUP provider versus OTP
- Pain Intensity and Interference (PEG scale)

ED characteristics such as size, location, existing substance abuse services and follow up resources as well as the range and number of addiction treatment services in the catchment area of the ED will be described, as well as ED provider characteristics such as age, gender, years, and level of training will be evaluated.

COPY

These analyses will utilize similar models as for the primary analysis, the MIXED models procedure repeated measures and generalized estimating equations (GEE), or other appropriate regression, clustering, and factor analytical tools to evaluate potential impact of site factors and patient characteristics on the primary implementation outcome and effectiveness outcome.

For cost effectiveness analyses, resource costs will include intervention costs incurred in the ED related to the studied intervention, (e.g., cost to provide ED-initiated BUP with referral for ongoing MAT, cost of buprenorphine), downstream medical costs and patient costs of treatment (e.g., time, transportation). Where relevant, we will convert duration of an activity to monetary values by multiplying by provider labor costs. The costs of all addiction and medical treatment (e.g., inpatient, outpatient, treatment center, medication) received by participants will be included in the cost calculations. This information will be collected by self-report through a health service utilization survey. Unit costs of substance abuse and medical treatment will come from the facility surveys or other published estimates.[59] Medication costs will be calculated from the average wholesale price plus the dispensing fee. We will collect Medicare reimbursement rather than Medicaid or commercial insurance amounts for relevant services in the facility surveys because Medicare reimbursement is most likely to reflect marginal costs of service provision. Incremental cost effectiveness ratios will be calculated, defined as $\Delta C/\Delta E$, where ΔC is the difference in costs and ΔE is the difference in effectiveness between the baseline evaluation period and IF evaluation period. Effectiveness is narrowly defined to the primary outcome – engagement in formal addiction treatment. The drawback of incremental CEA is that because no outcome is comprehensive, analyses do not allow one to directly compare interventions with different outcome measures. Yet, policymakers may still value this information when choosing among competing programs. Researchers often use the outcome Quality Adjusted Life Years (QALYs) to enable comparisons across interventions. In the case of OUD, many of the benefits accrue to individuals other than the individual being treated and would not be captured in this metric making this outcome less appealing. Because health care costs are typically highly skewed, we will consider several cost estimation models. We will not include monetized values of societal outcomes (i.e., reduced criminal activity) because inclusion of these monetized values of these outcomes in the numerator of the cost effectiveness ratio would lead to double counting of these outcomes (e.g., their monetized value would be counted in the numerator and then counted again as the value of being abstinent in the denominator of the cost effectiveness ratios). We will not include training or research costs because these costs would not be incurred in standard care. Our primary outcome will be cost effectiveness acceptability curves, which indicate the probability different implementation strategies are cost effectiveness at different willingness to pay threshold values of the studied outcome. For cost estimates which are subject to debate either because of known imprecision in the estimation procedures or lack of adequate information, we will conduct sensitivity analyses with the goal of explaining the ways in which different assumptions would impact study results.

We will use appropriate non-parametric, parametric, and analysis of variance statistical procedures to descriptively evaluate the key characteristics of each study site (e.g., patient flow indicators such as length of stay of treated and released patients, and demographic and drug use characteristics of patients with OUD presenting at each ED site, indicators of organizational level differences between the sites (e.g., the number ED providers, number/ratio ED providers DEA waivered to prescribe BUP), and to evaluate comparability of baseline characteristics among patient cohorts enrolled at each of the study sites and overall during baseline evaluation period and the IF evaluation periods across all sites.

13.0 TRAINING

Study RAs: A detailed training plan will be developed as part of the study Manual of Operations (MOP). Training in study-specific procedures and assessments will be provided by the Yale Project Director and affiliated staff members. The training will be conducted via web based, and in-person training sessions.

Provider: Qualified and trained personnel will provide training regarding ED-initiated BUP and ongoing community-based treatment for patient participants who have received ED-initiated BUP.

Non-Study specific training: Training will cover standard NIDA training for all CTN studies (e.g., Good Clinical Practices (GCP) and Human Subject Protection (HSP)), as well as protocol specific training as needed (e.g., safety procedures, data management and collection). All site staff will complete required Human Subjects Protection and Good Clinical Practices (GCP) training.

The training will include modules targeting all research team members conducted via web, telephone and in-person training sessions. All study staff will be required to complete any local training requirements per study site and IRBs. Further details are presented in the study Manual of Operations (MOP).

COPY

14.0 REPORTING AND MONITORING

14.1 Statement of Compliance

This study will be conducted in compliance with the appropriate protocol, current GCP, the principles of the Declaration of Helsinki, and all other applicable regulatory requirements. Participating sites must obtain written approval of the study protocol, consent form, other supporting documents, and any advertising for participant recruitment from the IRB of record for the study in order to participate in the study. Any amendments to the protocol or consent materials must be IRB approved before they are implemented. Annual progress reports and local Serious Adverse Event (SAE) reports will be submitted to the IRB of record for the study, according to its usual procedures.

14.2 Regulatory Files

The regulatory files should contain all required regulatory documents, study-specific documents, and all important communications. Regulatory files will be checked at each participating site for the regulatory documents compliance prior to study initiation, throughout the study, as well as at the study closure. The CCC will collaborate with the sites to be sure that all sponsor required regulatory documents have been uploaded into the Regulatory Tracking System (RTS).

14.3 Research Advisory Panel of California (California sites only)

Prior to initiating the study, the sponsor will obtain written approval from the Research Advisory Panel of California (RAP-C). Any planned research project to be conducted in California requiring the use of a Schedule I or Schedule II Controlled Substance as its main study drug as well as research for the treatment of controlled substance addiction or abuse utilizing any drug, scheduled or not (SAT) must be submitted to RAP-C for review and approval prior to study start-up. Study approval is based on review of the study protocol, consent form, and other pertinent study documents. Yearly reports will be provided to the RAP-C in order to obtain continuing study approval.

14.4 Informed Consent

An IRB waiver for written consent and instead elicit verbal consent to conduct all screening procedures to determine eligibility was approved by the IRB of record.

An IRB waiver for written consent and instead elicit verbal consent for the conduct of the focus groups was approved by the IRB of record.

The informed consent form is a means of providing information regarding the study to a prospective patient participant and allows for an informed decision about participation in the study. Given the now established safety and efficacy of the current ED-initiated BUP in our prior work, we do not view the proposed study as high risk. The consent form will include all of the required elements of informed consent. Each study site must have the study informed consent approved by the IRB of record for the study. A copy of the IRB-approved consent, along with the IRB study approval, must be sent to the Yale PI prior to the site initiation visit. Every patient participant to be included in the implementation or effectiveness outcomes is required to sign a valid, IRB-approved current version of the study informed consent form prior to enrollment into the study. The site must maintain the original signed informed consent for every participant in a locked,

secure location that is in compliance with their IRB and institutional policies and that is accessible to the study monitors. Every participant should be given a copy of their signed consent form.

Once eligibility is determined, the RA, who is knowledgeable about the study will explain the significant elements of the study to the potential participant. Included in the consent will be language allowing the ED RA to contact community-based providers and programs to determine the patient's engagement in formal addiction treatment status on the 30th day post enrollment. If the patient is able to converse and alert to person, place and time, the RA obtains signed, dated consent. A copy of the signed consent will be left with the participant. All persons obtaining consent must have completed appropriate training. The participant will be informed that their participation is voluntary and they may withdraw from the study at any time, for any reason without penalty. Individuals who refuse to participate or who withdraw from the study will be treated without prejudice. Study sites will be responsible for maintaining signed consent forms as source documents for quality assurance review and regulatory compliance.

The informed consent form must be updated or revised whenever important new safety information is available, or whenever the protocol is amended in a way that may affect participants' participation in the study.

14.5 Health Insurance Portability and Accountability Act (HIPAA)

Study sites may be required by their institutions to obtain authorization from participants for use of protected health information. Sites will be responsible for communicating with their IRBs or Privacy Boards and obtaining the appropriate approvals or waivers to be in regulatory compliance.

14.6 Investigator Assurances

Each study site must file (or have previously filed) a Federal Wide Assurance (FWA) with the DHHS Office for Human Research Protection setting forth the commitment of the organization to establish appropriate policies and procedures for the protection of human research subjects, with documentation sent to NIDA or its designee. Research covered by these regulations cannot proceed in any manner prior to NIDA receipt of certification that the research has been reviewed and approved by the IRB provided for in the assurance (45 CFR 46.103(b) and (f)). Prior to initiating the study, the principal investigator at each study site will sign a protocol signature page, providing assurances that the study will be performed according to the standards stipulated therein.

14.7 Financial Disclosure

All investigators will comply with the requirements of 42 CFR Part 50, Subpart F to ensure that the design, conduct, and reporting of the research will not be biased by any conflicting financial interest. Everyone with decision-making responsibilities regarding the protocol as well as all listed on the Investigator Agreements will be verified on an annual basis with the CCC that they are in compliance with their institutional reporting requirements.

14.8 Clinical Monitoring

14.8.1 Data collection and data completeness monitoring

The primary implementation outcomes at each study site will be based on ED provider documentation of ED-initiated BUP with referral for ongoing MAT in the EHR. Medical records will be accessed by an RA blinded from the index visit. The data will be entered into Advantage eClinical and accessed at each site by the RA.

The effectiveness data will be obtained by study RAs at each site by conducting the 30-day follow up assessment. All study patient participant-reported treatment engagement information will then be verified directly with the provider/program. Loss of follow up will be mitigated by having the RA schedule a 30-day post enrollment follow-up appointment prior to ED discharge and provide a printed appointment card.

At baseline we will collect information on provider wages and space costs to perform interventions to use in cost analysis. Unit process for other health care treatments will come from published national data.

Data completeness will be monitored weekly by review of aggregates/summaries of data completeness reports. The DSC will produce completeness summaries for DSMB meetings and for Trial Progress Reports, which are posted daily on the internet for perusal by study leadership.

The Lead Team (LT) comprised of the Lead Investigators (LI), Project Directors, and Site PI team will provide oversight for the study at all sites, with input from the Local Node PIs, Lead Node PIs and NIDA CCTN Scientific Officers and the DSC and CCC when required. Emmes will support study activities related to logistics (e.g., provision of study supplies and quality control) and data management. As the primary goal of this project is implementation, these monitoring efforts will focus on research (data collection) procedures of the research staff and not on implementation procedures, processes or outcomes of other entities. Qualified research personnel from the Local Node may assist the Lead Investigators with site management during the study to encourage and assess compliance with the study protocol, GCP guidelines, and to ensure the integrity of the study progress. This will take place as specified by the Lead Investigators and will occur as often as needed to help prevent, detect, and correct research problems at the study sites. The Lead Team will verify that study research procedures are properly followed and that site research staff members are trained and able to conduct the research protocol appropriately. If review of study documentation indicates that additional training of study personnel is needed, the Lead Investigators and/or Local Node will see that it is done.

Protection of the rights and welfare of study participants will be a vigilant process conducted by the research teams at all sites, at the Lead Node and by the sponsors of the research. Monitoring of the study sites will be conducted on a regular basis using a combination of NIDA-contracted monitors, local node monitors, and Lead Investigators staff. Site investigators will host periodic visits by monitors who will ensure all study procedures are appropriately conducted, and that study data are generated, documented and reported in compliance with the protocol, GCP, and applicable regulations. These monitors will audit at mutually agreed upon times, regulatory documents, case report forms (CRFs), and corresponding source documents for each participant. NIDA-contracted monitors will assure that submitted data are accurate and in agreement with source documentation where applicable and will also review regulatory/essential documents such as correspondence with the IRB. Areas of particular concern will be participant signed/dated consents, eligibility for study participation, protocol adherence, safety monitoring, IRB reviews

and approvals, regulatory documents, participant records, and principal investigator supervision and involvement in the study. Reports will be prepared following the visits and forwarded to the Lead Investigators and Site PIs the Lead Node, local node and NIDA CCTN.

Local QA monitor visits will take place as specified by the Lead Investigators and visits will occur as often as needed to help prevent, detect, and correct problems at the study sites. The Lead Investigator's team will work in collaboration with the local node staff to ensure that study procedures are properly followed and that site staff are trained and able to conduct the protocol appropriately. If the Lead Investigator's team review of study documentation indicates that additional training of study personnel is needed, node staff will communicate with the Lead Investigators to determine course of action.

14.9 Study Documentation

Study documentation includes but is not limited to, all case report forms, data correction forms, workbooks, source documents, monitoring logs and appointment schedules, sponsor-investigator correspondence, and signed protocol and amendments, Ethics Review Committee or IRB correspondence and approved consent form and signed participant consent forms.

Source documents include all recordings of observations or notations of clinical activities and all reports and records necessary for the evaluation and reconstruction of the study. All study information should be recorded, handled, and stored in a way that allows its accurate reporting, interpretation, and verification. Whenever possible, the original recording of an observation should be retained as the source document; however, a photocopy is acceptable provided that it is a clear, legible, and exact duplication of the original document.

14.10 Safety Monitoring

As buprenorphine, a marketed medication with prior known and labeled adverse reactions, is being used for its labeled indication in this study and is not being provided as a study medication but being used as a standard of care for treatment of opioid use disorder, the collection and reporting of safety events will be limited to those concerning hospitalization for all medical (including psychiatric) indications, Emergency Department visits, overdoses, and death of any participant enrolled in this study. All safety events, including designated SAEs will be presented to the DSMB.

14.10.1 Data and Safety Monitoring Board (DSMB)

An independent CTN DSMB will assure protection of study participants' safety while the study's scientific goals are being met. The CTN DSMB is responsible for conducting periodic reviews of accumulating safety and efficacy data. It will determine whether there is support for continuation of the study, or evidence that study procedures should be changed, or if the study should be halted, for reasons relating to the safety of the study participants, the efficacy of the treatment under study, or inadequate study performance (e.g., poor recruitment).

14.10.2 Reporting and Management

Any divergence from procedures and requirements outlined in the protocol will be classified a protocol deviation. A protocol deviation is an action (or inaction) that alone may or may not affect the scientific soundness of the investigation or seriously affect the safety, rights, or welfare of a study participant. In some cases, a protocol deviation may compromise participant safety,

participant rights, inclusion/exclusion criteria or the integrity of study data and is cause for corrective action to resolve the deviation and to prevent re-occurrence.

Protocol deviations will be monitored at each site for (1) significance, (2) frequency, and (3) impact on the study objectives, to ensure that site performance does not compromise the integrity of the study. The decision about whether a departure from the protocol will be designated as major or minor will be made by the Clinical Coordinating Center (CCC) and the protocol's Lead Investigators. The consequences will be specified, re-training or resolution conducted and participating sites should be informed as well as reviewed anonymously with all sites so similar deviations do not occur at other research sites

All protocol deviations will be recorded in the Advantage eClinical data entry system via the Protocol Deviation eCRF. Additionally, each site is responsible for tracking and reporting protocol deviations the IRB of record for the study, as required. The Data and Statistics Center (DSC) and the Lead Investigators must be contacted immediately if an unqualified/ ineligible study participant is enrolled into the study.

14.10.3 Confidentiality

By signing the protocol signature page, the investigator affirms that information furnished to the investigator by NIDA will be maintained in confidence and such information will be divulged to the IRB, Ethical Review Committee, or similar expert committee; affiliated institution; and employees only under an appropriate understanding of confidentiality with such board or committee, affiliated institution and employees.

14.11 Certificate of Confidentiality

The Lead Investigator will obtain a federal Certificate of Confidentiality (CoC) and will distribute it to all sites when received. No patients will be enrolled prior to receipt of the COC. A CoC helps researchers protect the privacy of human research participants enrolled in biomedical, behavioral, clinical and other forms of sensitive health-related research (e.g., drug use). Certificates protect against compulsory legal demands, such as court orders and subpoenas, for identifying information or identifying characteristics of a research participant. The department that issues the CoC will be advised of any changes in the CoC application information that may occur during the study, as required. Participating sites will be notified if CoC revision is necessary.

Participant records will be kept confidential by the use of study codes for identifying participants on CRFs, secure separate storage of any documents that have participant identifiers, and secure computing procedures for entering and transferring electronic data. All laboratory specimens, eCRFs, reports, and other data records will be identified by the participant identification study code that includes the site number, protocol number, and participant number. Research records will be stored in a locked cabinet. Only authorized individuals will have access to the study records. Participant information will not be released without written permission, except as necessary for monitoring by the NIDA-contracted monitors, local node monitors, lead node staff, or NIDA. By signing the protocol signature page, the investigator agrees that within local regulatory restrictions and ethical considerations, NIDA or any regulatory agency may consult and/or copy study documents in order to verify eCRF data.

14.12 Adverse Events (AEs) and Serious Adverse Events (SAEs)

For the purposes of this protocol, the collection and reporting of safety events will be limited to hospitalization for all medical (including psychiatric) indications, Emergency Department visits, overdoses, and death of any participant enrolled in this study. The study staff will be trained to monitor for and report all protocol-defined safety events including Serious Adverse Events. All safety events, including designated SAEs will be presented to the DSMB. SAEs that are specified for reporting will be reported to the IRB of record per the IRB's guidelines.

Reportable AEs will include:

- None

Reportable SAEs will include:

- Any death for any reason

All deaths, regardless of cause, will be captured for this study. These events will be identified through the participants' locator information or when possible through using state medical examiner records, National Death Index, and review of medical records. Deaths will be reported using the Adverse Event/Serious Adverse Event form set so that appropriate information surrounding the event can be gathered. It is the expectation that all *deaths will be reported in the EDC within 24 hours of site awareness. *Note: only death events that occur within the 30-day follow-up period will be recorded on the AE/SAE form set.

OTHER SAFETY EVENTS OF INTEREST

Hospitalization for all medical (including psychiatric) indications, Emergency Department visits and overdose events will not be captured as AEs or SAEs, but instead will be reported on study specific form(s).

All hospitalization events (including psychiatric indications) as well as Emergency Department visits will be captured for this study using self-report as well as review of medical records. These hospitalizations will be reported on the Health Services Utilization and ED Visits and Hospitalization CRFs. When possible, these forms will confirm the diagnosis for the hospital or ED visit and the information will be verified by study staff. The completion of these forms will indicate whether the information was gathered via self-report or medical record abstraction. This information will be collected at enrollment and at the 30-day follow up visit.

All overdose events will be captured for this study. Non-fatal overdose events will be captured through participant self-report on the Overdose CRF. This information will be collected at the enrollment visit as well as all follow-up visits for the study. Fatal overdose events will be captured through state medical examiner records, National Death Index, and review of medical records. *Fatal overdose events also qualify as a reportable SAE and will, therefore, be additionally reported on the AE/SAE CRF form set. *Note: only death events that occur within the 30-day follow-up period will be recorded on the AE/SAE form set.

OTHER EVENTS

Pregnancy is not exclusionary and will not be captured as the procedures for prescribing medication is per standard of care.

COPY

Incarceration that occurs on study will be reported on the Crime and Criminal Justice case report form but will not be considered an adverse or serious adverse event.

Loss of confidentiality related to participation in this study will be monitored at the site and will be reported on the Protocol Deviation eCRF as well as reported to the IRB of record per IRB requirements.

COPY

15.0 DATA MANAGEMENT AND PROCEDURES

15.1 Design and Development

This protocol will utilize a centralized Data and Statistics Center (DSC). The DSC will be responsible for development of the electronic case report forms (eCRFs) for tablet use, development and validation of the clinical study database, ensuring data integrity, and along with the Project Director will assist in training site investigators and staff on applicable data management procedures. Advantage eClinical, a web-based distributed data entry system will be implemented. This system will be developed to ensure that guidelines and regulations surrounding the use of computerized systems used in clinical research are upheld. The remainder of this section provides an overview of the data management plan associated with this protocol.

15.1.1 Data Collection

Data is collected at the study sites either through direct data entry at the study sites into Advantage eClinical or on source documents, which are entered at the study site into Advantage eClinical. In the event that Advantage eClinical is not available, the DSC will provide the sites with a final set of guided source documents and completion instructions. Data entry into the source documents and electronic Case Report Forms (eCRFs) should be completed according to the instructions provided and project specific training. The investigator is responsible for maintaining accurate, complete and up-to-date records, and for ensuring the completion of the eCRFs for each research participant.

15.1.2 Data Acquisition and Entry

Data entry into eCRFs shall be performed by authorized individuals and shall be entered into the Advantage eClinical system in accordance with the Advantage eClinical User’s Guide. Selected eCRFs may also require the Site Principal Investigator’s written signature or electronic signature, as appropriate. Electronic CRFs will be monitored for completeness, accuracy, and attention to detail throughout the study.

15.1.3 Site Responsibilities

The data management responsibilities of each individual site will be specified by the DSC and outlined in the Advantage eClinical User’s Guide.

15.1.4 Data Center Responsibilities

The DSC will 1) develop a data management plan and will conduct data management activities in accordance with that plan guide, 2) provide final guided source documents and eCRFs for the collection of all data required by the study, 3) develop data dictionaries for each eCRF that will comprehensively define each data element, 4) conduct ongoing data monitoring activities on study data from all participating sites, 5) monitor any preliminary analysis data cleaning activities as needed, and 6) rigorously monitor final study data cleaning.

15.1.5 Data Editing

Completed data will be entered into Advantage eClinical. If incomplete or inaccurate data are found, a data clarification request will be generated to the sites for a response. Sites will resolve data inconsistencies and errors and enter all corrections and changes into Advantage eClinical in accordance with the Advantage eClinical User's Guide.

15.1.6 Data Transfer/Lock

Data will be transmitted by the DSC to the NIDA central data repository as requested by NIDA. The DSC will conduct final data quality assurance checks and "lock" the study database from further modification. The final analysis dataset will be returned to NIDA, as requested, for storage and archive.

15.2 Data Training

The training plan for RA staff includes provisions for training on assessments, eCRF completion guidelines, data management procedures, the use of Advantage eClinical and the use of other computerized systems, as required.

15.3 Data Quality Assurance

To address the issue of data entry quality, the DSC will follow a standard data monitoring plan. An acceptable quality level prior to study lock or closeout will be established as a part of the data management plan. Data quality summaries will be made available during the course of the protocol.

COPY

16.0 STUDY TIMELINE

From time of approval of the final version of the protocol, the study team launched the study within approximately 6 months. The pre-launch activities included submitting and obtaining IRB approval, obtaining a Certificate of Confidentiality, data system development, site selection and preparation, initial investigator meeting and training, and completion of the Manual of Operations (see Study Timeline diagram in Section 5.3.1).

COPY

17.0 PUBLICATIONS AND OTHER RIGHTS

Per NIH Policy, the results of the proposed study are to be made available to the research community and the public at large. The planning, preparation, and submission of publications will follow the policies of the Publications Committee of the CTN.

COPY

18.0 SIGNATURES

SPONSOR'S REPRESENTATIVE (CCTN DESIGNEE)

Printed Name	Signature	Date

ACKNOWLEDGEMENT BY INVESTIGATOR:

- I am in receipt of version 4.0 of the protocol and agree to conduct this clinical study in accordance with the design and provisions specified therein.

- I agree to follow the protocol as written except in cases where necessary to protect the safety, rights, or welfare of a participant, an alteration is required, and the sponsor and IRB have been notified prior to the action.

- I will ensure that the requirements relating to obtaining informed consent and institutional review board (IRB) review and approval in 45 CFR 46 are met.

- I agree to personally conduct or supervise this investigation at this site and to ensure that all site staff assisting in the conduct of this study are adequately and appropriately trained to implement this version of the protocol and that they are qualified to meet the responsibilities to which they have been assigned.

- I agree to comply with all the applicable federal, state, and local regulations regarding the obligations of clinical investigators as required by the Department of Health and Human Services (DHHS), the state, and the IRB.

SITE'S PRINCIPAL INVESTIGATOR

Printed Name	Signature	Date

Clinical Site Name ______________________

Node Affiliation ______________________

COPY

19.0 APPENDIX: BAYESIAN ANALYSIS

To construct a Frequentist confidence interval for a parameter, one follows a recipe that generates from the data a region that, with specified probability, encloses the parameter. One tests at level 0.05 whether a parameter is or is not equal 0 by determining whether the 95% confidence interval for the parameter does or does not enclose 0.

In Bayesian statistics, the parameter is associated with a probability distribution. This distribution describes one's belief that the parameter could equal various numeric values. Credibility intervals, the Bayesian analogues to Frequentist confidence limits, are regions of the distribution that integrate to specific probabilities. A Bayesian analogue to testing whether a parameter is or is not equal 0 involves discovering whether the 95% credibility interval for the parameter does or does not enclose 0. A Bayesian analogue to power is the proportion of simulated data sets in which the 0.95-level credibility interval for the treatment effect does not enclose zero. (Equivalently for 1-tailed tests, the parameter differs from 0 if the integral of its distribution from 0 to infinity exceeds 0.95).

Figures B5 and B8 show the Bayesian power curves in the alternative and null cases we have been considering. These figures, based on simulations of 1000 iterations per scenario (the Bayesian method takes much longer – 8 hours for 250 iterations per scenario -- than the Frequentist approach – 4 hours for 10,000 iterations per scenario), are comparable to Figures 1 and 3, respectively. They indicate that the Bayesian approach may be a useful secondary analysis method for the primary hypotheses under discussion. Note, however, that although autocorrelation was a problem in only a fraction of a percent of the alternative-case iterations, about 27% of the null-case iterations generated SAS warnings that there was still significant autocorrelation after 500 lags.

The Bayesian approach undertaken here uses diffuse priors. Perhaps using historical data to estimate priors could lead to an increase in power, but this is not investigated here.

Figures B6-B7 show Bayes estimates for $p_1, p_2, \text{and}\, p_1 - p2$. The average credibility intervals for $p_1 \text{and}\, p_2$ are somewhat wider than corresponding confidence intervals. The reason for this is unclear. Although the treatment effect is an odds ratio, i.e. $p_2(1-p_1)/[p_1(1-p_2)]$ in both approaches, an attractive aspect of the Bayes MCMC method is that it is easy to get the posterior distribution of the simple treatment difference $p_2 - p_1$, as shown in Figure B7. The credibility interval for $p_2 - p_1$ seems less sensitive to ICC than other interval estimates investigated.

19.1 Figure B5: Bayes power; Alternative Cases

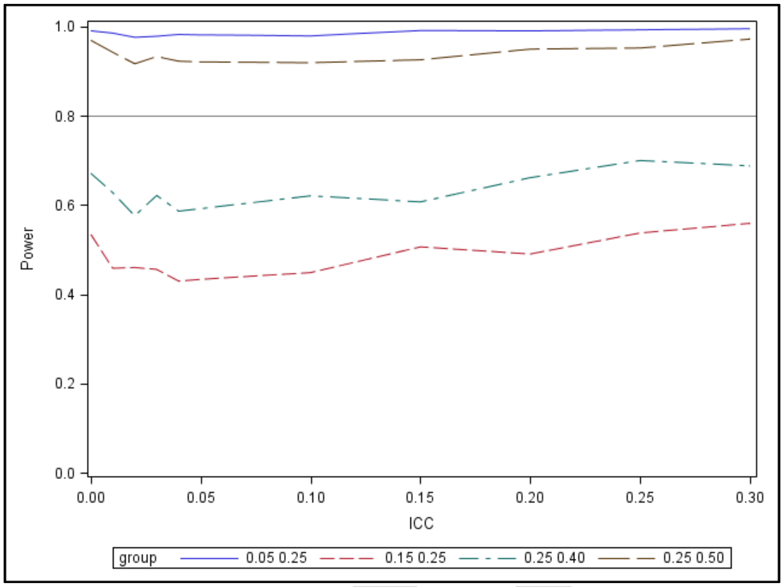

19.2 Figure B6: Bayes Credibility Intervals for p1, p2; Alternative Cases

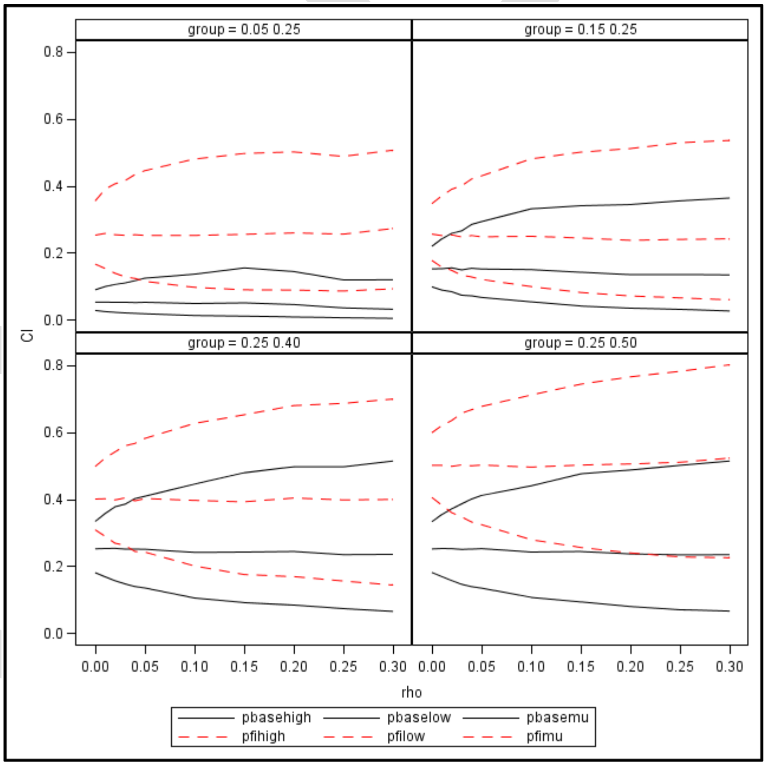

19.3 Figure B7: Bayes Credibility Intervals for p1-p2; Alternative Cases

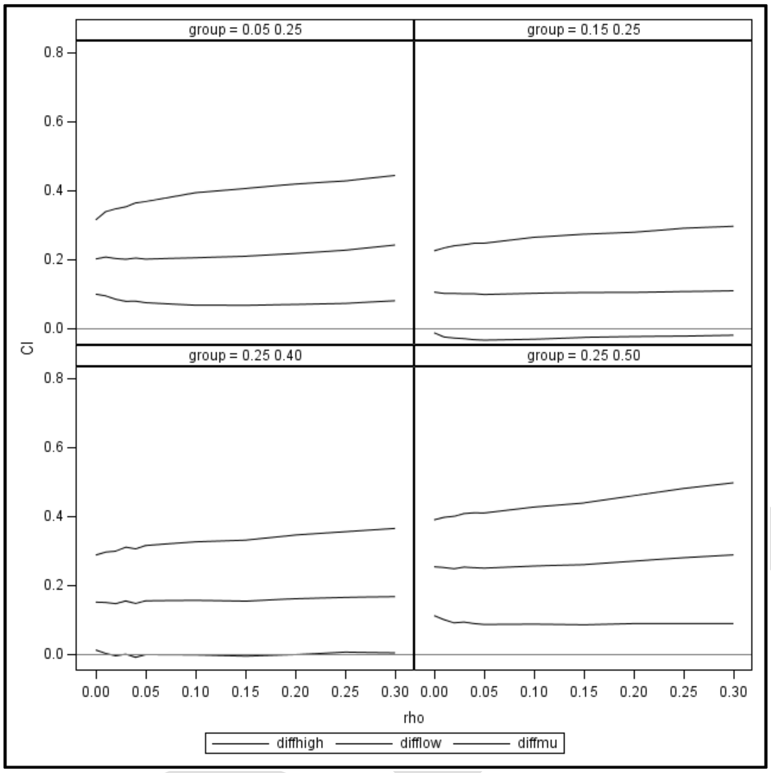

Figures B8-B10 give test size and credibility intervals under the “Null” scenarios of Table 4. Figures B8-B9 are comparable to their Frequentist analogues (Figures 4.-5, sections 11.8.3-11.8.4). As in Figure B7, the credibility interval for $p_2 - p_1$ (Figure B10) seems less sensitive to ICC than other interval estimates investigated.

COPY

19.4 Figure B8: Bayes Test Size; Null Cases

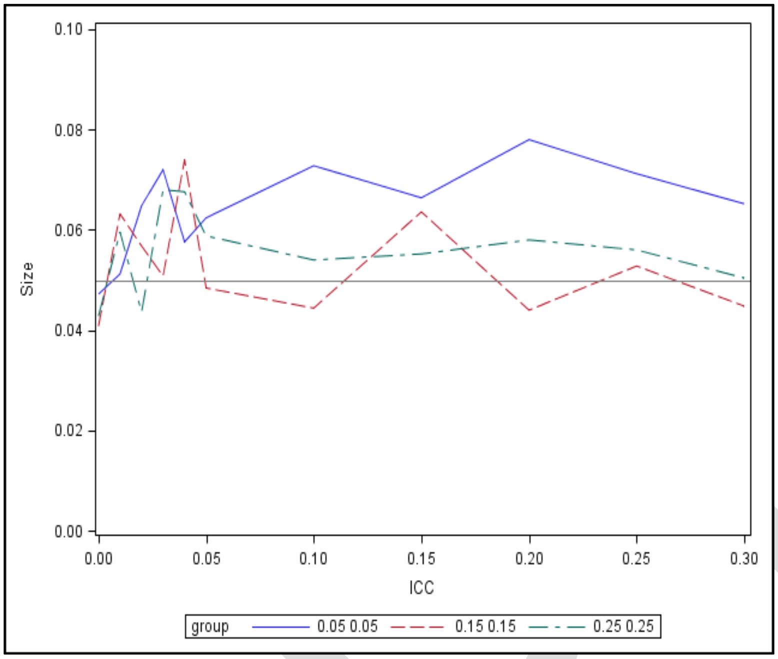

19.5 Figure B9: Bayes Credibility Intervals for P1, P2; Null Cases

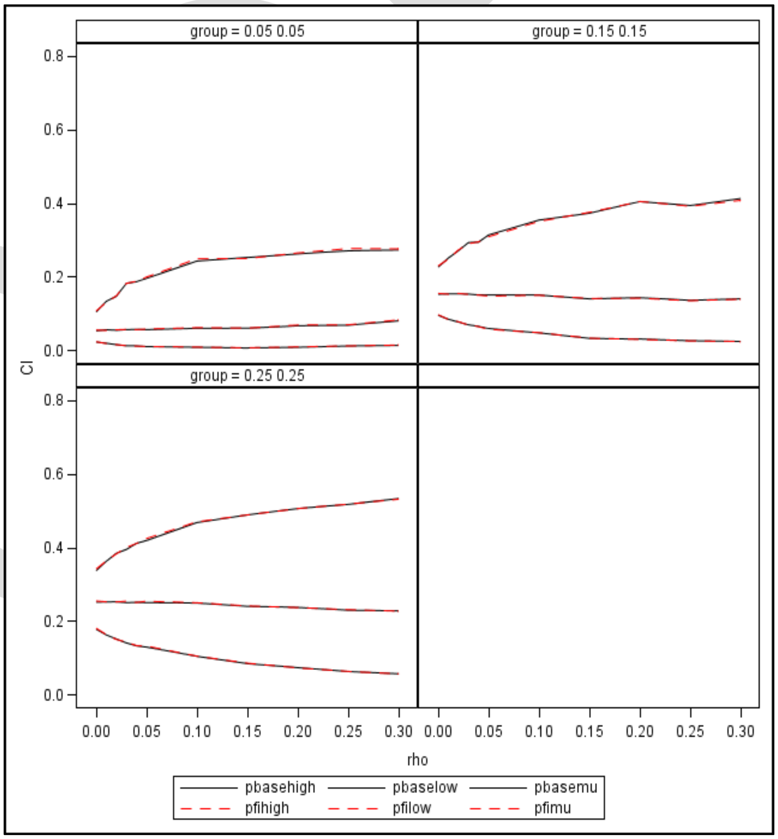

19.6 Figure B10: Bayes Credibility Intervals P1-P2; Null Cases

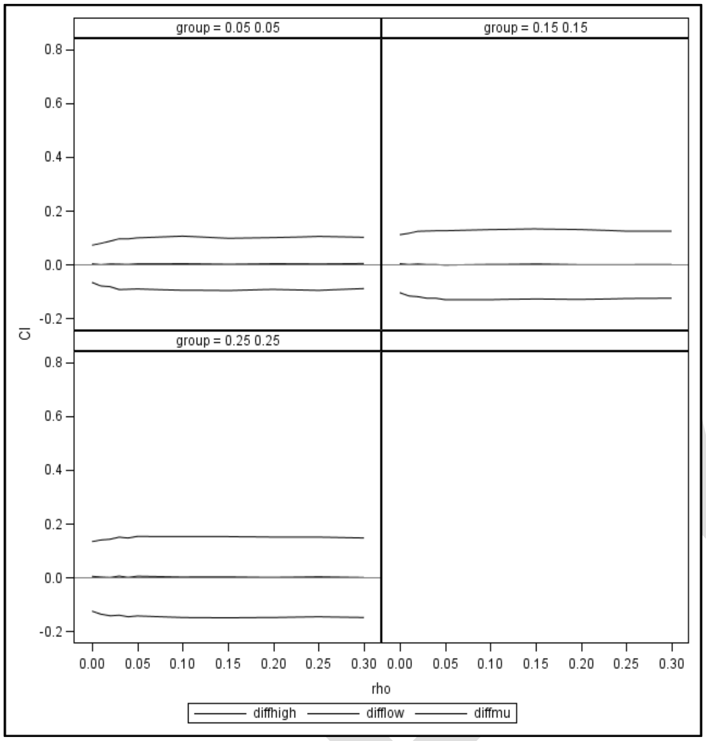

We document here the SAS code for the Bayesian approach, which employs the Markov Chain Monte Carlo method.

```
proc mcmc data = summsimul nbi = 1000 nmc = 10000 thin = 2 seed = 159

    monitor = (beta0-beta2 beta2_gt_0 p1 p2 pdiff) statistics = (summary intervals);
    parms beta0-beta2 0;
    parms sigma2 1;
    prior beta0-beta2 ~ normal(mean = 0, var = 1000);
    prior sigma2 ~ igamma(shape = 0.001, scale = 0.001);
    random b0 ~ normal(mean = 0, var = sigma2) subject = site;
    array p[2];
    p[treat+1] = logistic(beta0 + beta1 * 22 + beta2 * treat);
    pdiff = p2 – p1;
    eta = beta0 + beta1 * month + beta2 * treat + b0;
    pi = logistic(eta);
    model z ~ binomial(n = trials, p = pi);
    beta2_gt_0 = beta2 > 0;
    run;
```

20.0 REFERENCES

1. Brown CA, Lilford RJ. The stepped wedge trial design: a systematic review. *BMC medical research methodology.* 2006;6:54.

2. Mdege ND, Man MS, Taylor Nee Brown CA, Torgerson DJ. Systematic review of stepped wedge cluster randomized trials shows that design is particularly used to evaluate interventions during routine implementation. *Journal of clinical epidemiology.* 2011;64(9):936-948.

3. Hussey MA, Hughes JP. Design and analysis of stepped wedge cluster randomized trials. *Contemporary clinical trials.* 2007;28(2):182-191.

4. van Breukelen GJ, Candel MJ. Calculating sample sizes for cluster randomized trials: we can keep it simple and efficient! *Journal of clinical epidemiology.* 2012;65(11):1212-1218.

5. Baskerville NB, Liddy C, Hogg W. Systematic review and meta-analysis of practice facilitation within primary care settings. *Annals of family medicine.* 2012;10(1):63-74.

6. Volkow ND, Frieden TR, Hyde PS, Cha SS. Medication-assisted therapies--tackling the opioid-overdose epidemic. *N Engl J Med.* 2014;370(22):2063-2066.

7. D'Onofrio G, O'Connor PG, Pantalon MV, et al. Emergency department-initiated buprenorphine/naloxone treatment for opioid dependence: a randomized clinical trial. *Jama.* 2015;313(16):1636-1644.

8. Kirchner JE, Ritchie MJ, Pitcock JA, Parker LE, Curran GM, Fortney JC. Outcomes of a partnered facilitation strategy to implement primary care-mental health. *Journal of general internal medicine.* 2014;29 Suppl 4:904-912.

9. Curran GM, Bauer M, Mittman B, Pyne JM, Stetler C. Effectiveness-implementation hybrid designs: combining elements of clinical effectiveness and implementation research to enhance public health impact. *Medical care.* 2012;50(3):217-226.

10. Woertman W, de Hoop E, Moerbeek M, Zuidema SU, Gerritsen DL, Teerenstra S. Stepped wedge designs could reduce the required sample size in cluster randomized trials. *Journal of clinical epidemiology.* 2013;66(7):752-758.

11. Helfrich CD, Li YF, Sharp ND, Sales AE. Organizational readiness to change assessment (ORCA): development of an instrument based on the Promoting Action on Research in Health Services (PARIHS) framework. *Implementation science : IS.* 2009;4:38.

12. National Institute on Health. Implementation Science Information and Resources. 2015.

13. Kitson A, Harvey G, McCormack B. Enabling the implementation of evidence based practice: a conceptual framework. *Quality in health care : QHC.* 1998;7(3):149-158.

COPY

14. Harvey G, Kitson A. PARIHS revisited: from heuristic to integrated framework for the successful implementation of knowledge into practice. *Implementation science : IS.* 2016;11:33.

15. Rycroft-Malone J, Kitson A, Harvey G, et al. Ingredients for change: revisiting a conceptual framework. *Quality & safety in health care.* 2002;11(2):174-180.

16. Kitson AL, Rycroft-Malone J, Harvey G, McCormack B, Seers K, Titchen A. Evaluating the successful implementation of evidence into practice using the PARiHS framework: theoretical and practical challenges. *Implementation science : IS.* 2008;3:1.

17. Stetler CB, Damschroder LJ, Helfrich CD, Hagedorn HJ. A guide for applying a revised version of the PARIHS framework for implementation. *Implementation science : IS.* 2011;6:99.

18. VA Mental Health Queri. Implementation Facilitation Training Manual: Using External and Internal Facilitation to Improve Care in the Veterans Health Administration United States of America. In: Affairs DoV, ed. Vol Version 12016.

19. Stetler CB, Legro MW, Wallace CM, et al. The role of formative evaluation in implementation research and the QUERI experience. *Journal of general internal medicine.* 2006;21 Suppl 2:S1-8.

20. Baker R, Camosso-Stefinovic J, Gillies C, et al. Tailored interventions to overcome identified barriers to change: effects on professional practice and health care outcomes. *The Cochrane database of systematic reviews.* 2010(3):Cd005470.

21. Johnson KE, Coleman K, Phillips KE, et al. Development of a facilitation curriculum to support primary care transformation: the "coach medical home" curriculum. *Medical care.* 2014;52(11 Suppl 4):S26-32.

22. Anonymous. Implementing the PCMH: Practice Facilitation: Agency for Healthcare Research and Quality. 2015. Available from: http://pcmh.ahrq.gov/page/practice-facilitation. Accessed March 24, 2015.

23. Waiver authority for physicians who dispense or practice or prescribe certain narcotic drugs for maintenance treatment of detoxification treatment. *HR.* 2000;4365:122-127.

24. Substance Abuse and Mental Health Services Administration. Treatment CfSA. Clinical Guidelines for the Use of Buprenorphine in the Treatment of Opioid Addiction. Rockville, MD2004.

25. Fleming MF, Barry KL. A three-sample test of a masked alcohol screening questionnaire. *Alcohol and alcoholism (Oxford, Oxfordshire).* 1991;26(1):81-91.

26. Fleming MF, Bruno M, Barry K, Fost N. Informed consent, deception, and the use of disguised alcohol questionnaires. *The American journal of drug and alcohol abuse.* 1989;15(3):309-319.

27. Maisto SA, Clifford PR, Stout RL, Davis CM. Moderate drinking in the first year after treatment as a predictor of three-year outcomes. *Journal of studies on alcohol and drugs.* 2007;68(3):419-427.

COPY

28. Sobel L, Sobell M. Timeline follow-back: a technique for assessing self-reported alcohol consumption. In: Litten R, Allen J, eds. *Measuring Alcohol Consumption: Psychosocial and Biological Methods*. Totowa, NJ: Humana Press; 1992:41-72.

29. McLellan T, Zanis D, Incmikoski R. Treatment Service Review (TSR). Philadelphia: The Center for Studies in Addiction. In: Department of Psychiatry PVMC, The University of Pennsylvania, eds. Philadelphia, PA1989.

30. Rabin R, de Charro F. EQ-5D: a measure of health status from the EuroQol Group. *Annals of medicine.* 2001;33(5):337-343.

31. Kroenke K, Spitzer RL, Williams JB. The PHQ-9: validity of a brief depression severity measure. *Journal of general internal medicine.* 2001;16(9):606-613.

32. Darke S, Hall W, Heather N, Ward J, Wodak A. The reliability and validity of a scale to measure HIV risk-taking behaviour among intravenous drug users. *AIDS (London, England).* 1991;5(2):181-185.

33. Dwyer K, Walley AY, Langlois BK, et al. Opioid education and nasal naloxone rescue kits in the emergency department. *The western journal of emergency medicine.* 2015;16(3):381-384.

34. Fals-Stewart W, O'Farrell TJ, Freitas TT, McFarlin SK, Rutigliano P. The timeline followback reports of psychoactive substance use by drug-abusing patients: psychometric properties. *Journal of consulting and clinical psychology.* 2000;68(1):134-144.

35. Curry LA, Nembhard IM, Bradley EH. Qualitative and mixed methods provide unique contributions to outcomes research. *Circulation.* 2009;119(10):1442-1452.

36. Hagedorn HJ, Heideman PW. The relationship between baseline Organizational Readiness to Change Assessment subscale scores and implementation of hepatitis prevention services in substance use disorders treatment clinics: a case study. *Implementation science : IS.* 2010;5:46.

37. Hagedorn HJ, Stetler CB, Bangerter A, Noorbaloochi S, Stitzer ML, Kivlahan D. An implementation-focused process evaluation of an incentive intervention effectiveness trial in substance use disorders clinics at two Veterans Health Administration medical centers. *Addiction science & clinical practice.* 2014;9:12.

38. Owen RR, Drummond KL, Viverito KM, et al. Monitoring and managing metabolic effects of antipsychotics: a cluster randomized trial of an intervention combining evidence-based quality improvement and external facilitation. *Implementation science : IS.* 2013;8:120.

39. LaBrie JW, Quinlan T, Schiffman JE, Earleywine ME. Performance of alcohol and safer sex change rulers compared with readiness to change questionnaires. *Psychology of addictive behaviors : journal of the Society of Psychologists in Addictive Behaviors.* 2005;19(1):112-115.

40. Boudreaux ED, Sullivan A, Abar B, Bernstein SL, Ginde AA, Camargo CA, Jr. Motivation rulers for smoking cessation: a prospective observational examination of construct and predictive validity. *Addiction science & clinical practice.* 2012;7:8.

COPY

41. Haug NA, Shopshire M, Tajima B, Gruber V, Guydish J. Adoption of evidence-based practices among substance abuse treatment providers. *Journal of drug education.* 2008;38(2):181-192.

42. Aarons GA. Mental health provider attitudes toward adoption of evidence-based practice: the Evidence-Based Practice Attitude Scale (EBPAS). *Mental health services research.* 2004;6(2):61-74.

43. Amodei N, Lamb RJ. Convergent and concurrent validity of the Contemplation Ladder and URICA scales. *Drug and alcohol dependence.* 2004;73(3):301-306.

44. Beebe J. Rapid assessment process. In: Kempf-Leonard K, ed. *The encyclopedia of social measurement*2005:285-291.

45. Edelman EJ, Hansen NB, Cutter CJ, et al. Implementation of integrated stepped care for unhealthy alcohol use in HIV clinics. *Addiction science & clinical practice.* 2016;11(1):1.

46. Edelman EJ, Cole CA, Richardson W, Boshnack N, Jenkins H, Rosenthal MS. Opportunities for improving partner notification for HIV: results from a community-based participatory research study. *AIDS and behavior.* 2014;18(10):1888-1897.

47. Camenga DR, Hieftje KD, Fiellin LE, Edelman EJ, Rosenthal MS, Duncan LR. The use of message framing to promote sexual risk reduction in young adolescents: a pilot exploratory study. *Health education research.* 2014;29(2):360-366.

48. Hieftje K, Rosenthal MS, Camenga DR, Edelman EJ, Fiellin LE. A qualitative study to inform the development of a video game for adolescent HIV prevention. *Games for health journal.* 2012;1(4):294-298.

49. Hsieh HF, Shannon SE. Three approaches to qualitative content analysis. *Qualitative health research.* 2005;15(9):1277-1288.

50. Lee JD, Vocci F, Fiellin DA. Unobserved "home" induction onto buprenorphine. *Journal of addiction medicine.* 2014;8(5):299-308.

51. Kirchner JE, Parker LE, Bonner LM, Fickel JJ, Yano EM, Ritchie MJ. Roles of managers, frontline staff and local champions, in implementing quality improvement: stakeholders' perspectives. *Journal of evaluation in clinical practice.* 2012;18(1):63-69.

52. May CR, Mair F, Finch T, et al. Development of a theory of implementation and integration: Normalization Process Theory. *Implementation science : IS.* 2009;4:29.

53. Egan JE, Casadonte P, Gartenmann T, et al. The Physician Clinical Support System-Buprenorphine (PCSS-B): a novel project to expand/improve buprenorphine treatment. *Journal of general internal medicine.* 2010;25(9):936-941.

54. Magerko B, Deen J, Idnani A, Pantalon MV, D'Onofrio G. Dr. Vicky: A Virtual Coach for Learning Brief Negotiated Interview Techniques for Treating Emergency Room Patients. *AAAI Spring Symposium: AI and Health Communication.* 2011;SS11-1:25-32.

55. D'Onofrio G, Pantalon MV, Degutis LC, Fiellin DA, O'Connor P G. Development and implementation of an emergency practitioner-performed brief intervention for hazardous

COPY

and harmful drinkers in the emergency department. *Academic emergency medicine : official journal of the Society for Academic Emergency Medicine.* 2005;12(3):249-256.

56. D'Onofrio G, Fiellin DA, Pantalon MV, et al. A brief intervention reduces hazardous and harmful drinking in emergency department patients. *Annals of emergency medicine.* 2012;60(2):181-192.

57. Parzen M. A random effects model for simulating clustered binary data. *Submitted to Statistics and Computing.* 2016.

58. Datta S, Satten GA. A signed-rank test for clustered data. *Biometrics.* 2008;64(2):501-507.

59. French MT, Popovici I, Tapsell L. The economic costs of substance abuse treatment: updated estimates and cost bands for program assessment and reimbursement. *Journal of substance abuse treatment.* 2008;35(4):462-469.

COPY
