# Supplementary material for: Implementation Facilitation to Promote Emergency Department–Initiated Buprenorphine for Opioid Use Disorder
Source: JAMA Netw Open. 2023 Apr 5;6(4):e235439. doi: 10.1001/jamanetworkopen.2023.5439 (PMC10077107; doi:10.1001/jamanetworkopen.2023.5439)
Supplement: Supplement 2. — Amended Trial Protocol and Statistical Analysis Plan [file jamanetwopen-e235439-s002.pdf]

Statistical Analysis Plan for NIDA Protocol CTN-0069

Opioid Use Disorder in the Emergency Department (ED-HEALTH)

Lead Investigator: Gail D'Onofrio, MD, MS

Co-Lead Investigator: David A. Fiellin, MD

Version 2.0

September 20, 2021

Prepared by:

NIDA CTN Data and Statistics Center

CONFIDENTIAL

SIGNATURE PAGE

Lead Investigator: Gail D'Onofrio, MD, MS

Signature: ______________________________ Date: ____________

Co-Lead Investigator: David A. Fiellin, MD

Signature: ______________________________ Date: ____________

CCTN Scientific Officer: Kristen Huntley, PhD

Signature: ______________________________ Date: ____________

DSC Lead Statistician: Amy Hahn, MS

Signature: ______________________________ Date: ____________

DSC Leadership: Abigail Matthews, PhD

Signature: ______________________________ Date: ____________

TABLE OF CONTENTS

LIST OF ABBREVIATIONS vii
LIST OF PATIENT PARTICIPANT eCRFs viii
LIST OF PROVIDER PARTICIPANT eCRFs ix
1.0 INTRODUCTION 10
2.0 SUMMARY OF STUDY DESIGN AND PROCEDURES 10
2.1 Study Objective 10
2.2 Study Design 10
2.3 Study Procedures 11
2.3.1 Implementation Facilitation 11
2.3.1.1 Overview of the Implementation Facilitation 11
2.3.1.2 Quantitative Data Collection and Analysis 11
2.3.1.3 Qualitative Data Collection and Analysis 11
2.3.1.4 Administration of BUP During IF Evaluation Period 12
2.3.1.5 EHR-Abstracted Data about MOUD Activities 12
2.3.2 Patient Participants 12
2.3.2.1 Overview of Patient Participants 12
2.3.2.2 Induction onto BUP 12
2.3.2.3 Qualitative Data Collection and Analysis 13
2.3.3 Randomization and Blinding 13
2.4 Eligibility Criteria for Selection of Both Study Populations 13
2.4.1 Inclusion Criteria 13
2.4.1.1 IF ED Provider Participants 13
2.4.1.2 Community Opioid Treatment Provider Participants 14
2.4.1.3 Patient Participants 14
2.4.1.4 Focus Group Participants 14
2.4.2 Exclusion Criteria 14
2.4.2.1 Patient Participants 14
3.0 GENERAL ANALYSIS DEFINITIONS AND CONVENTIONS 15
3.1 Patient Participant Analysis Populations 15
3.1.1 Pre-screened Population 15
3.1.2 Screened Population 15
3.1.3 Intent-to-Treat Population 15
3.1.4 Safety Population 15
3.2 Other Populations 15
3.2.1 Survey Participant Population 15
3.2.2 Focus Group Participant Population 15
3.3 General Definitions 15
3.3.1 Index ED Visit 15
3.3.2 Follow-up Visit 15
3.3.3 IF Survey Dates 15

3.3.4 Study Day 16
3.3.5 Safety Window 16
3.3.6 Calendar Time 16
3.4 Table, Figures and Listings Conventions 16
4.0 PARTICIPANT ENROLLMENT, DISPOSITION, AND FOLLOW-UP VISIT ATTENDANCE 17
4.1 Participant Enrollment 17
4.1.1 Patient Participant Enrollment 17
4.2 Participant Disposition 17
4.2.1 Patient Participant Disposition 17
4.2.2 Provider Participant Disposition 17
4.2.3 Focus Group Disposition 17
4.3 Follow-up Visit Attendance 17
5.0 ANALYSIS OF PARTICIPANT BASELINE CHARACTERISTICS 18
5.1 Patient Participant Baseline Characteristics 18
5.2 Provider Participant Characteristics 18
5.3 Focus Group Participant Characteristics 18
6.0 STUDY INTERVENTION ADHERENCE 18
7.0 IMPLEMENTATION AND EFFECTIVENESS ANALYSIS 18
7.1 Definition of Primary Implementation Outcome Measure 18
7.2 Analysis of the Primary Implementation Outcome Measure 19
7.2.1 ChangeToOne Policy versus the Bayesian Approach 20
7.3 Definition of Primary Effectiveness Outcome Measure 21
7.4 Analysis of Primary Effectiveness Outcome Measure 21
7.5 Supportive Analyses of the Primary Outcome Measures 22
7.5.1 Subgroup Analyses 22
7.5.2 Sensitivity Analyses 22
7.5.3 Missing Data 23
7.5.4 Secondary Analyses Related to the Primary Effectiveness Outcome 23
7.6 Definition of Secondary Outcome Measures 23
7.6.1 Secondary Implementation Outcomes 23
7.6.2 Secondary Effectiveness Outcomes 24
7.7 Exploratory Analyses 24
8.0 SAFETY ANALYSIS 26
8.1 ED Visits and Hospitalizations 26
8.2 Overdose Events 26
8.3 Suicide Risk 26
8.4 Deaths 26
9.0 SIGNIFICANCE TESTING AND MULTIPLICITY 27
10.0 SAMPLE SIZE AND POWER 27
10.1 ChangeToOne Policy 27

10.1.1 Bayesian Approach 28
10.2 Summary of Power Simulations 28
11.0 INTERIM ANALYSES AND DATA MONITORING 28
12.0 DATA QUALITY 29
12.1 Data Audits 29
12.2 Protocol Deviations 29
13.0 SOFTWARE TO BE USED FOR ANALYSES 29
14.0 UPDATES TO THE STATISTICAL ANALYSIS PLAN 29
15.0 LIST OF PROPOSED TABLES, FIGURES, AND LISTINGS 30
16.0 SHELLS FOR PROPOSED TABLES, FIGURES AND LISTINGS 34
16.1 Patient Participant Data 34
16.1.1 Enrollment, Disposition and Follow-up 34
16.1.2 Baseline Characteristics 47
16.1.3 Primary Implementation Outcome 63
16.1.4 Primary Effectiveness Outcome 69
16.1.5 Safety 77
16.1.6 Data Quality 90
16.1.7 Focus Group Participant Characteristics 95
17.0 REFERENCES 113
18.0 APPENDIX: BAYESIAN ANALYSIS 114
18.1 Figure B5: Bayes power; Alternative Cases 114
18.2 Figure B6: Bayes Credibility Intervals for P1, P2; Alternative Cases 115
18.3 Figure B7: Bayes Credibility Intervals for P1-P2; Alternative Cases 115
18.4 Figure B8: Bayes Test Size; Null Cases 116
18.5 Figure B9: Bayes Credibility Intervals for P1, P2; Null Cases 116
18.6 Figure B10: Bayes Credibility Intervals P1-P2; Null Cases 117

FIGURES

Figure 1A: Patient Participants Flow Diagram – Baseline Evaluation Period 34
Figure 2: Proposed versus Actual Enrollments for Baseline Evaluation Period by Site 40
Figure 3: Proposed versus Actual Enrollments for IF Evaluation Period by Site 41

TABLES

Table 1: Summary of Screen Failures by Evaluation Period 35
Table 2: Summary of Screen Failures by Site 37
Table 3: Summary of Enrollment by Evaluation Period 39
Table 4: Summary of Enrollment by Site 39
Table 5: Summary of Patient Participant Disposition by Evaluation Period 42
Table 6: Summary of Patient Participant Disposition by Site 43
Table 7: Summary of Attendance at Follow-up Visit by Evaluation Period 44
Table 8: Summary of Attendance at Follow-up Visit by Site 44
Table 9: Summary of Enrolled Patient Participants Who Were Ineligible by Evaluation Period ..45

Table 10: Summary of Enrolled Patient Participants Who Were Ineligible by Site 46
Table 11: Summary of Patient Participant Baseline Characteristics by Evaluation Period 47
Table 12: Summary of Patient Participant Baseline Characteristics by Site..............................55
Table 13: Summary of Primary Implementation Outcome by Evaluation Period........................63
Table 14: Summary of Primary Implementation Outcome by Site and Evaluation Period 64
Table 15: Analysis Results for Primary Implementation Outcome 64
Table 16: Summary of Primary Implementation Outcome by Sex and Evaluation Period 65
Table 17: Summary of Primary Implementation Outcome by Race and Evaluation Period 65
Table 18: Summary of Primary Implementation Outcome by Ethnicity and Evaluation Period...66
Table 19: Final Covariate Adjusted Model Analysis Results for Primary Implementation Outcome 67
Table 20: Summary of MOUD Practices by Site Over Time from EHR-abstracted Data............68
Table 21: COVID-19 Sensitivity Analyses Results for Primary Implementation Outcome 69
Table 22: Summary of Primary Effectiveness Outcome by Evaluation Period 69
Table 23: Summary of Primary Effectiveness Outcome by Site and Evaluation Period 70
Table 24: Analysis Results for Primary Effectiveness Outcome 71
Table 25: Summary of Primary Effectiveness Outcome by Sex and Evaluation Period 72
Table 26: Summary of Primary Effectiveness Outcome by Race and Evaluation Period 72
Table 27: Summary of Primary Effectiveness Outcome by Ethnicity and Evaluation Period......73
Table 28: Final Covariate Adjusted Model Analysis Results for Primary Effectiveness Outcome 74
Table 29: COVID-19 Sensitivity Analyses Results for Primary Effectiveness Outcome 75
Table 30: Summary of Treatment Engagement at Day 30 during the IF Evaluation Period by ED-initiated BUP Status: All Patient-participants (Missing Engagement Status Imputed as Not Engaged) 76
Table 31: Summary of Treatment Engagement at Day 30 during the IF Evaluation Period by ED-initiated BUP Status: Patient-participants with Non-missing Engagement Status (No Imputation) 76
Table 32: Analysis of Treatment Engagement at Day 30 during the IF Evaluation Period by ED-initiated BUP Status 76
Table 33: Summary of Hospitalizations Post Index Visit by Evaluation Period 77
Table 34: Summary of ED Visits Post Index Visit by Evaluation Period....................................80
Table 35: Summary of Overdoses by Evaluation Period 83
Table 36: Summary of Suicide Risk by Evaluation Period 86
Table 37: Summary of Data Audits by Site...90
Table 38: Summary of Protocol Deviations by Site..91
Table 39: Summary of Provider Participant Characteristics by Site 93
Table 40A: Summary of Focus Groups Participant Characteristics: Wave 1 95
Table 41A: Summary of Focus Groups Participant Characteristics: Wave 2 101
Table 42A: Summary of Focus Groups Participant Characteristics: Wave 3 107

LISTINGS

Listing 1A: Listing of Hospitalizations Post Index Visit by Evaluation Period.............................78
Listing 2: Listing of Hospitalizations Post Index Visit for Ineligible Participants..........................79
Listing 3A: Listing of ED Visits Post Index Visit by Evaluation Period.......................................81
Listing 4: Listing of ED Visits Post Index Visit for Ineligible Participants 82
Listing 5A: Listing of Overdoses by Evaluation Period...84
Listing 6: Listing of Overdoses for Enrolled Patient Participants that Were Ineligible.................85
Listing 7A: Listing of Suicide Risk by Evaluation Period 87

Listing 8: Listing of Suicide Risk for Enrolled Patient Participants who Were Ineligible..............88
Listing 9: Listing of Deaths by Evaluation Period[1]..89
Listing 10: Listing of Protocol Deviations by Site 92

CONFIDENTIAL

LIST OF ABBREVIATIONS

Abbreviation	Definition
AE	Adverse Event
BEP	Baseline Evaluation Period
BUP	Buprenorphine or buprenorphine/naloxone
CAC	Critical Action Checklist
CRF	Case Report Form
CTN	Clinical Trials Network
CEA	Cost Effectiveness Analyses
DSC	Data and Statistics Center
DSM-5	Diagnostic and Statistical Manual of Mental Disorders, Fifth Edition
DATA	Drug Addiction Treatment Act
eCRF	Electronic Case Report Form
ED	Emergency Department
EHR	Electronic Health Record
EMR	Electronic Medical Record
FSR	Final Study Report
IF	Implementation Facilitation
IFEP	Implementation Facilitation Evaluation Period
ICC	Intra Class Correlation
LN	Lead Node
MAT	Medication-assisted Treatment
NA	Narcotics Anonymous
NIDA	National Institute on Drug Abuse
ORCA	Organizational Readiness to Change Assessment
OUD	Opioid Use Disorder
OTP	Opioid Treatment Program
PARiHS	Promoting Action on Research Implementation in Health Services
PI	Principal Investigator
RA	Research Associate
SAE	Serious Adverse Event
TLFB	Timeline Follow-back
UDS	Urine Drug Screen

LIST OF PATIENT PARTICIPANT eCRFs

Form Code	Form Name
AD1	Adverse Event
AD2	Serious Adverse Event Summary
AD3	Serious Adverse Event Medical Reviewer
CAC	Critical Action Checklist
CCJ	Crime and Criminal Justice
CLG	Contact Log
COM	Communication Log
DEM	Demographics
DM1	Additional Demographics
EC0069B	Enrollment 0069B (Baseline Evaluation)
EC0069C	Enrollment 0069C (IF Evaluation Period)
EC0069P	Enrollment 0069P (Screening)
EDR	ED Visit Review
EDV	ED Visits and Hospitalization
EHQ	ED Health Quiz
EQD	EQ-5D-3L
ESL	ED Daily Screening Log
ETF	Engagement in Treatment: Facility
ETP	Engagement in Treatment: Patient
F69	Timeline Followback Page 2
FLG	Facility Contact Log
FVT	Follow-up Visit Scheduling
HS1	Health Services Utilization Page 1: Inpatient
HS2	Health Services Utilization Page 1: Outpatient
HSI	Health Services Utilization: Inpatient
HSO	Health Services Utilization: Outpatient
HST	Health Status
HVL	Healthcare Visit Logistics
MVF	Missed Visit
NDI	National Death Index

Form Code	Form Name
ODE	Overdose Events
OSU	Other Substance Use
PDR	Protocol Deviation Review
PDV	Protocol Deviation
PES	Patient Eligibility Summary
STC	Study Completion
T69	Timeline Followback Page 1
TAP	TLFB Assessment Period
UDS	Urine Drug Screen

LIST OF PROVIDER PARTICIPANT eCRFs

Form Code	Form Name
D69	Focus Group Demographics
EC0069IB	Enrollment 0069IB (Provider Participant)
FGT	Focus Group Tracking
ICH	Individual Characteristics
OC1	Organizational Readiness to Change Assessment (ORCA) Community
OC2	Organizational Readiness to change Assessment (ORCA+) Community
OE1	Organizational Readiness to Change Assessment (ORCA) ED
OE2	Organizational Readiness to change Assessment (ORCA+) ED
RRL	Readiness Ruler
SC1	Site Characteristics - ED
SC2	Site Characteristics – OTP Page 1
SC3	Site Characteristics – OTP Page 2
SC4	Site Characteristics – OTP Page 3
SC5	Site Characteristics – Community Page 1
SC6	Site Characteristics – Community Page 2
WEL	Introduction

1.0 INTRODUCTION

The Statistical Analysis Plan (SAP) for CTN-0069 “Opioid Use Disorder in the Emergency Department (ED-HEALTH)” expands upon the statistical information presented in the protocol and describes planned analyses for the primary, secondary, exploratory and safety outcome measures. The CTN’s DSC will conduct the analyses for the FSR as listed in Table 1 below.

Table 1: Analysis Responsibilities

Content	Section Number	Responsible for Analysis
Patient Participant Enrollment, Disposition, and Follow-up	4.0	DSC
Patient, Survey, and Focus Group Participant Baseline Characteristics	5.0	DSC
Analyses of Primary Implementation and Effectiveness Outcomes	7.5	DSC
Summaries of Secondary Outcome Measures	7.6	LN
Analyses of the Secondary Outcome Measures	7.6	LN
Analyses of the Exploratory Outcome Measures	7.7	LN
Cost Effectiveness Analysis	7.7	Columbia
Safety Summaries	8.0	DSC
Data Quality	12.0	DSC

2.0 SUMMARY OF STUDY DESIGN AND PROCEDURES

2.1 Study Objective

The objective of the study is to evaluate the impact of (1) Implementation Facilitation (IF) on rates of provision of Emergency Department (ED)-initiated buprenorphine/naloxone (BUP) treatment with referral for ongoing medication-assisted treatment (MAT) and the (2) effectiveness of IF on patient engagement in formal addiction treatment for OUD at 30 days post-enrollment.

2.2 Study Design

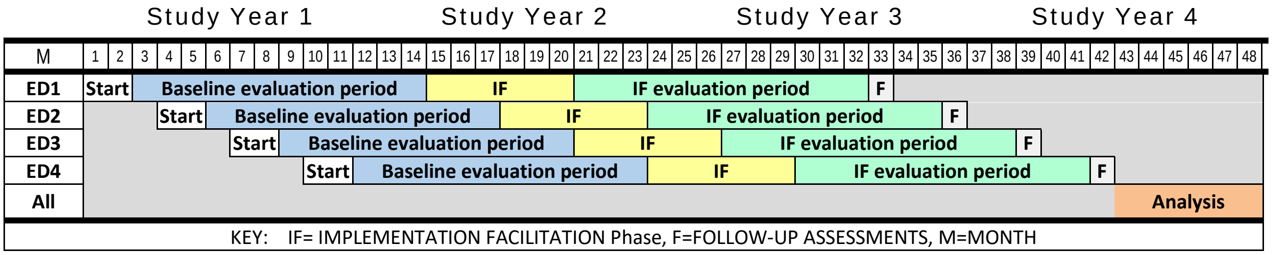

This study uses a Hybrid Type 3 Effectiveness-Implementation framework and a modified stepped wedge design. In a Hybrid Type 3 Effectiveness-Implementation study the primary research

question is the implementation strategy's impact on uptake. In addition, the Hybrid Type 3 design allows an assessment of the implementation strategy's impact on related effectiveness outcomes.

The study will be conducted at four ED study sites with a high prevalence of patients with untreated opioid use disorder (OUD), an existing research infrastructure and a potential network of community opioid treatment providers and programs. The study populations will include:

1. ED providers and staff involved in the treatment of patients with OUD;
2. Community opioid treatment providers and program staff involved in providing care for patients with OUD referred from the ED;
3. Approximately 960 ED patients with moderate to severe OUD.

Participants will be enrolled either during the Baseline Evaluation Period (BEP) or the IF Evaluation Period (IFEP). The IFEP is preceded by a period of 6 months of IF at each of the sites.

2.3 Study Procedures

Study procedures are divided into Implementation Facilitation (Section 2.3.1) and patient participants (Section 2.3.2).

2.3.1 Implementation Facilitation

2.3.1.1 Overview of the Implementation Facilitation

Building on the mixed-methods analysis conducted during the formative evaluation, the study will use the Promoting Action on Research Implementation in Health Services (PARiHS) framework to tailor the IF for site-specific needs. The facilitators and barriers identified by administrators, providers, and patients will be characterized according to the PARiHS sub-elements of patient and clinical experience (communication, knowledgeable and empathetic providers), receptive context (resources to provide addiction treatments), and culture (value of team-based approach) identified. PARiHS will be used to further explicate and design the IF, guide the ongoing formative evaluation, and revise the strategy in an iterative manner to improve implementation success. Other components of IF include external facilitators, local champions, provider education and academic detailing, stakeholder engagement, tailoring the program to local sites, performance monitoring and feedback, formative evaluation learning collaborative, and program marketing.

2.3.1.2 Quantitative Data Collection and Analysis

Baseline Organizational Readiness to Change Assessment (ORCA) and provider readiness and preparedness scores will be used to determine evidence and context related strengths and weaknesses in organizational readiness to implement BUP and referral and to tailor the IF. Other process measurements will include the number of ED providers attending the initial educational session, participation in the BUP training courses, acquisition of a DATA 2000 waiver, proportion of eligible patients receiving ED-initiated BUP, provider skill and adherence to critical actions on initiating BUP treatment with referral to ongoing MAT. Additional process measurements with organizational data will include integration of materials into EHR and proportion of patients with ED-initiated BUP who are successfully linked to office-based BUP providers and/or Opioid Treatment Programs (OTPs).

2.3.1.3 Qualitative Data Collection and Analysis

At each of the four ED study sites, the study will conduct focus groups with a purposeful sample of key stakeholders at multiple distinct stages of the project: during the first month of the IF period, at approximately the fourth or fifth months of the IF period, and nearing the completion of the IFEP. However, data collected during focus groups and during the formative evaluation is part of an iterative process, and therefore additional focus groups, one-on-one phone interviews, and

email correspondence will take place as needed. The study will enroll a variety of participants including ED patients, nurses, social workers, physicians, nurse practitioners, physician assistants, pharmacists, and physician and nursing directors at each ED site and office-based BUP providers, as well as representatives from OTPs to allow for evaluation of processes from multiple perspectives (triangulation). Focus groups will be conducted with approximately four to eight study participants and representation from each of the stakeholder categories.[1]

2.3.1.4 Administration of BUP During IF Evaluation Period

The study will assess fidelity to the procedures (adherence) using a critical action checklist. The checklist will include confirmation of documentation of: urine toxicology and liver function tests obtained; patient participant meeting criteria for DSM-5 moderate-severe opioid use disorder; urine positive for opioid; formal assessment of Clinical Opioid Withdrawal Scale (COWS); ED-initiated BUP provided; BUP education and induction instructions provided; and referral for ongoing medication-assisted treatment (MAT).

2.3.1.5 EHR-Abstracted Data about MOUD Activities

Sites will review their electronic health records (EHR) to capture and abstract the following at various timepoints: (1) BUP prescribing/administration; (2) BUP waivers amongst ED staff; (3) naloxone prescribing/dispensing; and (4) providers prescribing naloxone. This data was provided directly to the Lead Node, who compiled into a single dataset and provided to the DSC for inclusion in the official study dataset.

2.3.2 Patient Participants

2.3.2.1 Overview of Patient Participants

Patients will be recruited throughout the entire evaluation periods at each ED site. Research Associates (RAs) assigned to the study will work in shifts to ensure cross coverage to screen all ED patients who are potentially eligible for the study. The RA will use the ED log to identify all patients seen in the ED and will eliminate patients with obvious exclusions such as under police custody. Patients will be asked for verbal consent to complete a set of screening assessments starting with a screener that includes questions about illicit opioid use in the past 30 days embedded in a general health and substance use screener that also includes questions about safety and tobacco and alcohol use.[2,3] The screener will contain questions regarding heroin/fentanyl and non-medical use of prescription opioids. Potential study patients who report any opioid use in the past month will complete a seven-day Time-line Follow Back (TLFB)[4] method. If opioid use is reported during the past seven days, a brief (10 minute) structured diagnostic interview (DSM-5) to evaluate for the presence of moderate/severe OUD is administered. Those who meet criteria for moderate/severe OUD will be informed that they may qualify for a study if they are willing to produce a urine sample. Patients will be offered participation and written informed consent will be obtained if the urine tests are positive for any opioid (*fentanyl <u>only</u> are not eligible due to lack of CLIA-waived point of care testing*), the patient indicates he/she is able to provide contact information for two reliable contacts, and the patient meets all eligibility criteria on the Patient Participant Eligibility Summary form.

2.3.2.2 Induction onto BUP

Buprenorphine induction will take place in the ED or unobserved and should be based on the study patient’s level of opioid withdrawal as measured by the COWS. Study patients will receive a dose in the ED if they exhibit moderate to severe withdrawal on the COWS and will leave with a prescription for the daily doses needed prior to their scheduled follow-up appointment. In an effort to maximize retention and abstinence achievement during the induction the investigators

will instruct ED providers to provide study patients with a scheduled appointment for follow up with an office-based BUP provider or an OTP within 96 hours of their ED visit.

2.3.2.3 Qualitative Data Collection and Analysis

Patient participant baseline data will include a brief instrument assessing health status, healthcare utilization, overdose events, past seven-day alcohol and drug use including opioids using the TLFB method, use of other substances, the EuroQol (EQ-5D), and other cost. Assessments collected at 30 days post-study enrollment will be similar. In addition, at 30 days post-enrollment, participants will be asked to report OUD treatment received on their 30th day post enrollment target date.

2.3.3 Randomization and Blinding

Randomization does not occur at participant level. Five months before the start of a new step in the stepped wedge design, one site from those still in the BEP will be randomly selected to switch over to the IF period. The DSC performed this randomization and notified the LN via email of the site selected.

In an attempt to institute blinding and conduct an accurate BEP, ED provider participants, other than the site PI, were not notified of the intent of the study or the plan for an IF or IFEP. Patient participants were not notified of the intent of the study with respect to IF or IFEP. Research Assistants were notified of the intent of the IF and IFEP as each site transitioned to IF.

2.4 Eligibility Criteria for Selection of Both Study Populations

2.4.1 Inclusion Criteria

2.4.1.1 IF ED Provider Participants

ED provider participants must be credentialed to practice in the site ED and capable of prescribing BUP. This includes physicians, nurses, nurse practitioners, and physician assistants. Participating EDs should have the following characteristics and respond to the Data Call (site recruitment survey) regarding:

1. Large prevalence of patients with untreated OUD so that the target of 10-12 patients into the study per month can be met. Sites must provide number of ICD 9/10 codes for the past year related to overdose and opioid dependence, abuse, and unspecified use/opioid use disorders
2. An electronic health record that can be queried daily to weekly
3. Wireless internet access as information entered on tablets will be uploaded to a secure study server
4. Prior clinical research experience (report funded projects, enrollment, retention, etc.)
5. An Emergency Physician with experience as Principal Investigator (PI) and with time to devote to the project
6. No current routine use of ED-initiated BUP
7. Ability to have BUP on their formulary and available to the ED
8. Ability to present a plan for patient flow and space utilization
9. Have or able to hire appropriate staff to conduct the study
10. Have sufficient referral network for patients needing MAT that could potentially accommodate referrals in 96 hours

11. Be in a state that allows for MAT through its Medicaid program
12. Have accessible pharmacies to fill BUP prescriptions
13. Have an active state prescription monitoring program

2.4.1.2 Community Opioid Treatment Provider Participants

1. At least one office-based provider of BUP not currently at their limit according to DATA 2000 provisions, and one OTP without active waiting lists
2. Programs and/or providers with the ability to accept patients with a variety of insurance plans (including Medicaid) within 96 hours of ED-initiated BUP
3. Treatment providers and/or programs located within the general vicinity of where ED patients reside

2.4.1.3 Patient Participants

1. Be 18 years or older
2. Treated in the ED during study screening hours
3. Meet DSM-5 diagnostic criteria for moderate to severe opioid use disorder
4. Have a urine toxicology test that is positive for opioids (opiates, oxycodone, buprenorphine, or methadone). For patients with acute pain conditions requiring opioid administration, urine will need to be obtained prior to ED opioid medication administration.

2.4.1.4 Focus Group Participants

Providers, staff, and patients in EDs, office-based practices, and community-based programs will be included who are:

1. 18 years or older
2. Able to provide verbal informed consent

ED patients will include those previously enrolled during either Evaluation Period.

2.4.2 Exclusion Criteria

2.4.2.1 Patient Participants

1. Have a medical or psychiatric condition that requires hospitalization at the index ED visit
2. Be actively suicidal or severely cognitively impaired precluding informed consent
3. Present from extended care facility (e.g., skilled nursing facility)
4. Require continued prescription opioids for a pain condition
5. Be a prisoner or in police custody at the time of index ED visit
6. Currently have (past 30 days) been enrolled in formal addiction treatment for OUD, including by court order
7. Inability to provide reliable locator information including two contact numbers
8. Be unwilling to follow study procedures (e.g., unwilling to provide permission to contact referral provider/program or return for 30-day assessment)
9. Have prior enrollment in the current study. Note: A patient may **NOT** enroll in both the BEP and IFEP.

10. Not able to speak English sufficiently to understand the study procedures and provide written informed consent to participate in the study

3.0 GENERAL ANALYSIS DEFINITIONS AND CONVENTIONS

3.1 Patient Participant Analysis Populations

3.1.1 Pre-screened Population

The pre-screened population consists of all patient participants who present in the ED during screening hours.

3.1.2 Screened Population

The screened population consists of all patient participants who provided verbal consent at the initiation of the screening process.

3.1.3 Intent-to-Treat Population

The intent-to-treat (ITT) population consists of ED patients who provided informed consent and enrolled in the study. Data from ITT population will be used to evaluate the primary implementation outcome, primary effectiveness outcome, secondary effectiveness outcomes, and rates of enrolled patient participants receiving an appointment for opioid treatment provider/program upon ED discharge as part of secondary implementation outcome measures. Note that participants who enrolled in the study but were subsequently determined to have been ineligible are *not* included in the ITT population.

3.1.4 Safety Population

The safety population includes all patient participants who provided informed consent during the screening visit. This includes enrolled patient participants that were later found to be ineligible.

3.2 Other Populations

3.2.1 Survey Participant Population

The survey population includes all provider participants who were enrolled in the study and who completed at least one IF survey.

3.2.2 Focus Group Participant Population

The focus groups population includes all individuals who participated in at least one focus group.

3.3 General Definitions

3.3.1 Index ED Visit

The Index ED Visit is the first visit for patient participants and involves both screening and enrollment for each evaluation period.

3.3.2 Follow-up Visit

The Follow-up visit date is defined as the 30th day estimated post-index ED Visit discharge. The actual visit can occur later, but information is only collected about events that occurred within 30 days of the index ED visit discharge date.

3.3.3 IF Survey Dates

The baseline survey will occur toward the end of the BEP. This will be followed by two more survey visits (Follow-up Survey 1 and Follow-up Survey 2) that will occur at the beginning and toward the end of the IFEP.

3.3.4 Study Day

Study Day is defined as the number of days post-index ED Visit with Study Day 0 defined as the date eligibility confirmed which is collected on the enrollment forms for segments B and C (i.e., EC0069B, EC0069C).

3.3.5 Safety Window

As buprenorphine is an FDA approved marketed medication with known and labeled adverse events, safety events are only recorded on the AE/SAE form within the 30-day follow-up period and not followed to resolution. The safety window for this study is, generally, from 30 days prior to the index ED visit to Study Day 30. For ED visits and hospitalizations, suicidal ideation, and overdoses, the window is 30 days prior to the index ED visit for baseline and from baseline to Study Day 30 for the follow-up visit. Deaths occurring prior to Day 30, whether the site identifies on their own or the information was provided by NDI, are included. Should the site learn of a death (aside from NDI) after Study Day 30 and before the follow-up visit occurred, then this will be reported on the STC.

3.3.6 Calendar Time

Calendar time for analysis is defined as the number of days since the site opened for enrollment in the baseline evaluation period. Below are the start and end dates for each site in each evaluation period. The start date is defined as the date the site was given access to the eClinical system to begin enrolling participants in the study for that particular evaluation period. The end date is defined as the last day the site was able to enroll participants in the EDC system for each evaluation period. Note that during the IFEP, enrollment was paused due to the COVID-19 public health measures put into place at two sites, Harborview Medical Center and University of Cincinnati. The days enrollment was paused will be considered as part of the primary analysis and several sensitivity analyses will be performed to account for this pause.

	Baseline Evaluation Period		IF Evaluation Period			
Site	Start Date	End Date	Start Date	Date Paused for COVID-19	Date Reopened for COVID-19	End Date
MA Johns Hopkins ED	4/10/2017	4/9/2018	10/9/2018	N/A	N/A	10/8/2019
GNY Mount Sinai ED/Beth Israel	7/6/2017	7/5/2018	1/4/2019	N/A	N/A	1/5/2020
OV University of Cincinnati ED	10/9/2017	10/8/2018	4/8/2019	03/13/2020	6/10/2020	7/6/2020
PNW Harborview Medical Center ED	1/2/2018	1/1/2019	7/2/2019	03/3/2020	6/6/2020	10/4/2020

3.4 Table, Figures and Listings Conventions

All summary analyses described in this document for patient participants will be summarized by evaluation period and/or by site. Focus group data will be summarized by site for each individual wave (first month of IF, four to five months of IF, and near completion of IF period) and overall (the three waves combined) for the following categories: 1) patients, 2) providers and 3) all focus group participants combined. For IF provider surveys, respondent characteristics, Readiness

Ruler scores, and ORCA scores will be summarized by site, assessment period (Baseline, Follow-up 1 and Follow-up 2), and provider type (i.e., ED versus community provider). For all populations, descriptive statistics for continuous variables will be presented with mean, standard deviation, minimum, 25th percentile, median, 75th percentile, and maximum. Categorical variables will be summarized in terms of percentages and frequencies.

4.0 PARTICIPANT ENROLLMENT, DISPOSITION, AND FOLLOW-UP VISIT ATTENDANCE

4.1 Participant Enrollment

4.1.1 Patient Participant Enrollment

Proposed versus actual patient participant enrollments, under the assumption that 10 patient participants were expected to be enrolled per month per site, will be summarized by site and evaluation period in a tabular fashion and graphically. A flow diagram of patient participants will be presented that includes information on ineligibility and loss to follow-up for each evaluation period. Number of screen failures and reason for failure will be summarized by site and evaluation period as well. Number of ineligible patient participants that were enrolled will also be summarized by site and evaluation period.

4.2 Participant Disposition

4.2.1 Patient Participant Disposition

The number of patient participants who terminated early from the study along with the reasons for early termination will be summarized by site. Patient participants are defined as study completers if the Day 30 follow-up visit is completed as noted on the Study Completion (STC) form (i.e., STCOMPLT='1' or STLTEFUP='1'); they are considered non-completers if the visit is not completed (i.e., STCOMPLT='0' and STLTEFUP='0'). Patient participant disposition will be summarized by evaluation period for the number of patient participants completing the study, number of patient participants who completed the study within the window for the Day 30 follow-visit, the number of patient participants non-completed from the study, and the reasons for non-completion.

4.2.2 Provider Participant Disposition

The number of survey participants who completed each survey will be summarized by site. Survey participants are defined as completers if they completed the Site Characteristics form; they are considered non-completers if the form is not completed. Survey participants from an ED must complete the Site Characteristics-ED (SC1) form, survey participants from an OTP must complete the Site Characteristics – OTP Page 3 (SC4) form, and survey participants from a Community site must complete the Site Characteristics – Community Page 2 (SC6) form.

4.2.3 Focus Group Disposition

The number of focus group participants who completed each wave will be summarized by site.

4.3 Follow-up Visit Attendance

Attendance at the 30-day follow-up visit will be summarized by presenting the number and percentage of participants who attended by site and evaluation period. The visit is considered attended if the Engagement in Treatment: Participant form is completed.

5.0 ANALYSIS OF PARTICIPANT BASELINE CHARACTERISTICS

5.1 Patient Participant Baseline Characteristics

Demographics and characteristics such as sex, age, ethnicity, race, education completed, marital status, employment, timeline follow-back self-reported substance use, urine drug screen (including buprenorphine and fentanyl positivity), whether index ED visit was opioid-related, overdose events and EQ-5D-3L (i.e., EuroQol-5D) will be summarized by site and evaluation period. Since it is expected that participants with similar demographic characteristics will be enrolled during both evaluation periods, statistical comparisons of evaluation periods with respect to characteristics will be informal. If differences between evaluation periods are suspected, statistical testing will be performed.

5.2 Provider Participant Characteristics

Survey respondent characteristics for participants who started the Site Characteristics will also be summarized by site and time period (Baseline Survey, Follow-up Survey 1, Follow-up Survey 2), and provider type (ED and community).

5.3 Focus Group Participant Characteristics

Focus group participants and their characteristics (age, sex, race, ethnicity, education level, employment status, job title and marital status) will be summarized by site for each wave (first month of IF, four to five months of IF, and near completion of IF period for the following categories: (1) patients, (2) providers and (3) all focus group participants combined.

6.0 STUDY INTERVENTION ADHERENCE

Several of the secondary implementation outcomes capture adherence to the study intervention (i.e., IF). See Section 7.6.1 for a list of all secondary implementation outcomes.

7.0 IMPLEMENTATION AND EFFECTIVENESS ANALYSIS

7.1 Definition of Primary Implementation Outcome Measure

The primary outcome is the rate of provision of ED-initiated BUP with referral for ongoing MAT. The rates of provision of ED-initiated BUP with referral for ongoing MAT will be based on:

1. Proportion of enrolled patients who receive ED-initiated BUP with referral for ongoing MAT or
2. Computed from ED data on the numbers of providers and their patients who received ED-initiated BUP with referral for ongoing MAT. As estimating a very low frequency outcome by sampling is not reliable, computation will be used if (1) results in zeros or small ratios. This entails reviewing existing ED records and counting the number of ED providers assigned to patients with OUD, and the number of patients who received ED-initiated BUP with referral for ongoing MAT during the study period.

Note that subsequent sections of this SAP indicate that there are statistical methodologies (a Bayesian approach and a ChangeToOne approach) that can be implemented to handle low frequency outcome. Thus, for the primary analysis the primary implementation outcome will be defined as in (1). A participant will be counted as having received ED-initiated BUP with referral for ongoing MAT if there is evidence that they were administered, prescribed, or provided BUP for take home administration. Further, there must also be evidence that the participant received a referral for opioid use disorder treatment. The primary implementation outcome measure will be scored from the ED Visit Review (EDR) form based on the following two questions:

- Were any of the following medications administered in the ED, prescribed at discharge, and/or given a take home dose?
- Did the patient receive a referral to opioid use disorder (OUD) treatment?

The outcome measure is calculated as an indicator that both of the following are true:

- BUP was administered in the ED, prescribed and/or provided for take home administration (i.e., ERBUPMED='1' or ERBUPRX='1' or ERBUPTH='1').
- Referral made for OUD treatment (i.e., EROPIREF='1')

If either of these criteria are met and the other missing, then the primary outcome measure will be missing. Since the data arise from the electronic health record, missing data should be exceedingly rare.

7.2 Analysis of the Primary Implementation Outcome Measure

For the primary implementation outcome measure, a Generalized Linear Mixed Model will be used to compare the rates of provision of ED-initiated BUP with referral for ongoing MAT between the baseline and IF evaluation periods. The model for the primary analysis will be used to compare the rates of ED-initiated BUP with referral for ongoing MAT between the IFEP and the BEP is:

$$\mathbf{logit}(\boldsymbol{p_{si}}) = \boldsymbol{\alpha} + \boldsymbol{\beta t_{si}} + \boldsymbol{\gamma f_{si}} + \boldsymbol{r_s}$$

where

- p_{si} is the probability of success of patient participant *i* at site *s*,
- t_{si} is the calendar time of enrollment of patient participant *i* at site *s*,
- f_{si} is the indicator of whether patient participant *i* at site *s* is in the BEP (f_{si}=0) or IFEP (f_{si}=1), and
- r_s is the random effect of site *s*, where $r_s \sim N(0, \sigma^2)$.

In this model the γ estimate represents the estimated difference in the logit of the probability of success and captures the effect of implementation facilitation. Note that calendar time is measured in days and defined in Section 3.3.6. The model will test a one-tailed hypothesis at the 0.05 level. The null and alternative hypotheses are:

$$H_0: \gamma \leq 0$$

$$H_a: \gamma > 0.$$

The following SAS code fragment estimates this model:

```
proc glimmix data = primout method = quad;
    class arm (ref="1") site;
    model z = arm time / dist = binomial link = logit solution;
    random intercept / subject = site;
    estimate "trt effect" arm 1 -1 / cl;
    lsmeans arm / cl ilink;
run;
```

where

- primout is the dataset containing the variables required for the primary outcome analyses;
- arm is 1 for the BEP and 2 for the IFEP;
- z is an indicator of the ED-initiated BUP (primary implementation outcome measure); and

- time is defined in Section 3.3.6.

The main focus for estimation is the estimated Risk Difference (RD) defined as the difference between IFEP and BEP success rates, $(p_{IFEP} - p_{EP})$ with its 95% credible interval (CI). The estimated RD and its 95% CI will be obtained using the Bayesian approach outlined in the protocol Appendix 19.0 and in this SAP. Below is the SAS code used for analyzing the primary implementation outcome for this model:

```
proc mcmc data = simmsimul nbi = 1000 nmc = 10000 thin = 2 seed = 159
monitor =(alpha-gammacoeff gammacoeff_gt_0 p1 p2 pdiff) statistics = (summary
intervals);
    parms alpha-gammacoeff 0;
    parms sigma2 1;
    prior alpha-gammacoeff~normal(mean = 0, var = 1000):
    prior sigma2~ igamma(shape = 0.001, scale = 0.001)
    random b0 ~normal(mean = 0, var = sigma2) subject = site;
    array p[2];
    p[treat+1] = logistic(alpha + beta * 22 + gammacoeff * treat);
    pdiff = p2 – p1;
    eta = alpha + beta * time + gammacoeff * treat + b0;
    pi = logistic(eta);
    model z ~binomial(n = trials, p = pi);
    gammacoeff_gt_0 = gammacoeff > 0;
run;
```

where

- simmsimul is the analytic dataset (e.g., simulated dataset in power calculations);
- alpha is the intercept, beta is the coefficient β, gammacoeff is γ and sigma2 is the variance of the random effect of site from the logistic regression model above;
- time is defined in Section 3.3.6;
- treat is equal to ‘0’ for the BEP and ‘1’ for the IFEP;
- p1 is the proportion of success during the BEP;
- p2 is the proportion of successes in the IFEP; and
- pdiff is the risk difference.

The primary implementation outcome will be summarized by site and evaluation period with frequencies and percentages. Results of the Bayesian approach, that is the model specified above, will be presented tabularly with estimates and 95% credible intervals for the risk difference.

7.2.1 ChangeToOne Policy versus the Bayesian Approach

Under the ChangeToOne policy, if a site-arm has "all zeros" data (that is, if all results for that site are zero in either the BEP or IFEP), one randomly chosen value of the zeros is changed into a one. By moving the data back from the edge of the outcome space the analysis method will be spared from having to deal with phenomena such as estimated variances of zero. Note that, under the alternative that the control arm has a lower success probability than the treated arm, this change will tend to move the arms closer together, and thus can be considered conservative.

Note that the protocol stated the ChangeToOne policy (see Section 12.8 of the protocol) would be used for the primary implementation outcome in the presence of any zero counts (i.e., no patient participants received ED-initiated BUP at a particular site in a particular evaluation period). A Bayesian approach (see Section 12.9 of the protocol), which has its own attractions and drawbacks, was also considered as a supportive analysis. Since the risk difference is of primary interest, the Bayesian approach will be used for estimating the risk differences, however the ChangeToOne policy will be used for the primary analysis to calculate a p-value based on the logistic regression model in Section 7.2. This logistic model assumes a linear effect of time on the log-odds scale, which corresponds to a time-varying risk difference scale. Thus the risk differences will be estimated at two time points, and may be calculated for other as well: (1) the last day for which one site was in the BEP and one site was in the IFEP (01/01/2019), and (2) the last day at which both enrolling sites were actively enrolling prior to the pause in enrollment due to the COVID-19 pandemic (03/02/2020).

7.3 Definition of Primary Effectiveness Outcome Measure

The primary effectiveness outcome is patient participant engagement in formal addiction treatment for OUD on the 30th day post-index ED visit. Engagement in formal addiction treatment for OUD is defined as enrollment and receiving formal addiction treatment for OUD on Study Day 30, assessed by patient self-report and confirmed by direct contact with the treating facility and/or treating. Formal addiction treatment for OUD will be those treatments consistent with the American Society of Addiction Medicine's levels of care (1-4) and will include a range of clinical settings including office-based providers of BUP or naltrexone, OTPs, intensive outpatient, inpatient, or residential treatments. Patients do not need to be receiving MAT on the Study Day 30 to be considered engaged in formal addiction treatment for OUD. Participation in a self-help program, such as Narcotics Anonymous, alone will not be considered as engagement in formal addiction treatment for OUD.

The primary effectiveness outcome measure is binary variable reflecting engagement in addiction treatment on the 30th day post-index ED visit. The outcome will be based on patient participant self-report on the Engagement in Treatment: Participant (ETP) form and confirmed by facility and/or treating clinician on the Engagement in Treatment: Facility (ETF) form. If a patient participant reports being in treatment (i.e., ETMEDTRT="1") but there is no confirmation from a facility or clinician (i.e., no ETFs are completed, or all ETENGAGE="0") then the individual is considered not engaged in formal addiction treatment for OUD. Similarly, if there is an ETF indicating the individual was engaged in treatment (i.e., ETENGAGE="1") but this is not in line with self-report (i.e., ETMEDTRT="0", or the facility name does not match the self-reported facility) then the patient participant is considered not engaged in formal addiction treatment for OUD. If there is no ETP completed or if an ETF is completed but the facility did not complete confirmation the individual was engaged in treatment (i.e., ETENGAGE is missing on an existing ETF), then the primary effectiveness outcome is considered missing. If the patient participant self-reports not being in treatment then they are considered not engaged in formal addiction treatment for OUD, regardless of any ETF forms completed. Lastly, if the ETP is not completed but there is an ETF completed, the primary effectiveness outcome measure is considered missing.

7.4 Analysis of Primary Effectiveness Outcome Measure

A mixed effects model will be used to compare the rates of patient engagement in formal addiction treatment for OUD on the 30th day post study enrollment between the baseline and IF evaluation periods. The model for the primary effectiveness will be used to compare the rates of engagement in formal addiction treatment for OUD on the 30th day post-index ED visit between the IFEP and BEP is:

$$\mathrm{logit}(\boldsymbol{p_{si}}) = \boldsymbol{\alpha} + \boldsymbol{\beta t_{si}} + \boldsymbol{\gamma f_{si}} + \boldsymbol{r_s}$$

where

- p_{si} is the probability of success of patient participant *i* at site *s*,
- t_{si} is the calendar time of enrollment of patient participant *i* at site *s*,
- f_{si} is the indicator of whether patient participant *i* at site *s* is in the BEP (f_{si}=0) or IFEP (f_{si}=1), and
- r_s is the random effect of site *s*, where $r_s \sim N(0, \sigma^2)$.

Note that calendar time is measured in days and defined in Section 3.3.6. In this model, γ captures the effect of implementation facilitation. The model will test a one-tailed hypothesis at the 0.05 level. The null and alternative hypotheses are:

$$H_0: \gamma \leq 0$$

$$H_a: \gamma > 0.$$

Similar to the implementation primary outcome, the main focus is the estimated Risk Difference (RD) defined as the difference between IF and Baseline engagement in treatment success rates, ($p_{IFEP} - p_{EP}$) with its 95% credible interval (CI). The estimated RD and its 95% CI will be obtained from the Bayesian approach as described in Section 7.3 for the primary effectiveness outcome. The SAS code will be analogous except p1 is now the proportion of patient participants engaged in formal addiction treatment for OUD on Study Day 30 during the BEP and p2 is the now the proportion during the IFEP.

The primary effectiveness outcome will be summarized by site and evaluation period with frequencies and percentages. Results of the Bayesian approach, that is the model specified above, will be presented tabularly with estimates and 95% credible intervals for the risk difference.

7.5 Supportive Analyses of the Primary Outcome Measures

7.5.1 Subgroup Analyses

The NIH requires subgroup analyses by sex, race, and ethnicity (NIH, 2016). Analyses will be conducted by evaluation period. Primary implementation and effectiveness outcomes will also be summarized by these subgroups and evaluation period.

Several covariates may influence the primary implementation and effectiveness outcome measures. The GLMM models proposed for the primary outcomes will be expanded to adjust for potential effect modifiers. The models will be adjusted for sex, race, and ethnicity as well as each of their interactions with treatment. Below is a mathematical formulation of the models:

$$\mathbf{logit}\,(\boldsymbol{p_{si}}) = \boldsymbol{\alpha} + \boldsymbol{\beta t_{si}} + \boldsymbol{\gamma f_{si}} + \boldsymbol{\theta_1 sex_{si}} + \boldsymbol{\theta_2 race_{si}} + \boldsymbol{\theta_3 ethnicity_{si}} + \boldsymbol{\theta_4 f_{si} * sex_{si}} + \boldsymbol{\theta_5 f_{si} * race_{si}} + \boldsymbol{\theta_6 f_{si} * ethnicity_{si}} + \boldsymbol{r_s}$$

where sex, race, and ethnicity are indicators for their respective variables. They will be entered into the model in a stepwise manner using a p-value cutoff of 0.05. Note that an interaction term will always be included with its corresponding main effects, and the effect of treatment period will be forced into the model. If there are convergence issues, the model will be fit with each demographic factor separately. A forest plot will summarize the estimated risk difference from models where each demographic factor is considered separately to evaluate potential in trends in effect modification, which may not be statistically significant at the 0.05 level.

7.5.2 Sensitivity Analyses

To account for the pause in enrollment due to the COVID-19 pandemic, two separate sensitivity analyses will be conducted on the primary implementation and primary effectiveness outcomes.

First, the date sites closed enrollment due to the pandemic will be considered as the date closed for the study, whereby the analysis will be performed using only pre-COVID-19 data. This will show the “true” effect of the Implementation Facilitation on EDs with no effect of coronavirus. Secondly, the data entered after sites reopened their ED will be converted so that the pause in enrollment is removed. This will show whether the 3-month delay in recruitment due to COVID-19 had an impact on the primary implementation and primary effectiveness outcome analyses.

7.5.3 Missing Data

It is anticipated that there will be no missing data expected for the primary implementation outcome since this entails data abstraction from the EHR after the index ED visit. One scenario in which missing data may arise is if the participant withdraws consent prior to medical record abstraction. To minimize missing data for the primary effectiveness outcome, the initial assessment conducted at the follow-up visit will be the ETP, which can be done over the phone to maximize availability. Per Section 12.10 of the protocol, any missing primary outcome data will be considered as failures (i.e., no provision of ED-initiated BUP, not engaged in formal addiction treatment for OUD on Study Day 30).

7.5.4 Secondary Analyses Related to the Primary Effectiveness Outcome

A secondary analysis akin to the primary effectiveness outcome will also be implemented that addressed potential surveillance bias. Sites continued to attempt contact with patient participants who did not attend the follow-up visit within the targeted window (44 days, inclusive). Due to the stepped wedge this may result in a bias in that patient participants enrolled earlier have more of a chance of obtaining the primary effectiveness outcome. An analysis will be performed for treatment engagement that requires the self-report to have been obtained within the 44-day target window. Other than this requirement the operational definition follows Section 7.3.

An additional secondary analysis of the treatment engagement outcome measure will be implemented to assess whether there are differences in the rate of engagement at Day 30 between those who received ED-initiated BUP and those who did not.

7.6 Definition of Secondary Outcome Measures

7.6.1 Secondary Implementation Outcomes

1. Fidelity to the Critical Action Checklist relating to the provision of ED-initiated BUP with referral for ongoing MAT as captured by the CAC form. For the binary variable, if all critical actions were completed for a given participant the variable will be coded 1 versus 0 if at least one critical action was not completed. The count variable will be defined as the number of critical actions completed per given participant.

2. Rates of enrolled patients with OUD receiving an appointment for opioid treatment provider/program upon ED discharge as recorded on EDR form. The variable will be coded 1 if YES is endorsed on “Did the patient receive a referral to opioid use disorder (OUD) treatment?” and 0 if the answer is NO.

3. Number of ED providers receiving DATA 2000 training as reported on ICH form: The outcome is the number of participants who endorsed YES on the question “In the past year, did you attend or complete a DATA 2000 training on buprenorphine prescribing that would allow you to obtain a DEA waiver?”.

4. Number of clinicians providing ED-initiated BUP with referral for ongoing MAT as reported in ICH form.

5. ED provider readiness and preparedness ruler scores to initiate BUP and provide referral for ongoing MAT as reported on RRL form: The outcome will be scored from the following

questions: “On a scale from 0 to 10, how prepared are you to provide ED-initiated buprenorphine with referral for ongoing medication assisted treatment (MAT) for the treatment of opioid use disorder, where 0 equals “not prepared at all” and 10 equals “totally prepared?” and “On a scale from 0 to 10, how ready are you to provide ED-initiated buprenorphine with referral for ongoing MAT for the treatment of opioid use disorder, where 0 equals “not ready at all” and 10 equals “totally ready?”.

6. ED ORCA scores relating to ED-initiated BUP with referral for ongoing MAT (OE1 and OE2 form). The score will be dichotomized, as was done in Hawk et al.[5], where scores in the first four quintiles were categorized as less ready, and scores in the upper quintile is considered most ready.
7. Community opioid treatment provider/program readiness and preparedness ruler scores to continue MAT for patients with OUD who have received ED-initiated BUP (RRL form).
8. Community opioid treatment provider/program ORCA scores relating to receiving patients with OUD who have received ED-initiated BUP (OC1 and OC2 form).

7.6.2 Secondary Effectiveness Outcomes

1. Self-reported days of illicit opioid use (past seven-days) as measured by TLFB method at 30 days: The following substances collected on TLFB method will be considered illicit opioid use if there is no prescription: heroin, oxycodone, hydrocodone, fentanyl, morphine, hydromorphone, meperidine, methadone, buprenorphine, oxymorphone, pentazocine, and codeine.
2. Overdose events (past 30 days) captured by participant self-report in ODE: The outcome will be scored from question 1 “On how many days in the past 30 days do you think you overdosed on opioids (you used more opioids than you should have used and were more sedated, drugged, or high than you wanted to be)?”
3. HIV risk taking behaviors (past 30 days) as captured in HST.
4. Healthcare service utilization (past 30 days).
5. Rates of illicit opioid negative urines at 30 days as captured on urine drug screen (UDS): A UDS will be considered negative for opioids if all of the following substances are negative: opiates (2000ng), oxycodone, methadone, opiates (300ng), buprenorphine and fentanyl. If a participant tests positive on the UDS for any of these substances and self-reports on TLFB that they have a prescription for any opioid, then the use will be considered licit (i.e., not licit).

7.7 Exploratory Analyses

We will also evaluate a limited set of patient and provider characteristics for their potential effect on successful implementation and effectiveness outcomes. Study participant characteristics to be evaluated are:

- Gender
- Race/Ethnicity
- Health insurance status
- Age
- Primary drug (heroin vs prescription opioids)
- Reason for presentation such as seeking treatment for OUD or overdose

- Referral to office-based BUP provider versus OTP
- Pain Intensity and Interference (PEG scale)
- Opioid overdose at index visit
- Stimulant positive urine toxicology, or self-report at index ED visit
- Fentanyl positive urine toxicology at index ED visit

ED characteristics such as size, location, existing substance abuse services and follow up resources as well as the range and number of addiction treatment services in the catchment area of the ED will be described, as well as ED provider characteristics such as age, gender, years, and level of training will be evaluated.

These analyses will utilize similar models as for the primary analysis, the MIXED models procedure repeated measures and generalized estimating equations (GEE), or other appropriate regression, clustering, and factor analytical tools to evaluate potential impact of site factors and patient characteristics on the primary implementation outcome and effectiveness outcome.

For cost effectiveness analyses, resource costs will include intervention costs incurred in the ED related to the studied intervention, (e.g., cost to provide ED-initiated BUP with referral for ongoing MAT, cost of buprenorphine), downstream medical costs and patient costs of treatment (e.g., time, transportation). Where relevant, we will convert duration of an activity to monetary values by multiplying by provider labor costs. The costs of all addiction and medical treatment (e.g., inpatient, outpatient, treatment center, medication) received by participants will be included in the cost calculations. This information will be collected by self-report through a health service utilization survey. Unit costs of substance abuse and medical treatment will come from the facility surveys or other published estimates.[7] Medication costs will be calculated from the average wholesale price plus the dispensing fee. We will collect Medicare reimbursement rather than Medicaid or commercial insurance amounts for relevant services in the facility surveys because Medicare reimbursement is most likely to reflect marginal costs of service provision. Incremental cost effectiveness ratios will be calculated, defined as $\Delta C/\Delta E$, where ΔC is the difference in costs and ΔE is the difference in effectiveness between the baseline evaluation period and IF evaluation period. Effectiveness is narrowly defined to the primary outcome – engagement in formal addiction treatment for OUD. The drawback of incremental CEA is that because no outcome is comprehensive, analyses do not allow one to directly compare interventions with different outcome measures. Yet, policymakers may still value this information when choosing among competing programs. Researchers often use the outcome Quality Adjusted Life Years (QALYs) to enable comparisons across interventions. In the case of OUD, many of the benefits accrue to individuals other than the individual being treated and would not be captured in this metric making this outcome less appealing. Because health care costs are typically highly skewed, we will consider several cost estimation models. We will not include monetized values of societal outcomes (i.e., reduced criminal activity) because inclusion of these monetized values of these outcomes in the numerator of the cost effectiveness ratio would lead to double counting of these outcomes (e.g., their monetized value would be counted in the numerator and then counted again as the value of being abstinent in the denominator of the cost effectiveness ratios). We will not include training or research costs because these costs would not be incurred in standard care. Our primary outcome will be cost effectiveness acceptability curves, which indicate the probability different implementation strategies are cost effectiveness at different willingness to pay threshold values of the studied outcome. For cost estimates which are subject to debate either because of known imprecision in the estimation procedures or lack of adequate information, we will conduct sensitivity analyses with the goal of explaining the ways in which different assumptions would impact study results.

We will use appropriate non-parametric, parametric, and analysis of variance statistical procedures to descriptively evaluate the key characteristics of each study site (e.g., patient flow indicators such as length of stay of treated and released patients, and demographic and drug use characteristics of patients with OUD presenting at each ED site, indicators of organizational level differences between the sites (e.g., the number ED providers, number/ratio ED providers DEA waivered to prescribe BUP), and to evaluate comparability of baseline characteristics among patient cohorts enrolled at each of the study sites and overall during baseline evaluation period and the IF evaluation periods across all sites.

The EHR-abstracted data regarding MOUD activities in the ED will be summarized by site at the following time points separately: (1) the entire BEP; and (2) the entire IFEP. Exploratory analyses may evaluate whether any of the measures in this dataset are associated with primary implementation factors.

8.0 SAFETY ANALYSIS

Safety information for patient participants includes self-reported emergency department visits, hospitalizations, overdoses, and suicidal ideation, as well as deaths (not identified from a National Death Index search). Regardless of when the Day 30 follow-up visit occurs, deaths are only collected if the date of death occurred between the index ED visit and Study Day 30 (i.e., the safety window). This information will be summarized separately for those participants who were enrolled and later found to be ineligible. Note that deaths in the safety window are the only reportable adverse events.

8.1 ED Visits and Hospitalizations

The total number of ED visits and hospitalizations reported on the EDV form at the Day 30 follow-up visit, as well as the number reported per participant, will be summarized by evaluation period. Listings of ED visits and hospitalizations, including relationship to substance abuse disorder, will be presented by evaluation period and will include hospitalization or ED Visit date, discharge date, chief complaint, and discharge diagnosis. A listing will also be created for participants enrolled who were later found to have been ineligible, if any follow-up EDV data is available for them.

8.2 Overdose Events

Overdose events reported on the ODE form at the Day 30 follow-up visit will be summarized by evaluation period. A listing of overdose events report at the follow-up visit will be presented by site for each evaluation period and will include number of days the participant overdosed on opioids and the number of days the participant needed medical assistance for an opioid overdose in the past 30 days. A listing will also be created for participants enrolled who were later found to have been ineligible if any ODE data is available for them at the follow-up visit.

8.3 Suicide Risk

A listing of patient participants endorsing suicidal ideation on the HST at baseline or the Day 30 Follow-up visits will be presented for each evaluation period by site. A listing will also be created for participants enrolled who were later found to have been ineligible. Patient participants are considered to have endorsed suicidality on the HST if they indicate several days, more than half the days, or nearly every day having thoughts they are better off dead or of hurting themselves.

8.4 Deaths

Deaths occurring in the safety window will be coded using the MedDRA® dictionary version 23.1. A listing of deaths will be presented by evaluation period and will include description of death, date of enrollment, date of death, relatedness to overdose, MedDRA® coded Preferred Term and

System Organ Class. A listing will also be created for participants enrolled who were later found to have been ineligible. Narratives of deaths will also be provided.

9.0 SIGNIFICANCE TESTING AND MULTIPLICITY

As this study is designed as a Hybrid Type 3 Effectiveness-Implementation study, the implementation is the main outcome, and so when measuring success of the study, the implementation outcome will take precedence over the effectiveness outcome, which will be considered secondary. Therefore, considering the effectiveness aim as secondary, it is not necessary to put in procedures to control the type 1 error across multiple outcomes. Multiple-comparison adjustments are not anticipated when performing secondary analyses. To be mindful of the multiple testing problem, secondary findings will be reported as noteworthy hypothesis-generating results only when their p-values are considerably smaller than 0.05.

10.0 SAMPLE SIZE AND POWER

This section presents power simulation for the implementation of the primary outcome. The power simulations to assess the adequacy of the sample size followed the method of Parzen[6]. The Parzen method simulates many vectors of (0,1) random variates with specified probabilities and a specified positive intraclass correlation (ICC) ρ, for a given sample size. Assuming 240 patients will be enrolled at each of the 4 sites, power simulations were done for four parameter sets (scenarios) under both the alternative and null hypotheses as a function of an assumed ICC. The first 120 elements of each vector all had a common probability p_1, while the second 120 elements had a common probability p_2, where (p_1, p_2) are assumed probabilities of success in the baseline and IF evaluation periods, respectively. Each vector thus specifies in its first half a site's outcomes in the baseline evaluation period, while in the second half it specifies outcomes in the IF evaluation period. Four such vectors (one for each site) comprised the data for a single iteration of the simulation. Each element in each vector was assigned a month as per the protocol study timeline. However, month by itself played no role in determining the probability of success of any vector elements. For each setting of the parameters (p_1, p_2, ρ), 10,000 replicates were generated (that is, 40,000 vectors) for simulation analysis.

A Stepped-Wedge model was used to analyze simulated data. Simulated power was taken to be the proportion of the 10,000 GLIMMIX runs that had a significant one-tailed type 3 p-value for arm at alpha level 0.05. The "arm" refers to baseline (control) and IF evaluation (intervention) periods. Power analysis were not adjusted/ controlled for type I error. Because in this study it is likely the implementation outcome will have a low probability in the baseline evaluation period coupled with a large ICC, other analytical methods were identified that had better power characteristics than the GLIMMIX approach outlined above. One method is to substitute historical data for the relevant site(s) for the data in the "all zeros" arm. Problems with this approach are (1) there is no guarantee that the historical data will not be "all zeros", and (2) it is difficult to simulate the effect of this policy beforehand. The following section presents power analysis for another alternative approach, ChangeToOne policy, and a final approach, Bayesian analysis, is outlined in Appendix 15.0.

Details of the power simulations and results are given in Section 12 of the protocol.

10.1 ChangeToOne Policy

This rather naïve approach actually results in a surprisingly beneficial effect on the "Implementation Outcome with low p_1", without appreciably changing power for the other scenarios (in which the probability of an "all zero" site-arm is very low, so the ChangeToOne policy seldom has an effect).

10.1.1 Bayesian Approach

A Bayesian approach, which has its own attractions and drawbacks, was also considered. We detail these in the Appendix of the SAP (Section 17.0). Based on the power calculations previously presented, having sites with "all zeros" in the BEP degrades power no corrective action is made. While the "ChangeToOne" policy seems a simple and attractive remedy, the Bayesian approach with diffuse priors leads to similar conclusions. Except for the credibility intervals for $p_2 - p_1$, increasing ICC degrades precision. Due to these findings and the interpretational focus on the risk difference, the main analysis of the primary implementation will utilize the Bayesian approach, with diffuse priors, instead of the ChangeToOne Approach, as noted previously in Section 7.2.1.

10.2 Summary of Power Simulations

Under the most optimistic scenarios investigated (Implementation Outcome with low p1 and Efficacy Outcome with high p2) and ICC < 0.3 (and perhaps higher), power for the envisioned design exceeds 0.8 under the ChangeToOne policy and Bayesian approach. Other scenarios featuring less separation between groups have lower power. Having sites with "all zeros" in the baseline arm degrades power if you take no corrective action. The "ChangeToOne" policy seems a simple and attractive remedy. A Bayesian approach with diffuse priors leads to conclusions similar to those from the ChangeToOne policy (see Appendix 17.0). Except for the credibility intervals for $p_2 - p_1$, increasing ICC degrades precision.

11.0 INTERIM ANALYSES AND DATA MONITORING

No interim looks at primary or secondary outcomes were planned for this study. However, a power and sample size recalculation was to be performed based on the primary outcome rates and enrollment rates observed in the baseline evaluation period. The power and sample size recalculation was to be done no earlier than the end of the first site's baseline evaluation period.

Due to extremely low recruitment at one site, and fairly low recruitment at another site during the BEP, alternate power and sample size recalculations were implemented to evaluate the impact of this and several possible design changes. The first evaluation, in December 2017, simulations were conducted for the following scenarios:

- assumed future enrollment rates would proceed as expected for all sites;
- observed enrollment rates would continue for the remainder of recruitment at all sites;
- add three months of recruitment to each evaluation period and assume expected recruitment rate at all sites going forward;
- add three months of recruitment to each evaluation period and assume observed recruitment rates at all sites going forward;
- extend the IFEP by six months assuming expected enrollment rate for all future enrollments; and
- extend the IFEP by six months assuming all future recruitments occur at the observed rate.

These power curves did not indicate any substantial improvement over the original calculations in the protocol, and no changes were made to the design.

The second evaluation occurred in June 2018 when it was observed that the other two sites would exceed the target sample size of 120 in the BEP. The question of interest was whether recruitment should be slowed down at these high enrolling sites. Three different patterns of recruitment were

considered for the IFEP, with none showing substantial changes in power. No changes were made to the study implementation or expected recruitment rates for each site.

12.0 DATA QUALITY

12.1 Data Audits

A summary of data audit results from site interim monitoring visits conducted by CCC monitors will be presented by site.

12.2 Protocol Deviations

Protocol deviations will be summarized by site and by evaluation period for both the patient participants and provider participants. The summaries will include the number of deviations reported, the number of individuals impacted (if any), frequencies for the types of protocol deviations, and information on whether the protocol deviation was deemed minor or major. Detailed listings of protocol deviations by evaluation period and deviation category will also be provided.

13.0 SOFTWARE TO BE USED FOR ANALYSES

All statistical analyses performed by the DSC will use SAS® Version 9.4 software.

14.0 UPDATES TO THE STATISTICAL ANALYSIS PLAN

Protocol Version	Updated SAP Version Number	Section number changed	Description and reason for change	Date updated SAP was approved
4.0	1.0	N/A	Initial version	14-JUN-2021
4.0	2.0	Changes throughout		20-SEP-2021

15.0 LIST OF PROPOSED TABLES, FIGURES, AND LISTINGS

The below listing contains the tables, figures, and listings which will be provided by the DSC.

Category	Table Number	Title	Responsible Party
Enrollment, Patient Participant Disposition and Follow-up	Figure 1A	Patient Participants Flow Diagram – Baseline Evaluation Period	DSC
	Figure 1B	Patient Participants Flow Diagram – IF Evaluation Period	DSC
	1	Summary of Screen Failures by Evaluation Period	DSC
	2	Summary of Screen Failures by Site	DSC
	3	Summary of Enrollment by Evaluation Period	DSC
	4	Summary of Enrollment by Site	DSC
	Figure 2	Proposed versus Actual Enrollments for Baseline Evaluation Period by Site	DSC
	Figure 3	Proposed versus Actual Enrollments for IF Evaluation Period by Site	DSC
	5	Summary of Patient Participant Disposition by Evaluation Period	DSC
	6	Summary of Patient Participant Disposition by Site	DSC
	7	Summary of Attendance at Follow-up Visit by Evaluation Period	DSC
	8	Summary of Attendance at Follow-up Visit by Site	DSC
	9	Summary of Enrolled Patient Participants Who Were Ineligible by Evaluation Period	DSC
	10	Summary of Enrolled Patient Participants Who Were Ineligible by Site	DSC
Baseline Patient Participant Characteristics	11	Summary of Patient Participant Baseline Characteristics by Evaluation Period	DSC
	12	Summary of Patient Participant Baseline Characteristics by Site	DSC

Category	Table Number	Title	Responsible Party
Primary Implementation Outcome	13	Summary of Primary Implementation Outcome by Evaluation Period	DSC
	14	Summary of Primary Implementation Outcome by Site and Evaluation Period	DSC
	15	Analysis Results for Primary Implementation Outcome	DSC
	16	Summary of Primary Implementation Outcome by Sex and Evaluation Period	DSC
	17	Summary of Primary Implementation Outcome by Race and Evaluation Period	DSC
	18	Summary of Primary Implementation Outcome by Ethnicity and Evaluation Period	DSC
	19	Final Covariate Adjusted Model Analysis Results for Primary Implementation Outcome	DSC
	20	Summary of MOUD Practices by Site Over Time from EHR-abstracted Data	DSC
	21	COVID-19 Sensitivity Analyses Results for Primary Implementation Outcome	DSC
Primary Effectiveness Outcome	22	Summary of Primary Effectiveness Outcome by Evaluation Period	DSC
	23	Summary of Primary Effectiveness Outcome by Site and Evaluation Period	DSC
	24	Analysis Results for Primary Effectiveness Outcome	DSC
	25	Summary of Primary Effectiveness Outcome by Sex and Evaluation Period	DSC
	26	Summary of Primary Effectiveness Outcome by Race and Evaluation Period	DSC
	27	Summary of Primary Effectiveness Outcome by Ethnicity and Evaluation Period	DSC

Category	Table Number	Title	Responsible Party
	28	Final Covariate Adjusted Model Analysis Results for Primary Effectiveness Outcome	DSC
	29	COVID-19 Sensitivity Analyses Results for Primary Effectiveness Outcome	DSC
	30	Summary of Treatment Engagement at Day 30 during the IF Evaluation Period by ED-initiated BUP Status: All Patient-participants (Missing Engagement Status Imputed as Not Engaged)	DSC
	31	Summary of Treatment Engagement at Day 30 during the IF Evaluation Period by ED-initiated BUP Status: Patient-participants with Non-missing Engagement Status (No Imputation)	DSC
	32	Analysis of Treatment Engagement at Day 30 during the IF Evaluation Period by ED-initiated BUP Status	DSC
Safety	33	Summary of Hospitalizations Post Index Visit by Evaluation Period	DSC
	Listing 1	Listing of Hospitalizations Post Index Visit by Evaluation Period	DSC
	Listing 2	Listing of Hospitalizations Post Index Visit for Ineligible Participants	DSC
	34	Summary of ED Visits Post Index Visit by Evaluation Period	DSC
	Listing 3	Listing of ED Visits Post Index Visit by Evaluation Period	DSC
	Listing 4	Listing of ED Visits Post Index Visit for Ineligible Participants	DSC
	35	Summary of Overdoses by Evaluation Period	DSC
	Listing 5	Listing of Overdoses by Evaluation Period	DSC
	Listing 6	Listing of Overdoses for Ineligible Participants	DSC
	36	Summary of Suicide Risk by Evaluation Period	DSC

Category	Table Number	Title	Responsible Party
	Listing 7	Listing of Suicide Risk by Evaluation Period	DSC
	Listing 8	Listing of Suicide Risk for Ineligible Participants	DSC
	Listing 9	Listing of Deaths by Evaluation Period	DSC
Data Quality	37	Summary of Data Audits by Site	DSC
	38	Summary of Protocol Deviations by Site	DSC
	Listing 10	Listing of Protocol Deviations by Site	DSC
Provider Participant Characteristics	39	Summary of Provider Participant Characteristics by Site	DSC
Focus Group Participant Characteristics	40	Summary of Focus Group Participant Characteristics: Wave 1	DSC
	41	Summary of Focus Group Participant Characteristics: Wave 2	DSC
	42	Summary of Focus Group Participant Characteristics: Wave 3	DSC

16.0 SHELLS FOR PROPOSED TABLES, FIGURES AND LISTINGS

16.1 Patient Participant Data

16.1.1 Enrollment, Disposition and Follow-up

Figure 1A: Patient Participants Flow Diagram – Baseline Evaluation Period

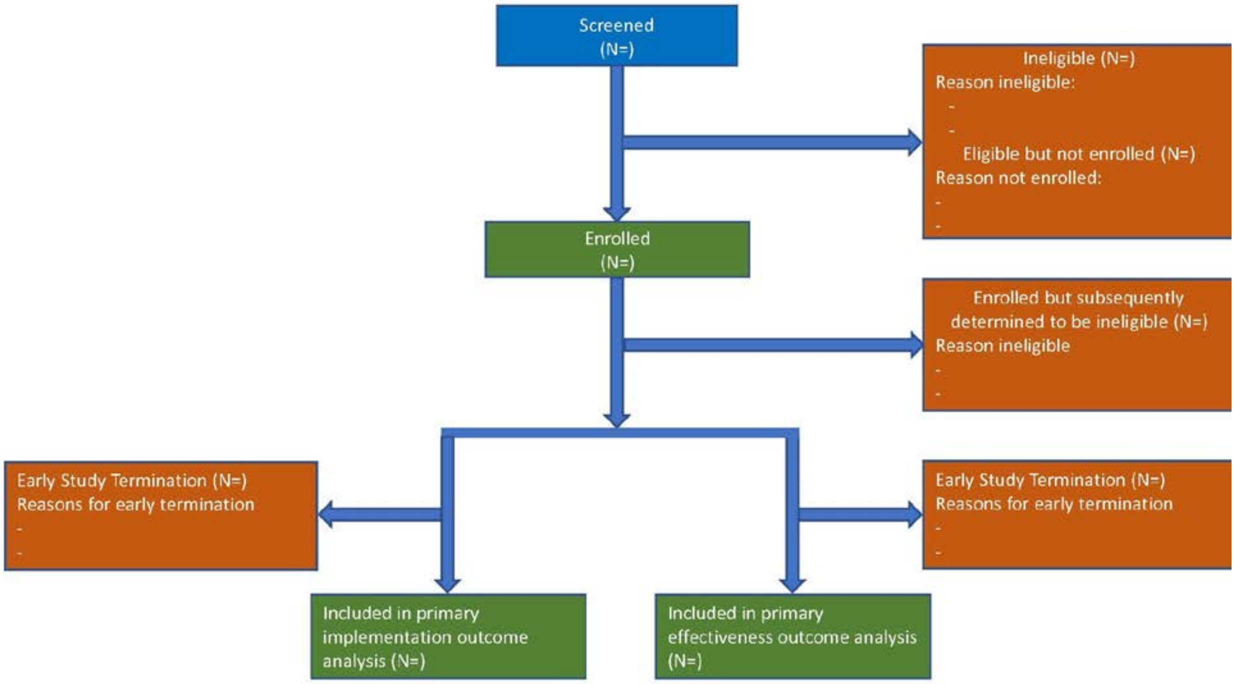

Figure 1B: Patient Participants Flow Diagram – IF Evaluation Period

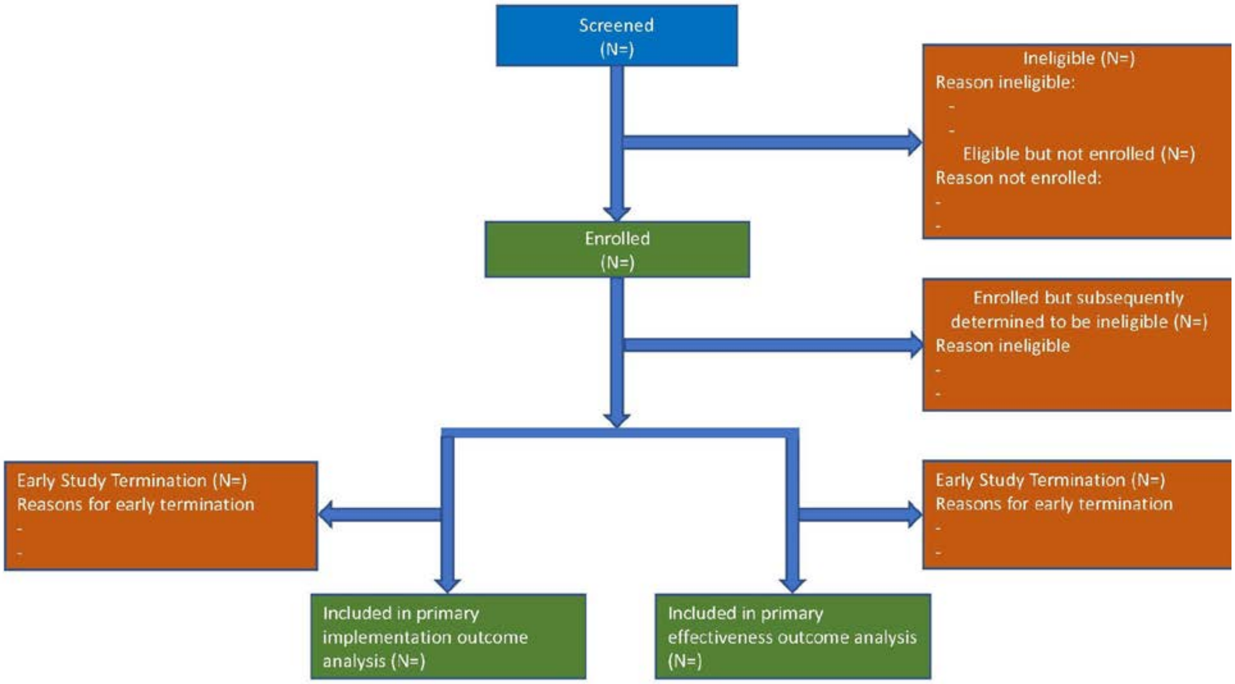

Table 1: Summary of Screen Failures by Evaluation Period			
	**Baseline Evaluation Period**	**IF Evaluation Period**	**Total**
Number verbally consented	N		
Number of screen failures	N (XX.X%)		
Failed the following eligibility criteria[1]			
Did not indicate opioid use in the past 7 days	N (XX.x%)		
Prescribed opioids for a pain condition	N (XX.x%)		
Currently enrolled in formal addiction treatment or inpatient overnight facility	N (XX.x%)		
Did not meet DSM-5 diagnostic criteria for moderate to severe OUD	N (XX.x%)		
Refused	N (XX.x%)		
Medical or psychiatric condition that requires hospitalization at the index ED visit	N (XX.x%)		
Unable to provide reliable locator information including two contact numbers	N (XX.x%)		
Urine toxicology test not positive for opioids	N (XX.x%)		
Actively suicidal or severely cognitively impaired	N (XX.x%)		
Currently a prisoner, awaiting trial, on probation, under house arrest or in police custody	N (XX.x%)		
Previously enrolled in this study	N (XX.x%)		
Unable to speak English	N (XX.x%)		
Presents from extended care facility	N (XX.x%)		
Not 18 years or older	N (XX.x%)		
Did not present to the ED during study screening hours	N (XX.x%)		
Missed	N (XX.x%)		
Unwilling to follow study procedures	N (XX.x%)		
Other	N (XX.x%)		
Unknown	N (XX.x%)		

CONFIDENTIAL

Table 1: Summary of Screen Failures by Evaluation Period			
	**Baseline Evaluation Period**	**IF Evaluation Period**	**Total**
Number of patient participants eligible but not enrolled	N (XX.x%)		
Reasons for not being enrolled[2]			
Missing	N (XX.x%)		
Failed to return to clinic	N (XX.x%)		
Declined study participation	N (XX.x%)		
Death	N (XX.x%)		
Other	N (XX.x%)		

[1] Percentages are calculated based on the denominator of the number of ineligibles and may not sum to 100% if multiple eligibility criteria are not met for potential patient participants.

[2] Percentages are calculated based on the denominator of the number of patient participants eligible but not enrolled.

Table 2: Summary of Screen Failures by Site

	MA Johns Hopkins ED	GNY Mount Sinai ED/Beth Israel	OV University of Cincinnati ED	PNW Harborview Med Center ED	Total
Number verbally consented	N				
Number of screen failures	N (XX.x%)				
Failed the following eligibility criteria[1]					
Did not indicate opioid use in the past 7 days	N (XX.x%)				
Prescribed opioids for a pain condition	N (XX.x%)				
Currently enrolled in formal addiction treatment or inpatient overnight facility	N (XX.x%)				
Did not meet DSM-5 diagnostic criteria for moderate to severe OUD	N (XX.x%)				
Refused	N (XX.x%)				
Medical or psychiatric condition that requires hospitalization at the index ED	N (XX.x%)				
Unable to provide reliable locator information including two contact numbers	N (XX.x%)				
Urine toxicology test not positive for opioids	N (XX.x%)				
Actively suicidal or severely cognitively impaired	N (XX.x%)				
Currently a prisoner, awaiting trial, on probation, under house arrest or in police custody	N (XX.x%)				
Previously enrolled in this study	N (XX.x%)				
Unable to speak English	N (XX.x%)				
Presents from extended care facility	N (XX.x%)				
Not 18 years or older	N (XX.x%)				
Did not present to the ED during study screening hours	N (XX.x%)				
Missed	N (XX.x%)				
Unwilling to follow study procedures	N (XX.x%)				
Other	N (XX.x%)				
Unknown	N (XX.x%)				

Table 2: Summary of Screen Failures by Site

	MA Johns Hopkins ED	GNY Mount Sinai ED/Beth Israel	OV University of Cincinnati ED	PNW Harborview Med Center ED	Total
Number of patient participants eligible but not enrolled	N				
Reasons for not being enrolled[2]					
Missing	N (XX.x%)				
Failed to return to clinic	N (XX.x%)				
Declined study participation	N (XX.x%)				
Death	N (XX.x%)				
Other	N (XX.x%)				

[1] Percentages are calculated based on the denominator of the number of ineligibles and may not sum to 100% if multiple eligibility criteria are not met for potential patient participants.
[2] Percentages are calculated based on the denominator of the number of patient participants eligible but not enrolled.

Table 3: Summary of Enrollment by Evaluation Period

Evaluation Period	Proposed Enrollment	Actual Enrollments, Cumulative	Actual/Proposed
Baseline Evaluation Period	N	N	XX.x%
IF Evaluation Period			
Total			

Table 4: Summary of Enrollment by Site

Site	Proposed Enrollment	Actual Enrollments, Cumulative	Actual/Proposed
MA Johns Hopkins ED	N	N	XX.x%
GNY Mount Sinai ED			
OV University of Cincinnati ED			
PNW Harborview Medical Center ED			
Total			

Figure 2: Proposed versus Actual Enrollments for Baseline Evaluation Period by Site

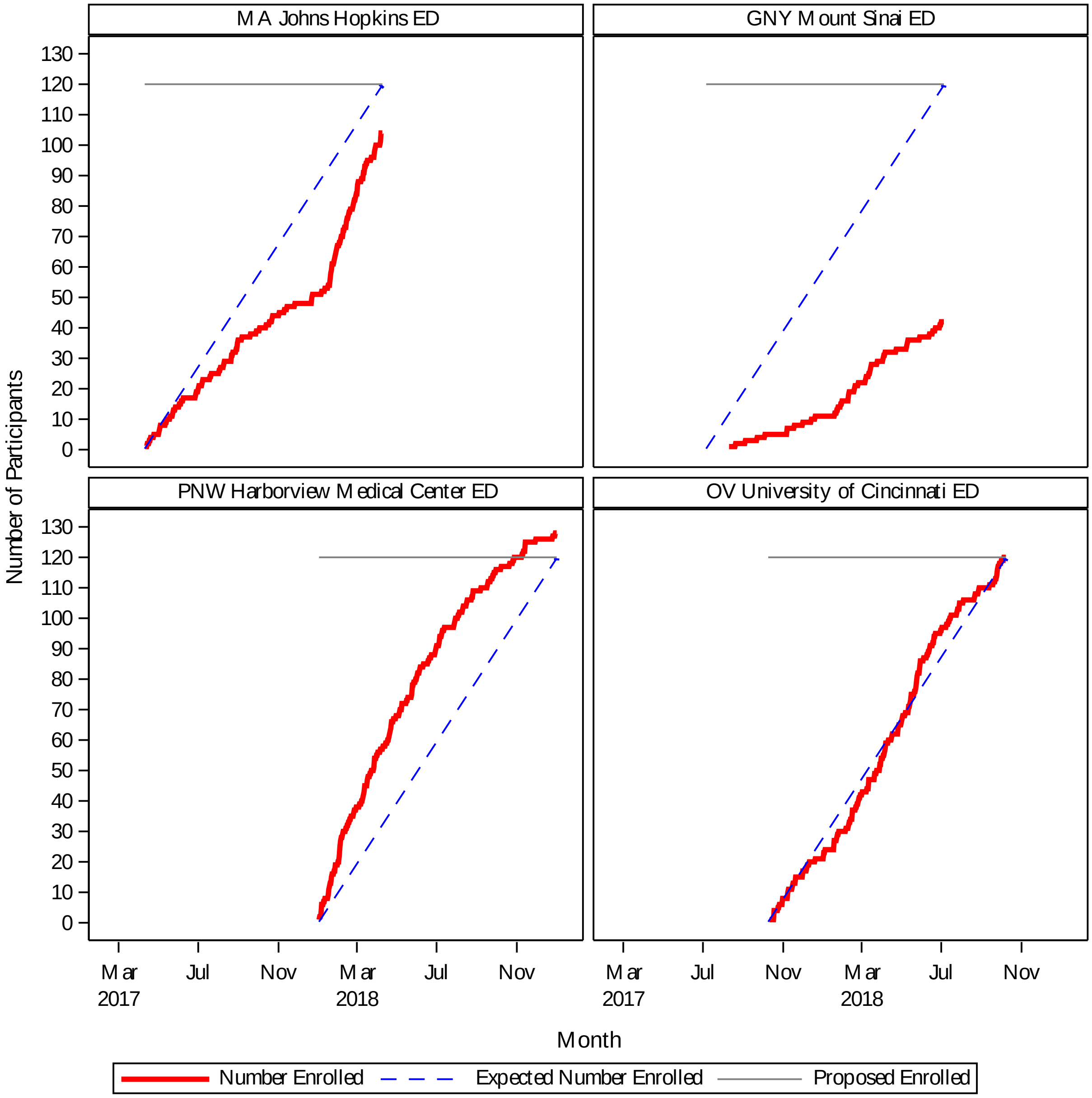

Figure 3: Proposed versus Actual Enrollments for IF Evaluation Period by Site

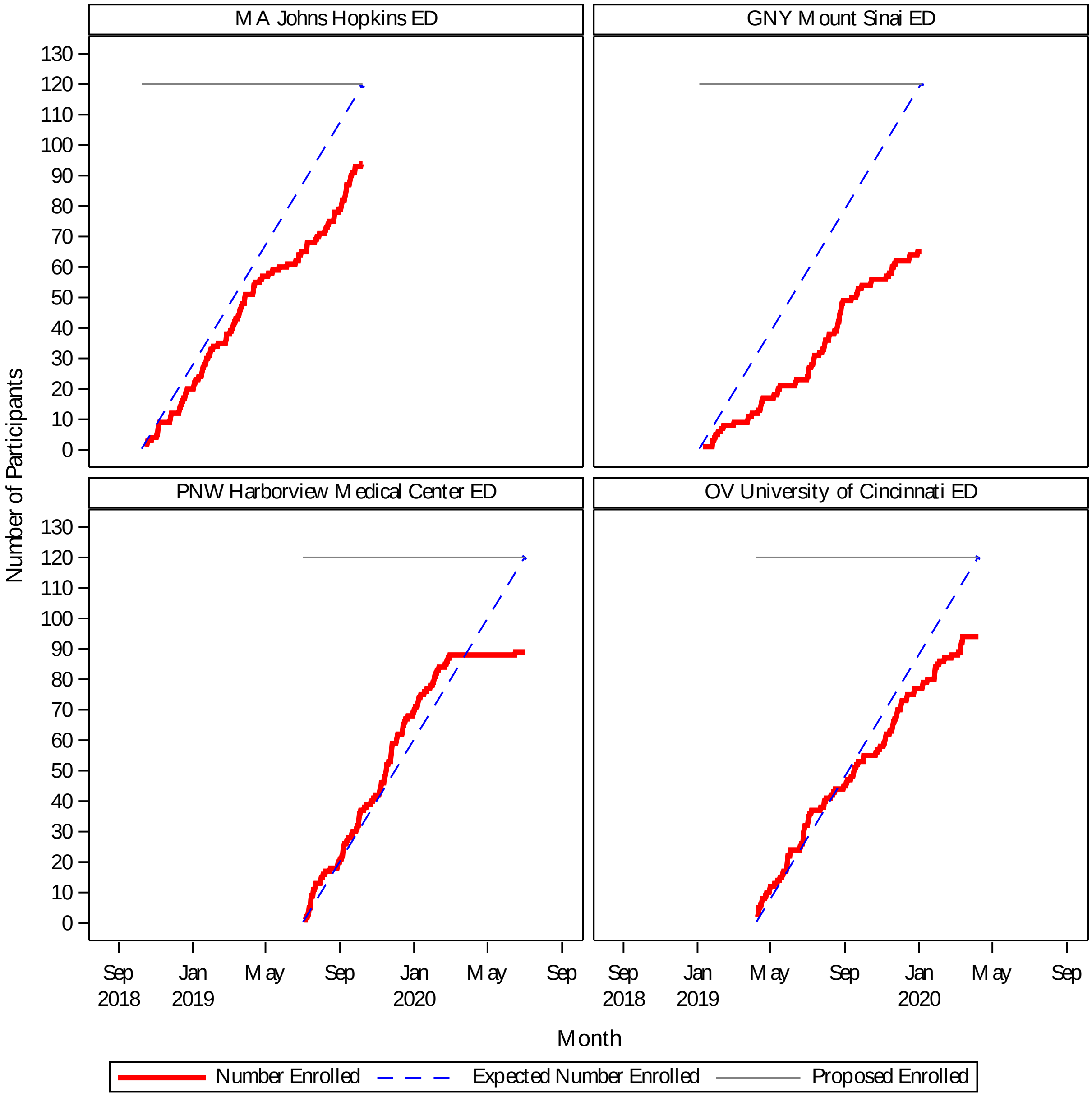

Table 5: Summary of Patient Participant Disposition by Evaluation Period			
	**Baseline Evaluation Period**	**IF Evaluation Period**	**Total**
Number of patient participants enrolled	N		
Number of study completers[1]	N (XX.x%)		
Number who completed the Day 30 follow-up visit within the window[2]	N (XX.x%)		
Number of non-completers	N (XX.x%)		
Reasons for non-completion			
Failed to return to clinic and unable to contact	N (XX.x%)		
Incarcerated	N (XX.x%)		
Deceased	N (XX.x%)		
Terminated due to practical problems (no childcare, transportation, other)	N (XX.x%)		
Moved from area	N (XX.x%)		
Terminated due to AE/SAE	N (XX.x%)		
Terminated for other reason	N (XX.x %)		
Significant psychiatric risk (suicidal, homicidal, psychotic)	N (XX.x %)		
Withdrew consent/assent	N (XX.x %)		
Terminated for administrative issues	N (XX.x %)		
Terminated due to pressure or advice from outsiders	N (XX.x %)		
Feels treatment no longer necessary, cured	N (XX.x %)		
Feels treatment no longer necessary, not working	N (XX.x %)		

[1] Patient participants are defined as study completers if the Day 30 follow-up visit is completed as noted on the STC.

[2] Between 30 and 44 days, inclusive, past enrollment.

Table 6: Summary of Patient Participant Disposition by Site

	MA Johns Hopkins ED	GNY Mount Sinai ED	OV University of Cincinnati ED	PNW Harborview Medical Center ED	Total
Number of patient participants enrolled	N				
Number of study completers[1]	N (XX.x%)				
Number who completed the Day 30 follow-up visit within the window[2]	N (XX.x%)				
Number of non-completers	N (XX.x%)				
Reasons for non-completion					
Failed to return to clinic and unable to contact	N (XX.x%)				
Incarcerated	N (XX.x%)				
Deceased	N (XX.x%)				
Terminated due to practical problems (no childcare, transportation, other)	N (XX.x%)				
Moved from area	N (XX.x%)				
Terminated due to AE/SAE	N (XX.x%)				
Terminated for other reason	N (XX.x %)				
Significant psychiatric risk (suicidal, homicidal, psychotic)	N (XX.x %)				
Withdrew consent/assent	N (XX.x %)				
Terminated for administrative issues	N (XX.x %)				
Terminated due to pressure or advice from outsiders	N (XX.x %)				
Feels treatment no longer necessary, cured	N (XX.x %)				
Feels treatment no longer necessary, not working	N (XX.x %)				

[1] Patient participants are defined as study completers if the Day 30 follow-up visit is completed as noted on the STC.

[2] Between 30 and 44 days, inclusive, past enrollment.

Table 7: Summary of Attendance at Follow-up Visit by Evaluation Period

Evaluation Period	Number Enrolled	Number of Expected Follow-up Visits[1]	Number of Follow-up Visits Attended[2]	Percent of Follow-up Visits Attended
Baseline Evaluation Period	N	N	N	XX.x%
IF Evaluation Period	N	N	N	XX.x%
Total	N	N	N	XX.x%

[1] Follow-up visits are expected 14 days after the target date, which is 30 days post enrollment.

[2] A follow-up visit is considered attended upon the availability of the Engagement in Treatment: Patient (ETP) form.

Table 8: Summary of Attendance at Follow-up Visit by Site

Site	Number Enrolled	Number of Expected Follow-up Visits[1]	Number of Follow-up Visits Attended[2]	Percent of Follow-up Visits Attended
MA Johns Hopkins ED	N	N	N	XX.x %
GNY Mount Sinai ED	N	N	N	XX.x %
OV University of Cincinnati ED	N	N	N	XX.x %
PNW Harborview Medical Center ED	N	N	N	XX.x %
Total	N	N	N	XX.x %

[1] Follow-up visits are expected 14 days after the target date, which is 30 days post enrollment.

[2] A follow-up visit is considered attended upon the availability of the Engagement in Treatment: Patient (ETP) form.

Table 9: Summary of Enrolled Patient Participants Who Were Ineligible by Evaluation Period

	Baseline Evaluation Period	IF Evaluation Period	Total
Number of enrolled patient participants who were subsequently determined to be ineligible	N	N	N
Reasons for ineligibility			
Missing/Unknown	N (XX.X%)		
Will be admitted	N (XX.X%)		
Already in treatment	N (XX.X%)		
Previously enrolled in this study	N (XX.X%)		
Refused	N (XX.X%)		
Actively suicidal or cognitively impaired	N (XX.X%)		
Currently prescribed opioids	N (XX.X%)		
In police custody	N (XX.X%)		
Non-English speaking	N (XX.X%)		
Presenting from extended care facility	N (XX.X%)		
Under 18 years of age	N (XX.X%)		
Missed/patient participant left	N (XX.X%)		

CONFIDENTIAL

Table 10: Summary of Enrolled Patient Participants Who Were Ineligible by Site

	MA Johns Hopkins ED	GNY Mount Sinai ED	OV University of Cincinnati ED	PNW Harborview Medical Center ED	Total
Number of enrolled patient participants who were subsequently determined to be ineligible	N	N	N	N	N
Reasons for ineligibility					
Missing/Unknown	N (XX.X%)				
Will be admitted	N (XX.X%)				
Already in treatment	N (XX.X%)				
Previously enrolled in this study	N (XX.X%)				
Refused	N (XX.X%)				
Actively suicidal or cognitively impaired	N (XX.X%)				
Currently prescribed opioids	N (XX.X%)				
In police custody	N (XX.X%)				
Non-English speaking	N (XX.X%)				
Presenting from extended care facility	N (XX.X%)				
Under 18 years of age	N (XX.X%)				
Missed/patient participant left	N (XX.X%)				

16.1.2 Baseline Characteristics

Table 11: Summary of Patient Participant Baseline Characteristics by Evaluation Period

	**Baseline Evaluation Period (N=)**	**IF Evaluation Period (N=)**	**Total (N=)**
**Demographics**			
Sex			
Male	N (XX.x %)		
Female	N (XX.x %)		
Age (Mean (SD))	N (X.X)		
Age			
< 18	N (XX.x %)		
18 - < 25	N (XX.x %)		
25 - < 35	N (XX.x %)		
35 - < 45	N (XX.x %)		
45 - < 55	N (XX.x %)		
55 - < 65	N (XX.x %)		
65 - < 75	N (XX.x %)		
75+	N (XX.x %)		
Ethnicity			
Not Hispanic or Latino	N (XX.x %)		
Hispanic or Latino	N (XX.x %)		
Don't know	N (XX.x %)		
Refused to answer	N (XX.x %)		
Race			
American Indian or Alaska Native	N (XX.x %)		
Asian	N (XX.x %)		
Black or African American	N (XX.x %)		
Native Hawaiian or Pacific Islander	N (XX.x %)		
White	N (XX.x %)		
Other	N (XX.x %)		
Multiracial	N (XX.x %)		
Don't know	N (XX.x %)		
Refused to answer	N (XX.x %)		

Table 11: Summary of Patient Participant Baseline Characteristics by Evaluation Period

	Baseline Evaluation Period (N=)	IF Evaluation Period (N=)	Total (N=)
Education completed			
Less than high school diploma	N (XX.x %)		
High school graduate	N (XX.x %)		
GED or equivalent	N (XX.x %)		
Some college, no degree	N (XX.x %)		
Associate's degree: occupational, technical, or vocational program	N (XX.x %)		
Associate's degree: academic program	N (XX.x %)		
Bachelor's degree	N (XX.x %)		
Master's degree	N (XX.x %)		
Professional school degree	N (XX.x %)		
Doctoral degree	N (XX.x %)		
Marital status			
Married	N (XX.x %)		
Widowed	N (XX.x %)		
Divorced	N (XX.x %)		
Separated	N (XX.x %)		
Never married	N (XX.x %)		
Living with partner	N (XX.x %)		
Don't know	N (XX.x %)		
Employment			
Working now	N (XX.x %)		
Only temporarily laid off, sick leave, or maternity leave	N (XX.x %)		
Looking for work, unemployed	N (XX.x %)		
Retired	N (XX.x %)		
Disabled permanently or temporarily	N (XX.x %)		
Keeping house	N (XX.x %)		
Student	N (XX.x %)		
Other	N (XX.x %)		

Table 11: Summary of Patient Participant Baseline Characteristics by Evaluation Period			
	Baseline Evaluation Period (N=)	IF Evaluation Period (N=)	Total (N=)
Severity of opioid use disorder			
None			
Mild			
Moderate			
Severe			
Missing			
**Self-reported Substance Use**			
Any substance			
N			
Mean			
SD			
Median			
Min			
Max			
Opioids			
N			
Mean			
SD			
Median			
Min			
Max			
Opioid analgesics[1] (with prescription)			
N			
Mean			
SD			
Median			
Min			
Max			
Opioid analgesics[1] (illicit)			
N			
Mean			
SD			

Table 11: Summary of Patient Participant Baseline Characteristics by Evaluation Period			
	**Baseline Evaluation Period (N=)**	**IF Evaluation Period (N=)**	**Total (N=)**
Median			
Min			
Max			
Heroin			
N			
Mean			
SD			
Median			
Min			
Max			
Fentanyl			
N			
Mean			
SD			
Median			
Min			
Max			
Buprenorphine			
N			
Mean			
SD			
Median			
Min			
Max			
Methadone			
N			
Mean			
SD			
Median			
Min			
Max			

Table 11: Summary of Patient Participant Baseline Characteristics by Evaluation Period

	Baseline Evaluation Period (N=)	IF Evaluation Period (N=)	Total (N=)
Methamphetamine			
N			
Mean			
SD			
Median			
Min			
Max			
Cocaine			
N			
Mean			
SD			
Median			
Min			
Max			
Alcohol			
N			
Mean			
SD			
Median			
Min			
Max			
Cannabis			
N			
Mean			
SD			
Median			
Min			
Max			

Table 11: Summary of Patient Participant Baseline Characteristics by Evaluation Period

	**Baseline Evaluation Period (N=)**	**IF Evaluation Period (N=)**	**Total (N=)**
**UDS Results**			
Number of positive UDS results by substance	n/N (%)		
Benzodiazepines	n/N(%)		
Amphetamine			
Marijuana			
Methamphetamine			
Opiates (2000 ng)			
Opiates (300 ng)			
Cocaine			
Ecstasy			
Oxycodone			
Methadone			
Buprenorphine			
Fentanyl			
Number of days in the past 30 days that the patient participant			
Thought they overdosed on opioids			
N			
Mean			
SD			
Median			
Min			
Max			
Had an overdose involving opioids where they lost consciousness, needed medical care, or used more than they wanted to			
N			
Mean			
SD			
Median			
Min			
Max			

Table 11: Summary of Patient Participant Baseline Characteristics by Evaluation Period			
	**Baseline Evaluation Period (N=)**	**IF Evaluation Period (N=)**	**Total (N=)**
Go to the ED after an overdose involving opioids			
N			
Mean			
SD			
Median			
Min			
Max			
Number of inpatient admissions in past 30 days			
N			
Mean			
SD			
Min			
Median			
Max			
Number of outpatient visits in past 30 days			
N			
Mean			
SD			
Min			
Median			
Max			
Quality of Life (EQ-5D)			
Mobility			
I have no problems in walking about	N (%)		
I have some problems in walking about			
I am confined to bed			
Self-Care			
I have no problems with self-care			
I have some problems washing or dressing myself			
I am unable to wash or wash or dress myself			

Table 11: Summary of Patient Participant Baseline Characteristics by Evaluation Period

	Baseline Evaluation Period (N=)	IF Evaluation Period (N=)	Total (N=)
Usual Activities			
I have no problems with performing my usual activities			
I have some problems with performing my usual activities			
I am unable to perform my usual activities			
Pain/Discomfort			
I have no pain or discomfort			
I have moderate pain or discomfort			
I have extreme pain or discomfort			
Anxiety/Depression			
I am not anxious or depressed			
I am moderately anxious or depressed			
I am extremely anxious or depressed			
Overall score			
N			
Mean			
SD			
Min			
Median			
Max			

[1] Includes oxycodone, hydrocodone, fentanyl, morphine, hydromorphone, meperidine, oxymorphone, pentazocine, codeine.

Table 12: Summary of Patient Participant Baseline Characteristics by Site

	MA Johns Hopkins ED (N=)	GNY Mount Sinai ED (N=)	OV University of Cincinnati ED (N=)	PNW Harborview Medical Center ED (N=)	Total (N=)
**Demographics**					
Sex					
Male	N (XX.x %)				
Female	N (XX.x %)				
Age (Mean (SD))	N (X.X)				
Age					
< 18	N (XX.x %)				
18 - < 25	N (XX.x %)				
25 - < 35	N (XX.x %)				
35 - < 45	N (XX.x %)				
45 - < 55	N (XX.x %)				
55 - < 65	N (XX.x %)				
65 - < 75	N (XX.x %)				
75+	N (XX.x %)				
Ethnicity					
Not Hispanic or Latino	N (XX.x %)				
Hispanic or Latino	N (XX.x %)				
Don't know	N (XX.x %)				
Refused to answer	N (XX.x %)				
Race					
American Indian or Alaska Native	N (XX.x %)				
Asian	N (XX.x %)				
Black or African American	N (XX.x %)				
Native Hawaiian or Pacific Islander	N (XX.x %)				
White	N (XX.x %)				
Other	N (XX.x %)				
Multiracial	N (XX.x %)				
Don't know	N (XX.x %)				
Refused to answer	N (XX.x %)				

Table 12: Summary of Patient Participant Baseline Characteristics by Site

	MA Johns Hopkins ED (N=)	GNY Mount Sinai ED (N=)	OV University of Cincinnati ED (N=)	PNW Harborview Medical Center ED (N=)	Total (N=)
Education completed					
Less than high school diploma	N (XX.x %)				
High school graduate	N (XX.x %)				
GED or equivalent	N (XX.x %)				
Some college, no degree	N (XX.x %)				
Associate's degree: occupational, technical, or vocational program	N (XX.x %)				
Associate's degree: academic program	N (XX.x %)				
Bachelor's degree	N (XX.x %)				
Master's degree	N (XX.x %)				
Professional school degree	N (XX.x %)				
Doctoral degree	N (XX.x %)				
Marital status					
Married	N (XX.x %)				
Widowed	N (XX.x %)				
Divorced	N (XX.x %)				
Separated	N (XX.x %)				
Never married	N (XX.x %)				
Living with partner	N (XX.x %)				
Don't know	N (XX.x %)				
Employment					
Working now	N (XX.x %)				
Only temporarily laid off, sick leave, or maternity leave	N (XX.x %)				
Looking for work, unemployed	N (XX.x %)				
Retired	N (XX.x %)				
Disabled permanently or temporarily	N (XX.x %)				
Keeping house	N (XX.x %)				
Student	N (XX.x %)				
Other	N (XX.x %)				

Table 12: Summary of Patient Participant Baseline Characteristics by Site

	MA Johns Hopkins ED (N=)	GNY Mount Sinai ED (N=)	OV University of Cincinnati ED (N=)	PNW Harborview Medical Center ED (N=)	Total (N=)
Severity of opioid use disorder					
None					
Mild					
Moderate					
Severe					
Missing					
**Self-reported Substance Use**					
Any substance					
N					
Mean					
SD					
Median					
Min					
Max					
Opioids					
N					
Mean					
SD					
Median					
Min					
Max					
Opioid analgesics[1] (with prescription)					
N					
Mean					
SD					
Median					
Min					
Max					

Table 12: Summary of Patient Participant Baseline Characteristics by Site					
	MA Johns Hopkins ED (N=)	GNY Mount Sinai ED (N=)	OV University of Cincinnati ED (N=)	PNW Harborview Medical Center ED (N=)	Total (N=)
Opioid analgesics[1] (illicit)					
N					
Mean					
SD					
Median					
Min					
Max					
Heroin					
N					
Mean					
SD					
Median					
Min					
Max					
Fentanyl					
N					
Mean					
SD					
Median					
Min					
Max					
Buprenorphine					
N					
Mean					
SD					
Median					
Min					
Max					

Table 12: Summary of Patient Participant Baseline Characteristics by Site

	MA Johns Hopkins ED (N=)	GNY Mount Sinai ED (N=)	OV University of Cincinnati ED (N=)	PNW Harborview Medical Center ED (N=)	Total (N=)
Methadone					
N					
Mean					
SD					
Median					
Min					
Max					
Methamphetamine					
N					
Mean					
SD					
Median					
Min					
Max					
Cocaine					
N					
Mean					
SD					
Median					
Min					
Max					
Alcohol					
N					
Mean					
SD					
Median					
Min					
Max					

Table 12: Summary of Patient Participant Baseline Characteristics by Site

	MA Johns Hopkins ED (N=)	GNY Mount Sinai ED (N=)	OV University of Cincinnati ED (N=)	PNW Harborview Medical Center ED (N=)	Total (N=)
Cannabis					
N					
Mean					
SD					
Median					
Min					
Max					
**UDS Results**					
Number of positive UDS results by substance	n/N (%)				
Benzodiazepines	n/N(%)				
Amphetamine					
Marijuana					
Methamphetamine					
Opiates (2000 ng)					
Opiates (300 ng)					
Cocaine					
Ecstasy					
Oxycodone					
Methadone					
Buprenorphine					
Fentanyl					
Number of days in the past 30 days that the patient participant					
Thought they overdosed on opioids					
N					
Mean					
SD					
Median					
Min					
Max					

Table 12: Summary of Patient Participant Baseline Characteristics by Site

	MA Johns Hopkins ED (N=)	GNY Mount Sinai ED (N=)	OV University of Cincinnati ED (N=)	PNW Harborview Medical Center ED (N=)	Total (N=)
Had an overdose involving opioids where they lost consciousness, needed medical care, or used more than they wanted to					
N					
Mean					
SD					
Median					
Min					
Max					
Go to the ED after an overdose involving opioids					
N					
Mean					
SD					
Median					
Min					
Max					
Number of inpatient admissions in past 30 days					
N					
Mean					
SD					
Min					
Median					
Max					
Number of outpatient visits in past 30 days					
N					
Mean					
SD					
Min					
Median					
Max					

Table 12: Summary of Patient Participant Baseline Characteristics by Site					
	**MA Johns Hopkins ED (N=)**	**GNY Mount Sinai ED (N=)**	**OV University of Cincinnati ED (N=)**	**PNW Harborview Medical Center ED (N=)**	**Total (N=)**
Quality of Life (EQ-5D)					
Mobility					
I have no problems in walking about	N (%)				
I have some problems in walking about					
I am confined to bed					
Self-Care					
I have no problems with self-care					
I have some problems washing or dressing myself					
I am unable to wash or wash or dress myself					
Usual Activities					
I have no problems with performing my usual activities					
I have some problems with performing my usual activities					
I am unable to perform my usual activities					
Pain/Discomfort					
I have no pain or discomfort					
I have moderate pain or discomfort					
I have extreme pain or discomfort					
Anxiety/Depression					
I am not anxious or depressed					
I am moderately anxious or depressed					
I am extremely anxious or depressed					
Overall score					
N					
Mean					
SD					
Min					
Median					
Max					

CONFIDENTIAL

16.1.3 Primary Implementation Outcome

Table 13: Summary of Primary Implementation Outcome by Evaluation Period

Number	Baseline Evaluation Period (N=)	IF Evaluation Period (N=)	Total (N=)
Received ED-initiated BUP	N	N	N
Received referral for MAT	N	N	N
Received ED-initiated BUP with referral for ongoing MAT	N (XX.x%)	N (XX.x%)	N (XX.x%)

Table 14: Summary of Primary Implementation Outcome by Site and Evaluation Period								
	Baseline Evaluation Period				IF Evaluation Period			
**Number**	**MA Johns Hopkins ED (N=)**	**GNY Mount Sinai ED/Beth Israel (N=)**	**OV University of Cincinnati ED (N=)**	**PNW Harborview Med Center ED (N=)**	**MA Johns Hopkins ED (N=)**	**GNY Mount Sinai ED/Beth Israel (N=)**	**OV University of Cincinnati ED (N=)**	**PNW Harborview Med Center ED (N=)**
Received ED-initiated BUP	N				N			
Received Referral for MAT	N				N			
Received ED-initiated BUP with Referral for Ongoing MAT	N (XX.x%)				N (XX.x%)			

Table 15: Analysis Results for Primary Implementation Outcome				
GLMM		Bayesian Analysis		
**Intervention Coefficient**	**p-value**	**Risk Difference Estimation Time Point**	**Risk Difference**	**95% Credible Interval**
X.xx	x.xxx	Mid-point (day 632)	X.xx	X.xx; X.xx
		COVID-19 Pause (day 1423)	X.xx	X.xx; X.xx

Table 16: Summary of Primary Implementation Outcome by Sex and Evaluation Period				
	**Baseline Evaluation Period**		**IF Evaluation Period**	
**Number**	**Male (N=)**	**Female (N=)**	**Male (N=)**	**Female (N=)**
Received ED-initiated BUP	N		N	
Received Referral for MAT	N		N	
Received ED-initiated BUP with Referral for Ongoing MAT	N (XX.x%)		N (XX.x%)	

Table 17: Summary of Primary Implementation Outcome by Race and Evaluation Period						
	**Baseline Evaluation Period**			**IF Evaluation Period**		
**Number**	**White (N=)**	**Black/African American (N=)**	**Other (N=)**	**White (N=)**	**Black/African American(N=)**	**Other (N=)**
Received ED-initiated BUP	N			N		
Received Referral for MAT	N			N		
Received ED-initiated BUP with Referral for Ongoing MAT	N (XX.x%)			N (XX.x%)		

Table 18: Summary of Primary Implementation Outcome by Ethnicity and Evaluation Period

	Baseline Evaluation Period		IF Evaluation Period	
**Number**	**Hispanic or Latino (N=)**	**Not Hispanic or Latino (N=)**	**Hispanic or Latino (N=)**	**Not Hispanic or Latino (N=)**
Received ED-initiated BUP	N		N	
Received Referral for MAT	N		N	
Received ED-initiated BUP with Referral for Ongoing MAT	N (XX.x%)		N (XX.x%)	

Table 19: Final Covariate Adjusted Model Analysis Results for Primary Implementation Outcome			
**Covariate[1]**	**N**	**Coefficient**	**95% Credible Interval**
Study Phase			
BEP	N	[ref]	[ref]
IFEP	N	X.x	X.xx; X.xx
Sex			
Male	N	[ref]	[ref]
Female	N		
Race			
White	N	[ref]	[ref]
Black/African American	N		
Other	N		
Ethnicity			
Not Hispanic/Latino	N	[ref]	[ref]
Hispanic/Latino	N		
Sex-by-study phase interaction[2]			
Male	N	X.x	X.xx; X.xx
Female	N	X.x	X.xx; X.xx
Race-by-study phase interaction[2]			
White	N	X.x	X.xx; X.xx
Black	N	X.x	X.xx; X.xx
Other	N	X.x	X.xx; X.xx
Ethnicity-by-study phase interaction[2]			
Not Hispanic/Latino	N	X.x	X.xx; X.xx
Hispanic/Latino	N	X.x	X.xx; X.xx

[1] Note that only covariates which remained in the final covariate-adjusted model will be included in this table.

[2] Coefficient and corresponding credible intervals are presented for the intervention effect in a particular subgroup (e.g., males).

Table 20: Summary of MOUD Practices by Site Over Time from EHR-abstracted Data										
	**Baseline Evaluation Period**					**IF Evaluation Period**				
	**MA Johns Hopkins ED**	**GNY Mount Sinai ED**	**OV University of Cincinnati ED**	**PNW Harborview Medical Center ED**	**Total**	**MA Johns Hopkins ED**	**GNY Mount Sinai ED**	**OV University of Cincinnati ED**	**PNW Harborview Medical Center ED**	**Total**
Buprenorphine										
Administered	N									
Prescribed	N									
Administered and prescribed	N									
Unique providers administering or prescribing	N									
Naloxone										
Dispensed	N									
Prescribed	N									
Dispensed and prescribed	N									
Unique providers dispensing or prescribing	N									
X-waiver										
Physicians	N									
APPs	N									
Additional staff (counselors/peers)	N									

Table 21: COVID-19 Sensitivity Analyses Results for Primary Implementation Outcome

Sensitivity Analysis	GLMM		Bayesian Analysis		
	Intervention Coefficient	p-value	Risk Difference Estimation Time Point	Risk Difference	95% Credible Interval
Pre-COVID data	X.xx	x.xxx	Mid-point (day 632)	X.xx	X.xx; X.xx
			COVID-19 Pause (day 1423)	X.xx	X.xx; X.xx
COVID pause removed	X.xx	x.xxx	Mid-point (day 632)	X.xx	X.xx; X.xx
			COVID-19 Pause (day 1423)	X.xx	X.xx; X.xx

16.1.4 Primary Effectiveness Outcome

Table 22: Summary of Primary Effectiveness Outcome by Evaluation Period

Number	Baseline Evaluation Period (N=)	IF Evaluation Period (N=)
Self-report Treatment Engagement	N	N
Facility confirmed Treatment Engagement	N	N
Missing primary effectiveness outcome (i.e., to be imputed as non-engaged)	N	N
Engagement in formal addiction treatment for OUD at Day 30	N (XX.x%)	N (XX.x%)

Table 23: Summary of Primary Effectiveness Outcome by Site and Evaluation Period

	Baseline Evaluation Period				IF Evaluation Period			
**Number**	**MA Johns Hopkins ED (N=)**	**GNY Mount Sinai ED/Beth Israel (N=)**	**OV University of Cincinnati ED (N=)**	**PNW Harborview Med Center ED (N=)**	**MA Johns Hopkins ED (N=)**	**GNY Mount Sinai ED/Beth Israel (N=)**	**OV University of Cincinnati ED (N=)**	**PNW Harborview Med Center ED (N=)**
Self-report Treatment Engagement	N				N			
Facility confirmed Treatment Engagement	N				N			
Missing primary effectiveness outcome (i.e., to be imputed as non-engaged)	N				N			
Engagement in formal addiction treatment for OUD at Day 30	N (XX.x%)				N (XX.x%)			

Table 24: Analysis Results for Primary Effectiveness Outcome				
**GLMM**		**Bayesian Analysis**		
**Intervention Effect Coefficient**	**p-value**	**Risk Difference Estimation Time Point**	**Risk Difference**	**95% Credible Interval**
X.xx	x.xxx	Mid-point (time = 632)	X.xx	X.xx; X.xx
		COVID-19 Pause (time = 1423)	X.xx	X.xx; X.xx

Table 25: Summary of Primary Effectiveness Outcome by Sex and Evaluation Period				
	Baseline Evaluation Period		IF Evaluation Period	
**Number**	**Male (N=)**	**Female (N=)**	**Male (N=)**	**Female (N=)**
Self-report Treatment Engagement	N		N	
Facility confirmed Treatment Engagement	N		N	
Missing primary effectiveness outcome (i.e., to be imputed as non-engaged)	N		N	
Engagement in formal addiction treatment for OUD at Day 30	N (XX.x%)		N (XX.x%)	

Table 26: Summary of Primary Effectiveness Outcome by Race and Evaluation Period						
	Baseline Evaluation Period			IF Evaluation Period		
**Number**	**White (N=)**	**Black/African American (N=)**	**Other (N=)**	**White (N=)**	**Black/African American (N=)**	**Other (N=)**
Self-report Treatment Engagement	N			N		
Facility confirmed Treatment Engagement	N			N		
Missing primary effectiveness outcome (i.e., to be imputed as non-engaged)	N			N		
Engagement in formal addiction treatment for OUD at Day 30	N (XX.x%)			N (XX.x%)		

Table 27: Summary of Primary Effectiveness Outcome by Ethnicity and Evaluation Period

	Baseline Evaluation Period		IF Evaluation Period	
**Number**	**Hispanic or Latino (N=)**	**Not Hispanic or Latino (N=)**	**Hispanic or Latino (N=)**	**Not Hispanic or Latino (N=)**
Self-report Treatment Engagement	N	N		
Facility confirmed Treatment Engagement	N	N		
Missing primary effectiveness outcome (i.e., to be imputed as non-engaged)	N	N		
Engagement in formal addiction treatment for OUD at Day 30	N (XX.x%)	N (XX.x%)		

Table 28: Final Covariate Adjusted Model Analysis Results for Primary Effectiveness Outcome

Covariate[1]	N	Coefficient	95% Credible Interval
Study Phase			
BEP	N	[ref]	[ref]
IFEP	N	X.x	X.xx; X.xx
Sex			
Male	N	[ref]	[ref]
Female	N	X.x	X.xx; X.xx
Race			
White	N	[ref]	[ref]
Black	N	X.x	X.xx; X.xx
Other	N	X.x	X.xx; X.xx
Ethnicity			
Not Hispanic/Latino		[ref]	[ref]
Hispanic/Latino		X.x	X.xx; X.xx
Sex-by-study phase interaction[2]			
Male	N	X.x	X.xx; X.xx
Female	N	X.x	X.xx; X.xx
Race-by-study phase interaction[2]			
White	N	X.x	X.xx; X.xx
Black	N	X.x	X.xx; X.xx
Other	N	X.x	X.xx; X.xx
Ethnicity-by-study phase interaction[2]			
Not Hispanic/Latino	N	X.x	X.xx; X.xx
Hispanic/Latino	N	X.x	X.xx; X.xx

[1] Note that only covariates which remained in the final covariate-adjusted model will be included in this table.

[2] Coefficient and corresponding credible intervals are presented for the intervention effect in a particular subgroup (e.g., males).

Table 29: COVID-19 Sensitivity Analyses Results for Primary Effectiveness Outcome

Sensitivity Analysis	GLMM		Bayesian Analysis		
	Intervention Coefficient	p-value	Risk Difference Estimation Time Point	Risk Difference	95% Credible Interval
Pre-COVID data	X.xx	x.xxx	Mid-point (day 632)	X.xx	X.xx; X.xx
			COVID-19 Pause (day 1423)	X.xx	X.xx; X.xx
COVID pause removed	X.xx	x.xxx	Mid-point (day 632)	X.xx	X.xx; X.xx
			COVID-19 Pause (day 1423)	X.xx	X.xx; X.xx

Table 30: Summary of Treatment Engagement at Day 30 during the IF Evaluation Period by ED-initiated BUP Status: All Patient-participants (Missing Engagement Status Imputed as Not Engaged)

ED-initiated BUP Status	Pre-IF Cohort (N =)	Post-IF Cohort (N =)
No	N (XX.x%)	N (XX.x%)
Yes	N (XX.x%)	N (XX.x%)

Table 31: Summary of Treatment Engagement at Day 30 during the IF Evaluation Period by ED-initiated BUP Status: Patient-participants with Non-missing Engagement Status (No Imputation)

ED-initiated BUP Status	Pre-IF Cohort (N =)	Post-IF Cohort (N =)
No	N (XX.x%)	N (XX.x%)
Yes	N (XX.x%)	N (XX.x%)

Table 32: Analysis of Treatment Engagement at Day 30 during the IF Evaluation Period by ED-initiated BUP Status

Method of Handling Missing Data	N	p-value
Missing engagement status imputed as not engaged)	N	X.xxx
No Imputation (i.e., complete case)	N	X.xxx

16.1.5 Safety

Table 33: Summary of Hospitalizations Post Index Visit by Evaluation Period

	Baseline Evaluation Period (N=)	IF Evaluation Period (N=)	Total (N=)
Number of hospitalizations			
Number of patient participants with at least one hospitalization			
Number of hospitalizations per patient participant			
0			
1			
2			
3			
4			
5 or more			

Listing 1A: Listing of Hospitalizations Post Index Visit by Evaluation Period

Baseline Evaluation Period

Site	Patient Participant ID	Date of Index ED Visit	Hospitalization Date	Discharge Date	Chief Complaint	Discharge Diagnosis
MA Johns Hopkins ED						
GNY Mount Sinai ED						
PNW Harborview Medical Center ED						
OV University of Cincinnati ED						

Listing 1B: Listing of Hospitalizations Post Index Visit by Evaluation Period

IF Evaluation Period

Site	Patient Participant ID	Date of Index ED Visit	Hospitalization Date	Discharge Date	Chief Complaint	Discharge Diagnosis
MA Johns Hopkins ED						
GNY Mount Sinai ED						
PNW Harborview Medical Center ED						
OV University of Cincinnati ED						

Listing 2: Listing of Hospitalizations Post Index Visit for Ineligible Participants						
**Site**	**Patient Participant ID**	**Date of Index ED Visit**	**Hospitalization Date**	**Discharge Date**	**Chief Complaint**	**Discharge Diagnosis**
MA Johns Hopkins ED						
GNY Mount Sinai ED						
PNW Harborview Medical Center ED						
OV University of Cincinnati ED						

Table 34: Summary of ED Visits Post Index Visit by Evaluation Period

	Baseline Evaluation Period (N=)	IF Evaluation Period (N=)	Total (N=)
Number of ED visits			
Number of patient participants with at least one ED visit			
Number of ED visits per patient participant			
0			
1			
2			
3			
4			
5 or more			

Listing 3A: Listing of ED Visits Post Index Visit by Evaluation Period

Baseline Evaluation Period

Site	Patient Participant ID	Date of Index ED Visit	ED Visit Date	Discharge Date	Chief Complaint	Discharge Diagnosis
MA Johns Hopkins ED						
GNY Mount Sinai ED						
PNW Harborview Medical Center ED						
OV University of Cincinnati ED						

Listing 3B: Listing of ED Visits Post Index Visit by Evaluation Period

IF Evaluation Period

Site	Patient Participant ID	Date of Index ED Visit	ED Visit Date	Discharge Date	Chief Complaint	Discharge Diagnosis
MA Johns Hopkins ED						
GNY Mount Sinai ED						
PNW Harborview Medical Center ED						
OV University of Cincinnati ED						

Listing 4: Listing of ED Visits Post Index Visit for Ineligible Participants						
**Site**	**Patient Participant ID**	**Date of Index ED Visit**	**ED Visit Date**	**Discharge Date**	**Chief Complaint**	**Discharge Diagnosis**
MA Johns Hopkins ED						
GNY Mount Sinai ED						
PNW Harborview Medical Center ED						
OV University of Cincinnati ED						

Table 35: Summary of Overdoses by Evaluation Period

Events in the past 30 days	Baseline Evaluation Period (N=)	IF Evaluation Period (N=)	Total (N=)
Number of days overdosed on opioids			
N			
Mean			
Min			
Median			
Max			
Number of days overdose(s) requiring medical assistance			
N			
Mean			
Min			
Median			
Max			

Listing 5A: Listing of Overdoses by Evaluation Period

Baseline Evaluation Period

Site	Patient Participant ID	Number of Days in the Past 30 Days that the Patient Participant Overdosed	
		On Opioids	And Needed Medical Assistance
MA Johns Hopkins ED			
GNY Mount Sinai ED			
PNW Harborview Medical Center ED			
OV University of Cincinnati ED			

Listing 5B: Listing of Overdoses by Evaluation Period

IF Evaluation Period

Site	Patient Participant ID	Number of Days in the Past 30 Days that the Patient Participant Overdosed	
		On Opioids	And Needed Medical Assistance
MA Johns Hopkins ED			
GNY Mount Sinai ED			
PNW Harborview Medical Center ED			
OV University of Cincinnati ED			

Listing 6: Listing of Overdoses for Enrolled Patient Participants that Were Ineligible

Site	Patient Participant ID	Number of Days in the Past 30 Days that the Patient Participant Overdosed	
		On Opioids	And Needed Medical Assistance
MA Johns Hopkins ED			
GNY Mount Sinai ED			
PNW Harborview Medical Center ED			
OV University of Cincinnati ED			

Table 36: Summary of Suicide Risk by Evaluation Period

Visit	PHQ-9 Question 9	Baseline Evaluation Period (N=)	IF Evaluation Period (N=)	Total (N=)
Baseline Visit	Over the last 2 weeks, how often have you been bothered by thoughts that you would be better off dead, or of hurting yourself in some way?			
	Missing			
	Not at all			
	Several Days			
	More than Half the Days			
	Nearly Every Day			
Follow-up Visit	Over the last 2 weeks, how often have you been bothered by thoughts that you would be better off dead, or of hurting yourself in some way?			
	Missing			
	Not at all			
	Several Days			
	More than Half the Days			
	Nearly Every Day			

Listing 7A: Listing of Suicide Risk by Evaluation Period

Baseline Evaluation Period

Site	Patient Participant ID	Date of Enrollment	Visit	Date of Entry	Over the last 2 weeks, how often have you been bothered by thoughts that you would be better off dead, or of hurting yourself in some way?
MA Johns Hopkins ED					
GNY Mount Sinai ED					
PNW Harborview Medical Center ED					
OV University of Cincinnati ED					

All visits are included for participants who endorsed having thoughts of being better off dead or of hurting themselves in some way on 'Several Days', 'More than Half the Days' or 'Nearly Every Day'.

Responses of 'Several Days' are highlighted in yellow, 'More than Half the Days' are highlighted in orange and 'Nearly Every Day' are highlighted in red.

Listing 7B: Listing of Suicide Risk by Evaluation Period

IF Evaluation Period

Site	Participant ID	Date of Enrollment	Visit	Date of Entry	Over the last 2 weeks, how often have you been bothered by thoughts that you would be better off dead, or of hurting yourself in some way?
MA Johns Hopkins ED					
GNY Mount Sinai ED					
PNW Harborview Medical Center ED					
OV University of Cincinnati ED					

All visits are included for participants who endorsed having thoughts of being better off dead or of hurting themselves in some way on 'Several Days', 'More than Half the Days' or 'Nearly Every Day'.

Responses of 'Several Days' are highlighted in yellow, 'More than Half the Days' are highlighted in orange and 'Nearly Every Day' are highlighted in red.

Listing 8: Listing of Suicide Risk for Enrolled Patient Participants who Were Ineligible

Site	Patient Participant ID	Date of Enrollment	Visit	Date of Entry	Over the last 2 weeks, how often have you been bothered by thoughts that you would be better off dead, or of hurting yourself in some way?
MA Johns Hopkins ED					
GNY Mount Sinai ED					
PNW Harborview Medical Center ED					
OV University of Cincinnati ED					

All visits are included for participants who endorsed having thoughts of being better off dead or of hurting themselves in some way on 'Several Days', 'More than Half the Days' or 'Nearly Every Day'.

Responses of 'Several Days' are highlighted in yellow, 'More than Half the Days' are highlighted in orange and 'Nearly Every Day' are highlighted in red.

Listing 9: Listing of Deaths by Evaluation Period[1]

							MedDRA v23.1	
**Evaluation Period**	**Site**	**Patient Participant ID**	**Description of Death**	**Date of Enrollment**	**Date of Death**	**Related to OD?**	**Preferred Term**	**System Organ Class**
Baseline Evaluation Period								
IF Evaluation Period								

[1] Includes only deaths during 30-Day follow-up window, not those identified in NDI search.

16.1.6 Data Quality

Table 37: Summary of Data Audits by Site

Site	Date of Audit	Total Fields Audited[1]	Total Data Discrepancies[2]	Error Rate (%)
MA Johns Hopkins ED	MM/DD/YYY	N	N	X.X %
	Subtotal	N	N	X.X %
GNY Mount Sinai ED				
	Subtotal			
OV University of Cincinnati ED				
	Subtotal			
PNW Harborview Medical Center ED				
	Subtotal			
Total				

[1] Fields reviewed at monitoring visit comparing the database to source documentation.

[2] Fields discrepant between database and source documentation.

Table 38: Summary of Protocol Deviations by Site					
	**MA Johns Hopkins ED**	**GNY Mount Sinai ED**	**PNW Harborview Medical Center ED**	**OV University of Cincinnati ED**	**Total**
Total number of protocol deviations	N	N	N	N	N
Number of patient participants impacted per protocol deviation					
None	N (XX.X %)				
One	N (XX.X %)				
More than one	N (XX.X %)				
Total number of major protocol deviations	N				
Type of major protocol deviation					
Ineligible participant enrolled/inclusion/exclusion criteria not met	N (XX.X %)				
Breach of Confidentiality	N (XX.X %)				
Total number of minor protocol deviations	N				
Type of minor protocol deviation					
Informed consent/assent process not properly conducted and/or documented	N (XX.X %)				
Biologic specimen not collected/processed as per protocol	N (XX.X %)				
Non IRB approved/outdated/obsolete informed consent/assent documents used	N (XX.X %)				
Study assessments not completed/followed as per protocol	N (XX.X %)				
Other significant deviation issues	N (XX.X %)				

Listing 10: Listing of Protocol Deviations by Site

Site	Related Patient Participant IDs	Date of Protocol Deviation	Date Protocol Deviation Entered in EDC	Deviation Type	Deviation Type (other)	Related to COVID-19?	Deviation Description	Corrective Action Taken	IRB Reporting Required?	IRB Notified at Continuing Review?	Expected/ Actual IRB Report Date
MA Johns Hopkins ED								.			
GNY Mount Sinai ED								.			
OV University of Cincinnati ED								.			
PNW Harborview Medical Center ED											

Table 39: Summary of Provider Participant Characteristics by Site

Characteristic	MA Johns Hopkins ED (N=)	GNY Mount Sinai ED (N=)	OV University of Cincinnati ED (N=)	PNW Harborview Medical Center ED (N=)	Total (N=)
Sex					
Male	N (XX.x%)				
Female	N (XX.x%)				
Age (Mean (SD))	N (X.x)				
Age					
< 18	N (XX.x%)				
18 - < 25	N (XX.x%)				
25 - < 35	N (XX.x%)				
35 - < 45	N (XX.x%)				
45 - < 55	N (XX.x%)				
55 - < 65	N (XX.x%)				
65 - < 75	N (XX.x%)				
75+	N (XX.x%)				
Ethnicity					
Not Hispanic or Latino	N (XX.x%)				
Hispanic or Latino	N (XX.x%)				
Don't know	N (XX.x%)				
Refused to answer	N (XX.x%)				
Race					
American Indian or Alaska Native	N (XX.x%)				
Asian	N (XX.x%)				
Black or African American	N (XX.x%)				
Native Hawaiian or Pacific Islander	N (XX.x%)				
White	N (XX.x%)				
Other	N (XX.x%)				
Multiracial	N (XX.x%)				
Don't know	N (XX.x%)				
Refused to answer	N (XX.x%)				

CONFIDENTIAL

Table 39: Summary of Provider Participant Characteristics by Site

Characteristic	MA Johns Hopkins ED (N=)	GNY Mount Sinai ED (N=)	OV University of Cincinnati ED (N=)	PNW Harborview Medical Center ED (N=)	Total (N=)
Education completed					
Less than high school diploma	N (XX.x%)				
High school graduate	N (XX.x%)				
GED or equivalent	N (XX.x%)				
Some college, no degree	N (XX.x%)				
Associate's degree: occupational, technical, or vocational program	N (XX.x%)				
Associate's degree: academic program	N (XX.x%)				
Bachelor's degree	N (XX.x%)				
Master's degree	N (XX.x%)				
Professional school degree	N (XX.x%)				
Doctoral degree	N (XX.x%)				
Marital status					
Married	N (XX.x%)				
Widowed	N (XX.x%)				
Divorced	N (XX.x%)				
Separated	N (XX.x%)				
Never married	N (XX.x%)				
Living with partner	N (XX.x%)				
Don't know	N (XX.x%)				
Employment					
Working now	N (XX.x%)				
Only temporarily laid off, sick leave, or maternity leave	N (XX.x%)				
Looking for work, unemployed	N (XX.x%)				
Retired	N (XX.x%)				
Disabled permanently or temporarily	N (XX.x%)				
Keeping house	N (XX.x%)				
Student	N (XX.x%)				
Other	N (XX.x%)				

CONFIDENTIAL

16.1.7 Focus Group Participant Characteristics

Table 40A: Summary of Focus Groups Participant Characteristics: Wave 1

	**Characteristic**	**MA Johns Hopkins ED (N=)**	**GNY Mount Sinai ED (N=)**	**OV University of Cincinnati ED (N=)**	**PNW Harborview Medical Center ED (N=)**	**Total (N=)**
Patients only	Sex					
	Male	N (XX.x %)				
	Female	N (XX.x %)				
	Age (Mean (SD))	N (X.X)				
	Age					
	< 18	N (XX.x %)				
	18 - < 25	N (XX.x %)				
	25 - < 35	N (XX.x %)				
	35 - < 45	N (XX.x %)				
	45 - < 55	N (XX.x %)				
	55 - < 65	N (XX.x %)				
	65 - < 75	N (XX.x %)				
	75+	N (XX.x %)				
	Ethnicity					
	Not Hispanic or Latino	N (XX.x %)				
	Hispanic or Latino	N (XX.x %)				
	Don't know	N (XX.x %)				
	Refused to answer	N (XX.x %)				
	Race					
	American Indian or Alaska Native	N (XX.x %)				
	Asian	N (XX.x %)				
	Black or African American	N (XX.x %)				
	Native Hawaiian or Pacific Islander	N (XX.x %)				
	White	N (XX.x %)				
	Other	N (XX.x %)				
	Multiracial	N (XX.x %)				
	Don't know	N (XX.x %)				
	Refused to answer	N (XX.x %)				

Table 40A: Summary of Focus Groups Participant Characteristics: Wave 1						
	**Characteristic**	**MA Johns Hopkins ED (N=)**	**GNY Mount Sinai ED (N=)**	**OV University of Cincinnati ED (N=)**	**PNW Harborview Medical Center ED (N=)**	**Total (N=)**
	Education completed					
	Less than high school diploma	N (XX.x %)				
	High school graduate	N (XX.x %)				
	GED or equivalent	N (XX.x %)				
	Some college, no degree	N (XX.x %)				
	Associate's degree: occupational, technical, or vocational program	N (XX.x %)				
	Associate's degree: academic program	N (XX.x %)				
	Bachelor's degree	N (XX.x %)				
	Master's degree	N (XX.x %)				
	Professional school degree	N (XX.x %)				
	Doctoral degree	N (XX.x %)				
	Marital status					
	Married	N (XX.x %)				
	Widowed	N (XX.x %)				
	Divorced	N (XX.x %)				
	Separated	N (XX.x %)				
	Never married	N (XX.x %)				
	Living with partner	N (XX.x %)				
	Don't know	N (XX.x %)				
	Employment					
	Working now	N (XX.x %)				
	Only temporarily laid off, sick leave, or maternity leave	N (XX.x %)				
	Looking for work, unemployed	N (XX.x %)				
	Retired	N (XX.x %)				
	Disabled permanently or temporarily	N (XX.x %)				
	Keeping house	N (XX.x %)				
	Student	N (XX.x %)				
	Other	N (XX.x %)				

Table 40B: Summary of Focus Groups Participant Characteristics: Wave 1

	Characteristic	MA Johns Hopkins ED (N=)	GNY Mount Sinai ED (N=)	OV University of Cincinnati ED (N=)	PNW Harborview Medical Center ED (N=)	Total (N=)
Non-patients only	Sex					
	Male	N (XX.x %)				
	Female	N (XX.x %)				
	Age (Mean (SD))	N (X.X)				
	Age					
	< 18	N (XX.x %)				
	18 - < 25	N (XX.x %)				
	25 - < 35	N (XX.x %)				
	35 - < 45	N (XX.x %)				
	45 - < 55	N (XX.x %)				
	55 - < 65	N (XX.x %)				
	65 - < 75	N (XX.x %)				
	75+	N (XX.x %)				
	Ethnicity					
	Not Hispanic or Latino	N (XX.x %)				
	Hispanic or Latino	N (XX.x %)				
	Don't know	N (XX.x %)				
	Refused to answer	N (XX.x %)				
	Race					
	American Indian or Alaska Native	N (XX.x %)				
	Asian	N (XX.x %)				
	Black or African American	N (XX.x %)				
	Native Hawaiian or Pacific Islander	N (XX.x %)				
	White	N (XX.x %)				
	Other	N (XX.x %)				
	Multiracial	N (XX.x %)				
	Don't know	N (XX.x %)				
	Refused to answer	N (XX.x %)				

Table 40B: Summary of Focus Groups Participant Characteristics: Wave 1						
	**Characteristic**	**MA Johns Hopkins ED (N=)**	**GNY Mount Sinai ED (N=)**	**OV University of Cincinnati ED (N=)**	**PNW Harborview Medical Center ED (N=)**	**Total (N=)**
	Education completed					
	Less than high school diploma	N (XX.x %)				
	High school graduate	N (XX.x %)				
	GED or equivalent	N (XX.x %)				
	Some college, no degree	N (XX.x %)				
	Associate's degree: occupational, technical, or vocational program	N (XX.x %)				
	Associate's degree: academic program	N (XX.x %)				
	Bachelor's degree	N (XX.x %)				
	Master's degree	N (XX.x %)				
	Professional school degree	N (XX.x %)				
	Doctoral degree	N (XX.x %)				
	Marital status					
	Married	N (XX.x %)				
	Widowed	N (XX.x %)				
	Divorced	N (XX.x %)				
	Separated	N (XX.x %)				
	Never married	N (XX.x %)				
	Living with partner	N (XX.x %)				
	Don't know	N (XX.x %)				
	Employment					
	Working now	N (XX.x %)				
	Only temporarily laid off, sick leave, or maternity leave	N (XX.x %)				
	Looking for work, unemployed	N (XX.x %)				
	Retired	N (XX.x %)				
	Disabled permanently or temporarily	N (XX.x %)				
	Keeping house	N (XX.x %)				
	Student	N (XX.x %)				
	Other	N (XX.x %)				

Table 40C: Summary of Focus Groups Participant Characteristics: Wave 1

	Characteristic	MA Johns Hopkins ED (N=)	GNY Mount Sinai ED (N=)	OV University of Cincinnati ED (N=)	PNW Harborview Medical Center ED (N=)	Total (N=)
All participants (Patients and non-patients)	Sex					
	Male	N (XX.x %)				
	Female	N (XX.x %)				
	Age (Mean (SD))	N (X.X)				
	Age					
	< 18	N (XX.x %)				
	18 - < 25	N (XX.x %)				
	25 - < 35	N (XX.x %)				
	35 - < 45	N (XX.x %)				
	45 - < 55	N (XX.x %)				
	55 - < 65	N (XX.x %)				
	65 - < 75	N (XX.x %)				
	75+	N (XX.x %)				
	Ethnicity					
	Not Hispanic or Latino	N (XX.x %)				
	Hispanic or Latino	N (XX.x %)				
	Don't know	N (XX.x %)				
	Refused to answer	N (XX.x %)				
	Race					
	American Indian or Alaska Native	N (XX.x %)				
	Asian	N (XX.x %)				
	Black or African American	N (XX.x %)				
	Native Hawaiian or Pacific Islander	N (XX.x %)				
	White	N (XX.x %)				
	Other	N (XX.x %)				
	Multiracial	N (XX.x %)				
	Don't know	N (XX.x %)				
	Refused to answer	N (XX.x %)				

Table 40C: Summary of Focus Groups Participant Characteristics: Wave 1

	Characteristic	MA Johns Hopkins ED (N=)	GNY Mount Sinai ED (N=)	OV University of Cincinnati ED (N=)	PNW Harborview Medical Center ED (N=)	Total (N=)
	Education completed					
	Less than high school diploma	N (XX.x %)				
	High school graduate	N (XX.x %)				
	GED or equivalent	N (XX.x %)				
	Some college, no degree	N (XX.x %)				
	Associate's degree: occupational, technical, or vocational program	N (XX.x %)				
	Associate's degree: academic program	N (XX.x %)				
	Bachelor's degree	N (XX.x %)				
	Master's degree	N (XX.x %)				
	Professional school degree	N (XX.x %)				
	Doctoral degree	N (XX.x %)				
	Marital status					
	Married	N (XX.x %)				
	Widowed	N (XX.x %)				
	Divorced	N (XX.x %)				
	Separated	N (XX.x %)				
	Never married	N (XX.x %)				
	Living with partner	N (XX.x %)				
	Don't know	N (XX.x %)				
	Employment					
	Working now	N (XX.x %)				
	Only temporarily laid off, sick leave, or maternity leave	N (XX.x %)				
	Looking for work, unemployed	N (XX.x %)				
	Retired	N (XX.x %)				
	Disabled permanently or temporarily	N (XX.x %)				
	Keeping house	N (XX.x %)				
	Student	N (XX.x %)				
	Other	N (XX.x %)				

Table 41A: Summary of Focus Groups Participant Characteristics: Wave 2

	Characteristic	MA Johns Hopkins ED (N=)	GNY Mount Sinai ED (N=)	OV University of Cincinnati ED (N=)	PNW Harborview Medical Center ED (N=)	Total (N=)
Patients only	Sex					
	Male	N (XX.x %)				
	Female	N (XX.x %)				
	Age (Mean (SD))	N (X.X)				
	Age					
	< 18	N (XX.x %)				
	18 - < 25	N (XX.x %)				
	25 - < 35	N (XX.x %)				
	35 - < 45	N (XX.x %)				
	45 - < 55	N (XX.x %)				
	55 - < 65	N (XX.x %)				
	65 - < 75	N (XX.x %)				
	75+	N (XX.x %)				
	Ethnicity					
	Not Hispanic or Latino	N (XX.x %)				
	Hispanic or Latino	N (XX.x %)				
	Don't know	N (XX.x %)				
	Refused to answer	N (XX.x %)				
	Race					
	American Indian or Alaska Native	N (XX.x %)				
	Asian	N (XX.x %)				
	Black or African American	N (XX.x %)				
	Native Hawaiian or Pacific Islander	N (XX.x %)				
	White	N (XX.x %)				
	Other	N (XX.x %)				
	Multiracial	N (XX.x %)				
	Don't know	N (XX.x %)				
	Refused to answer	N (XX.x %)				

Table 41A: Summary of Focus Groups Participant Characteristics: Wave 2						
	Characteristic	MA Johns Hopkins ED (N=)	GNY Mount Sinai ED (N=)	OV University of Cincinnati ED (N=)	PNW Harborview Medical Center ED (N=)	Total (N=)
	Education completed					
	Less than high school diploma	N (XX.x %)				
	High school graduate	N (XX.x %)				
	GED or equivalent	N (XX.x %)				
	Some college, no degree	N (XX.x %)				
	Associate's degree: occupational, technical, or vocational program	N (XX.x %)				
	Associate's degree: academic program	N (XX.x %)				
	Bachelor's degree	N (XX.x %)				
	Master's degree	N (XX.x %)				
	Professional school degree	N (XX.x %)				
	Doctoral degree	N (XX.x %)				
	Marital status					
	Married	N (XX.x %)				
	Widowed	N (XX.x %)				
	Divorced	N (XX.x %)				
	Separated	N (XX.x %)				
	Never married	N (XX.x %)				
	Living with partner	N (XX.x %)				
	Don't know	N (XX.x %)				
	Employment					
	Working now	N (XX.x %)				
	Only temporarily laid off, sick leave, or maternity leave	N (XX.x %)				
	Looking for work, unemployed	N (XX.x %)				
	Retired	N (XX.x %)				
	Disabled permanently or temporarily	N (XX.x %)				
	Keeping house	N (XX.x %)				
	Student	N (XX.x %)				
	Other	N (XX.x %)				

Table 41B: Summary of Focus Groups Participant Characteristics: Wave 2						
	**Characteristic**	**MA Johns Hopkins ED (N=)**	**GNY Mount Sinai ED (N=)**	**OV University of Cincinnati ED (N=)**	**PNW Harborview Medical Center ED (N=)**	**Total (N=)**
Non-patients only	Sex					
	Male	N (XX.x %)				
	Female	N (XX.x %)				
	Age (Mean (SD))	N (X.X)				
	Age					
	< 18	N (XX.x %)				
	18 - < 25	N (XX.x %)				
	25 - < 35	N (XX.x %)				
	35 - < 45	N (XX.x %)				
	45 - < 55	N (XX.x %)				
	55 - < 65	N (XX.x %)				
	65 - < 75	N (XX.x %)				
	75+	N (XX.x %)				
	Ethnicity					
	Not Hispanic or Latino	N (XX.x %)				
	Hispanic or Latino	N (XX.x %)				
	Don't know	N (XX.x %)				
	Refused to answer	N (XX.x %)				
	Race					
	American Indian or Alaska Native	N (XX.x %)				
	Asian	N (XX.x %)				
	Black or African American	N (XX.x %)				
	Native Hawaiian or Pacific Islander	N (XX.x %)				
	White	N (XX.x %)				
	Other	N (XX.x %)				
	Multiracial	N (XX.x %)				
	Don't know	N (XX.x %)				
	Refused to answer	N (XX.x %)				

Table 41B: Summary of Focus Groups Participant Characteristics: Wave 2

	Characteristic	MA Johns Hopkins ED (N=)	GNY Mount Sinai ED (N=)	OV University of Cincinnati ED (N=)	PNW Harborview Medical Center ED (N=)	Total (N=)
	Education completed					
	Less than high school diploma	N (XX.x %)				
	High school graduate	N (XX.x %)				
	GED or equivalent	N (XX.x %)				
	Some college, no degree	N (XX.x %)				
	Associate's degree: occupational, technical, or vocational program	N (XX.x %)				
	Associate's degree: academic program	N (XX.x %)				
	Bachelor's degree	N (XX.x %)				
	Master's degree	N (XX.x %)				
	Professional school degree	N (XX.x %)				
	Doctoral degree	N (XX.x %)				
	Marital status					
	Married	N (XX.x %)				
	Widowed	N (XX.x %)				
	Divorced	N (XX.x %)				
	Separated	N (XX.x %)				
	Never married	N (XX.x %)				
	Living with partner	N (XX.x %)				
	Don't know	N (XX.x %)				
	Employment					
	Working now	N (XX.x %)				
	Only temporarily laid off, sick leave, or maternity leave	N (XX.x %)				
	Looking for work, unemployed	N (XX.x %)				
	Retired	N (XX.x %)				
	Disabled permanently or temporarily	N (XX.x %)				
	Keeping house	N (XX.x %)				
	Student	N (XX.x %)				
	Other	N (XX.x %)				

Table 41C: Summary of Focus Groups Participant Characteristics: Wave 2							
	**Characteristic**	**MA Johns Hopkins ED (N=)**	**GNY Mount Sinai ED (N=)**	**OV University of Cincinnati ED (N=)**	**PNW Harborview Medical Center ED (N=)**	**Total (N=)**
All participants (Patients and non-patients)	Sex					
	Male	N (XX.x %)				
	Female	N (XX.x %)				
	Age (Mean (SD))	N (X.X)				
	Age					
	< 18	N (XX.x %)				
	18 - < 25	N (XX.x %)				
	25 - < 35	N (XX.x %)				
	35 - < 45	N (XX.x %)				
	45 - < 55	N (XX.x %)				
	55 - < 65	N (XX.x %)				
	65 - < 75	N (XX.x %)				
	75+	N (XX.x %)				
	Ethnicity					
	Not Hispanic or Latino	N (XX.x %)				
	Hispanic or Latino	N (XX.x %)				
	Don't know	N (XX.x %)				
	Refused to answer	N (XX.x %)				
	Race					
	American Indian or Alaska Native	N (XX.x %)				
	Asian	N (XX.x %)				
	Black or African American	N (XX.x %)				
	Native Hawaiian or Pacific Islander	N (XX.x %)				
	White	N (XX.x %)				
	Other	N (XX.x %)				
	Multiracial	N (XX.x %)				
	Don't know	N (XX.x %)				
	Refused to answer	N (XX.x %)				

Table 41C: Summary of Focus Groups Participant Characteristics: Wave 2

	Characteristic	MA Johns Hopkins ED (N=)	GNY Mount Sinai ED (N=)	OV University of Cincinnati ED (N=)	PNW Harborview Medical Center ED (N=)	Total (N=)
	Education completed					
	Less than high school diploma	N (XX.x %)				
	High school graduate	N (XX.x %)				
	GED or equivalent	N (XX.x %)				
	Some college, no degree	N (XX.x %)				
	Associate's degree: occupational, technical, or vocational program	N (XX.x %)				
	Associate's degree: academic program	N (XX.x %)				
	Bachelor's degree	N (XX.x %)				
	Master's degree	N (XX.x %)				
	Professional school degree	N (XX.x %)				
	Doctoral degree	N (XX.x %)				
	Marital status					
	Married	N (XX.x %)				
	Widowed	N (XX.x %)				
	Divorced	N (XX.x %)				
	Separated	N (XX.x %)				
	Never married	N (XX.x %)				
	Living with partner	N (XX.x %)				
	Don't know	N (XX.x %)				
	Employment					
	Working now	N (XX.x %)				
	Only temporarily laid off, sick leave, or maternity leave	N (XX.x %)				
	Looking for work, unemployed	N (XX.x %)				
	Retired	N (XX.x %)				
	Disabled permanently or temporarily	N (XX.x %)				
	Keeping house	N (XX.x %)				
	Student	N (XX.x %)				
	Other	N (XX.x %)				

Table 42A: Summary of Focus Groups Participant Characteristics: Wave 3

	Characteristic	MA Johns Hopkins ED (N=)	GNY Mount Sinai ED (N=)	OV University of Cincinnati ED (N=)	PNW Harborview Medical Center ED (N=)	Total (N=)
Patients only	Sex					
	Male	N (XX.x %)				
	Female	N (XX.x %)				
	Age (Mean (SD))	N (X.X)				
	Age					
	< 18	N (XX.x %)				
	18 - < 25	N (XX.x %)				
	25 - < 35	N (XX.x %)				
	35 - < 45	N (XX.x %)				
	45 - < 55	N (XX.x %)				
	55 - < 65	N (XX.x %)				
	65 - < 75	N (XX.x %)				
	75+	N (XX.x %)				
	Ethnicity					
	Not Hispanic or Latino	N (XX.x %)				
	Hispanic or Latino	N (XX.x %)				
	Don't know	N (XX.x %)				
	Refused to answer	N (XX.x %)				
	Race					
	American Indian or Alaska Native	N (XX.x %)				
	Asian	N (XX.x %)				
	Black or African American	N (XX.x %)				
	Native Hawaiian or Pacific Islander	N (XX.x %)				
	White	N (XX.x %)				
	Other	N (XX.x %)				
	Multiracial	N (XX.x %)				
	Don't know	N (XX.x %)				
	Refused to answer	N (XX.x %)				

Table 42A: Summary of Focus Groups Participant Characteristics: Wave 3

	Characteristic	MA Johns Hopkins ED (N=)	GNY Mount Sinai ED (N=)	OV University of Cincinnati ED (N=)	PNW Harborview Medical Center ED (N=)	Total (N=)
	Education completed					
	Less than high school diploma	N (XX.x %)				
	High school graduate	N (XX.x %)				
	GED or equivalent	N (XX.x %)				
	Some college, no degree	N (XX.x %)				
	Associate's degree: occupational, technical, or vocational program	N (XX.x %)				
	Associate's degree: academic program	N (XX.x %)				
	Bachelor's degree	N (XX.x %)				
	Master's degree	N (XX.x %)				
	Professional school degree	N (XX.x %)				
	Doctoral degree	N (XX.x %)				
	Marital status					
	Married	N (XX.x %)				
	Widowed	N (XX.x %)				
	Divorced	N (XX.x %)				
	Separated	N (XX.x %)				
	Never married	N (XX.x %)				
	Living with partner	N (XX.x %)				
	Don't know	N (XX.x %)				
	Employment					
	Working now	N (XX.x %)				
	Only temporarily laid off, sick leave, or maternity leave	N (XX.x %)				
	Looking for work, unemployed	N (XX.x %)				
	Retired	N (XX.x %)				
	Disabled permanently or temporarily	N (XX.x %)				
	Keeping house	N (XX.x %)				
	Student	N (XX.x %)				
	Other	N (XX.x %)				

Table 42B: Summary of Focus Groups Participant Characteristics: Wave 3

	Characteristic	MA Johns Hopkins ED (N=)	GNY Mount Sinai ED (N=)	OV University of Cincinnati ED (N=)	PNW Harborview Medical Center ED (N=)	Total (N=)
Non-patients only	Sex					
	Male	N (XX.x %)				
	Female	N (XX.x %)				
	Age (Mean (SD))	N (X.X)				
	Age					
	< 18	N (XX.x %)				
	18 - < 25	N (XX.x %)				
	25 - < 35	N (XX.x %)				
	35 - < 45	N (XX.x %)				
	45 - < 55	N (XX.x %)				
	55 - < 65	N (XX.x %)				
	65 - < 75	N (XX.x %)				
	75+	N (XX.x %)				
	Ethnicity					
	Not Hispanic or Latino	N (XX.x %)				
	Hispanic or Latino	N (XX.x %)				
	Don't know	N (XX.x %)				
	Refused to answer	N (XX.x %)				
	Race					
	American Indian or Alaska Native	N (XX.x %)				
	Asian	N (XX.x %)				
	Black or African American	N (XX.x %)				
	Native Hawaiian or Pacific Islander	N (XX.x %)				
	White	N (XX.x %)				
	Other	N (XX.x %)				
	Multiracial	N (XX.x %)				
	Don't know	N (XX.x %)				
	Refused to answer	N (XX.x %)				

Table 42B: Summary of Focus Groups Participant Characteristics: Wave 3						
	Characteristic	MA Johns Hopkins ED (N=)	GNY Mount Sinai ED (N=)	OV University of Cincinnati ED (N=)	PNW Harborview Medical Center ED (N=)	Total (N=)
	Education completed					
	Less than high school diploma	N (XX.x %)				
	High school graduate	N (XX.x %)				
	GED or equivalent	N (XX.x %)				
	Some college, no degree	N (XX.x %)				
	Associate's degree: occupational, technical, or vocational program	N (XX.x %)				
	Associate's degree: academic program	N (XX.x %)				
	Bachelor's degree	N (XX.x %)				
	Master's degree	N (XX.x %)				
	Professional school degree	N (XX.x %)				
	Doctoral degree	N (XX.x %)				
	Marital status					
	Married	N (XX.x %)				
	Widowed	N (XX.x %)				
	Divorced	N (XX.x %)				
	Separated	N (XX.x %)				
	Never married	N (XX.x %)				
	Living with partner	N (XX.x %)				
	Don't know	N (XX.x %)				
	Employment					
	Working now	N (XX.x %)				
	Only temporarily laid off, sick leave, or maternity leave	N (XX.x %)				
	Looking for work, unemployed	N (XX.x %)				
	Retired	N (XX.x %)				
	Disabled permanently or temporarily	N (XX.x %)				
	Keeping house	N (XX.x %)				
	Student	N (XX.x %)				
	Other	N (XX.x %)				

Table 42C: Summary of Focus Groups Participant Characteristics: Wave 3

	Characteristic	MA Johns Hopkins ED (N=)	GNY Mount Sinai ED (N=)	OV University of Cincinnati ED (N=)	PNW Harborview Medical Center ED (N=)	Total (N=)
All participants (Patients and non-patients)	Sex					
	Male	N (XX.x %)				
	Female	N (XX.x %)				
	Age (Mean (SD))	N (X.X)				
	Age					
	< 18	N (XX.x %)				
	18 - < 25	N (XX.x %)				
	25 - < 35	N (XX.x %)				
	35 - < 45	N (XX.x %)				
	45 - < 55	N (XX.x %)				
	55 - < 65	N (XX.x %)				
	65 - < 75	N (XX.x %)				
	75+	N (XX.x %)				
	Ethnicity					
	Not Hispanic or Latino	N (XX.x %)				
	Hispanic or Latino	N (XX.x %)				
	Don't know	N (XX.x %)				
	Refused to answer	N (XX.x %)				
	Race					
	American Indian or Alaska Native	N (XX.x %)				
	Asian	N (XX.x %)				
	Black or African American	N (XX.x %)				
	Native Hawaiian or Pacific Islander	N (XX.x %)				
	White	N (XX.x %)				
	Other	N (XX.x %)				
	Multiracial	N (XX.x %)				
	Don't know	N (XX.x %)				
	Refused to answer	N (XX.x %)				

Table 42C: Summary of Focus Groups Participant Characteristics: Wave 3

	Characteristic	MA Johns Hopkins ED (N=)	GNY Mount Sinai ED (N=)	OV University of Cincinnati ED (N=)	PNW Harborview Medical Center ED (N=)	Total (N=)
	Education completed					
	Less than high school diploma	N (XX.x %)				
	High school graduate	N (XX.x %)				
	GED or equivalent	N (XX.x %)				
	Some college, no degree	N (XX.x %)				
	Associate's degree: occupational, technical, or vocational program	N (XX.x %)				
	Associate's degree: academic program	N (XX.x %)				
	Bachelor's degree	N (XX.x %)				
	Master's degree	N (XX.x %)				
	Professional school degree	N (XX.x %)				
	Doctoral degree	N (XX.x %)				
	Marital status					
	Married	N (XX.x %)				
	Widowed	N (XX.x %)				
	Divorced	N (XX.x %)				
	Separated	N (XX.x %)				
	Never married	N (XX.x %)				
	Living with partner	N (XX.x %)				
	Don't know	N (XX.x %)				
	Employment					
	Working now	N (XX.x %)				
	Only temporarily laid off, sick leave, or maternity leave	N (XX.x %)				
	Looking for work, unemployed	N (XX.x %)				
	Retired	N (XX.x %)				
	Disabled permanently or temporarily	N (XX.x %)				
	Keeping house	N (XX.x %)				
	Student	N (XX.x %)				
	Other	N (XX.x %)				

17.0 REFERENCES

1. Beebe J. Rapid assessment process. In: Kempf-Leonard K, ed. *The encyclopedia of social measurement.* 2005:285-291.
2. Fleming MF, Barry KL. A three-sample test of a masked alcohol screening questionnaire. Alcohol and alcoholism (Oxford, Oxfordshire). 1991;26(1):81-91.
3. Fleming MF, Bruno M, Barry K, Fost N. Informed consent, deception, and the use of disguised alcohol questionnaires. The American journal of drug and alcohol abuse. 1989;15(3):309-319.Hussey MA, Hughes JP. Design and analysis of stepped wedge cluster randomized trials. Contemporary clinical trials. 2007;28(2):182-191.
4. Sobel L, Sobell M. Timeline follow-back: a technique for assessing self-reported alcohol consumption. In: Litten R, Allen J, eds. *Measuring Alcohol Consumption: Psychosocial and Biological Methods*. Totowa, NJ: Humana Press; 1992:41-72.
5. Hawk KF, D'Onofrio G, Chawarski MC. *Barriers and Facilitators to Clinician Readiness to Provide Emergency Department–Initiated Buprenorphine*. JAMA Netw Open. 2020;3(5):e204561. doi:10.1001/jamanetworkopen.2020.4561.
6. Woertman W, de Hoop E, Moerbeek M, Zuidema SU, Gerritsen DL, Teerenstra S. *Stepped wedge designs could reduce the required sample size in cluster randomized trials*. Journal of clinical epidemiology*.* 2013;66(7):752-758.
7. French MT, Popovici I, Tapsell L. *The economic costs of substance abuse treatment: updated estimates and cost bands for program assessment and reimbursement*. J Subst Abuse Treat. 2008 Dec;35(4):462-9.

18.0 APPENDIX: BAYESIAN ANALYSIS

To construct a Frequentist confidence interval for a parameter, one follows a recipe that generates from the data a region that, with specified probability, encloses the parameter. One tests a level 0.05 whether a parameter is or is not equal 9 by determining whether the 95% confidence interval for the parameter does or does not enclose 0.

The Bayesian statistics, the parameter is associated with a probability distribution this distribution describes one's belief that the parameter could equal various numeric values. Credibility intervals, the Bayesian analogous to Frequentist confidence limits, are regions of the distribution that integrate to specific probabilities. A Bayesian analogue to testing whether a parameter is or is not equal 0 involves discovering whether 95% credibility interval for the parameter does or does not enclose 0. So a Bayesian analogue to power it the proportion of simulated data sets in which the 0.95-level credibility interval for the treatment effect does not enclose zero. (Equivalently for 1-tailed tests, the parameter differs from 0 if the integral of its distribution from 0 to infinity exceed 0.95).

Figures B5 and B8 show the Bayesian power curves in the alternative and bull cases we have been considering. These figures, based on simulation of 1000 iterations per scenario (the Bayesian method takes much longer – 8 hours for 250 iterations per scenario – than the Frequentist approach – 4 hours for 10,000 iterations per scenario), are comparable to Figures 1 and 3, respectively. They indicate that the Bayesian approach may be useful secondary analysis method for the primary hypotheses under discussion. Note, however, that although autocorrelation was a problem in only a fraction of a percent of the alternative-case iterations, about 27% of the null-case iterations generated SAS warnings that there was still significant autocorrelation after 500 lags.

The Bayesian approach undertaken here uses diffuse prior. Perhaps using historical data to estimate priors could lead to an increase in power, but this is investigated here.

Figures B6-B7 shows Bayes estimates for $p_1, p_2,$ and $p_1 - p2$. The average credibility intervals for p_1 and p_2 are somewhat wider than corresponding confidence intervals. The reason for this is unclear. Although the treatment effect is an odds ratio. i.e., $p_2(1-p_1)/[p_1(1-p_2)]$ in both approaches, an attractive aspect of the Bayes MCMC method is that it is easy to get the posterior distribution of the simple treatment difference, as shown $p_2 - p_1$ in Figure B7. The credibility interval for seems less $p_2 - p_1$ sensitive to ICC than other interval estimates investigated.

18.1 Figure B5: Bayes power; Alternative Cases

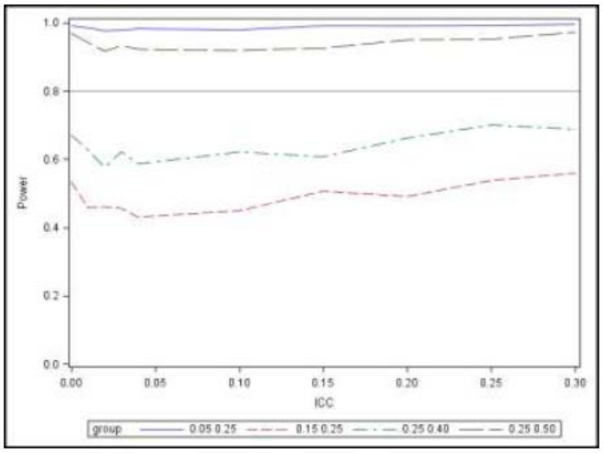

18.2 Figure B6: Bayes Credibility Intervals for P1, P2; Alternative Cases

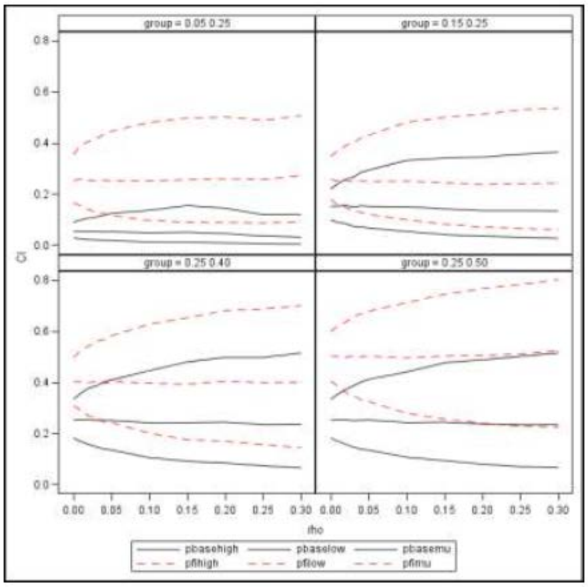

18.3 Figure B7: Bayes Credibility Intervals for P1-P2; Alternative Cases

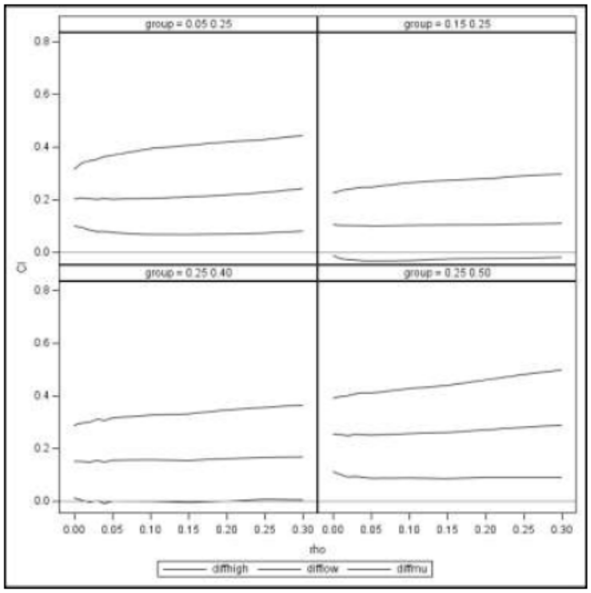

Figures B8-B10 give test size and credibility intervals under the “Null” scenarios of Table 4. Figures B8-B9 are comparable to their Frequentist analogues (Figure 4.-5, sections 11.8.3-11.8.4) As in figure B7, the credibility interval for p2 – p1 (Figure B10) seems less sensitive to ICC than other interval estimates investigated.

18.4 Figure B8: Bayes Test Size; Null Cases

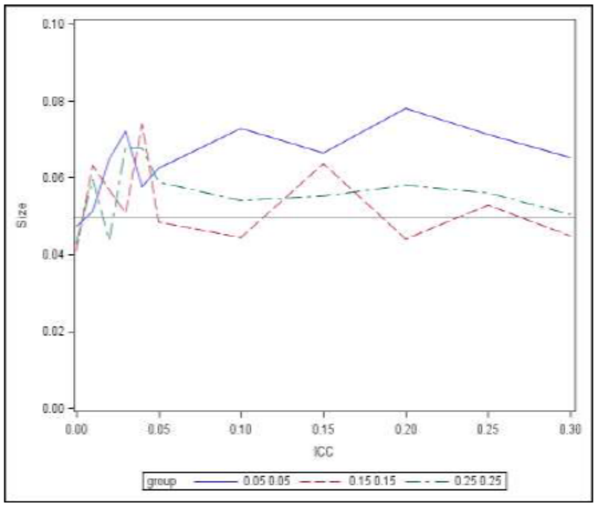

18.5 Figure B9: Bayes Credibility Intervals for P1, P2; Null Cases

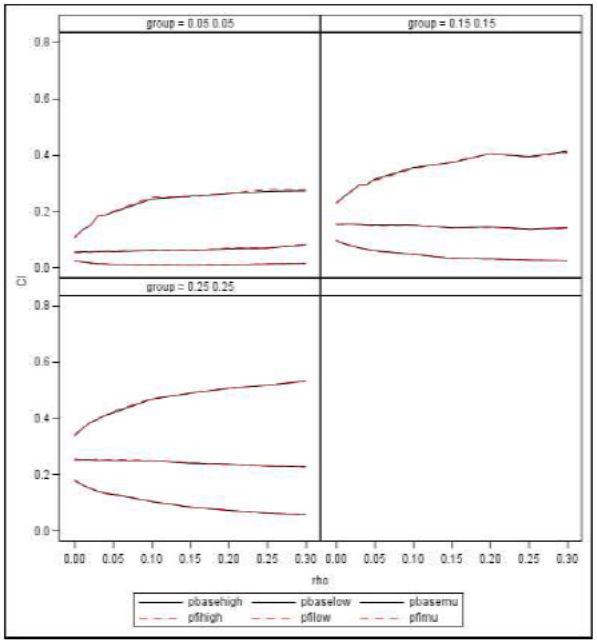

18.6 Figure B10: Bayes Credibility Intervals P1-P2; Null Cases

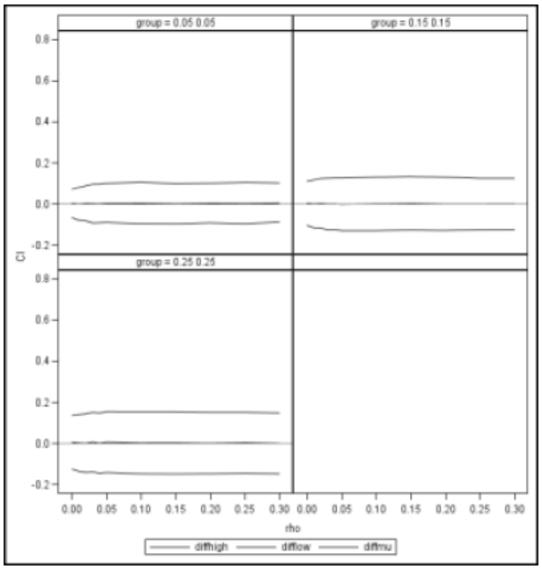

We document here the SAS code for the Bayesian approach, which employs the Markov Chain Monte Carlo method.

```
Proc mcmc data = simmsimul nbi = 1000 nmc = 10000 thin = 2 see = 159
    monitor – (beta0-beta2 beta2_gt_0 p1 p2 pdiff) statistics = (summary intervals);
    parms beta0-beta2 0;
    parms sigma2 1;
    prior beta0-beta2~normal(mean = 0, var = 1000):
    prior sigma2~ igamma(shape – 0.001, scale = 0.001)
    random b0 ~normal(mean = 0, var = sigma2) subject = site;
    array p[2]
    p[treat+1] = logistic(beta0 + beta1 * 22 + beta2 * treat);
    pdiff = p2 – p1
    eta = beta0 + beta1 * month + beta2 * treat + b0;
    pi = logistic(eta)
    model z ~binomial(n = trials, p = pi);
    beta2_gt_0 = beta2 > 0;
    run;
```

REVISED SAP
Revised on 04/25/22

Analytic Model Adjustments

This section provides a description of adjustments to the computational model used in the planned analyses that were necessary to ensure the validity of the performed tests of statistical significance of the hypothesized effects. In general, two updates were implemented: (1) exclusion of time in the model; and (2) the method of adjusting for site.

Time. The proposed analytical plan in the SAP document included a fixed effect for the evaluation period (Baseline vs IF evaluation, called “arm” in the SAP), fixed linear effect of time (days from the study start to the date of enrollment of each patient in the observational cohorts study, a continuous variable called “time”) and a random effect for site (called” site”). Such a model is appropriate for analyzing data collected in a fully implemented multisite stepped-wedge study design. An important characteristic of a stepped-wedge study design is a substantial overlap of control and treatment periods. **(Figure 1)**

FIGURE 1. Characteristic of a Stepped-Wedge Study Design: Substantial Overlap of Control and Treatment Periods

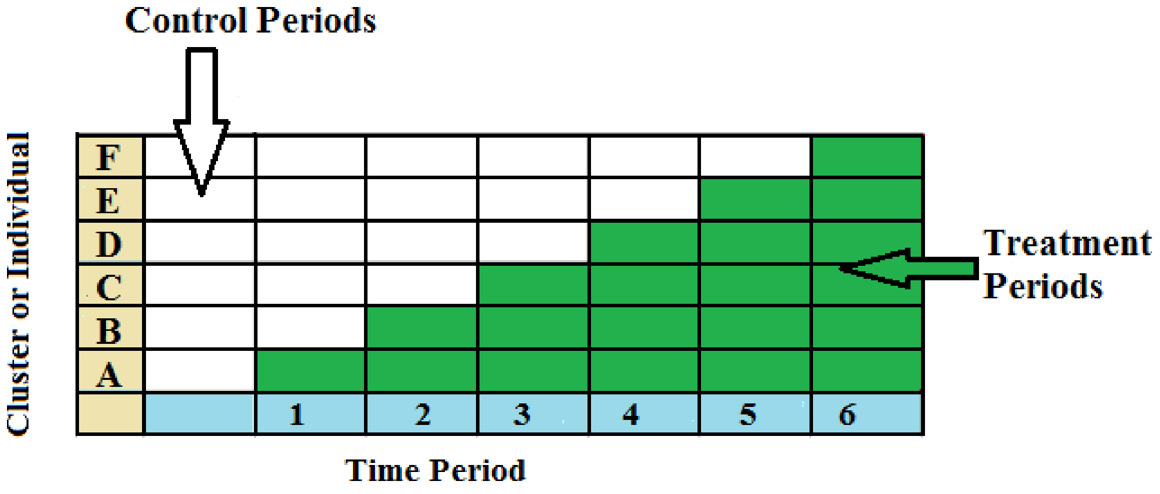

In such a design, the effect of time can be analytically partially disentangled from the treatment effect. However, the CTN0069 study was not implemented as a modified stepped-wedge design as initially described. As implemented, in CTN-0069, there was virtually no overlap between the baseline evaluation and IF evaluation periods. Evaluation of the distributional properties of the time variable data indicated that this variable, originally included as one of the explanatory factors in the analytical model, overlaps 96% with the intervention/treatment factor in the study. Including two highly overlapping variables in the analytical model can create a computational, statistical, and mathematical error. Collinearity, or high association between two explanatory variables

means that the collinear variables contain the same information about the dependent variable, and that these only nominally different measures actually quantify the same phenomenon or information. In CTN-0069, the variables “time” and “arm” are highly redundant. The best and most statistically robust analytical models are called “low noise" models in which the predictor variables each correlate highly with the outcome variable but correlate with each other at most only minimally. Collinearity and redundancy may lead to a failure to reject a false null hypothesis of no intervention effect. Consequently, the “time” variable was removed from the adjusted computational model.

Site. Additionally, the four geographically diverse study sites were purposefully selected to represent different clinical contexts and different patient populations. Conceptually, these EDs cannot be considered as a random sample of all EDs in the US, and probabilistically, no four values selected from a large population can be considered to represent randomness, even if they are drawn by chance. Consequently, site was included in the primary analytic models as a fixed effect, and not a random one in order to provide additional information on between site differences. The study results demonstrated not only statistical difference effects between the sites, but also substantial, large, important, and informative site differences.

Thus, the final analytic model for both the primary implementation and effectiveness outcomes was a logistic regression model with fixed effects for site and evaluation period.

<u>Site Differences for Primary Effectiveness Outcome</u>

As noted in the manuscript, there were substantial site differences with respect to the primary effectiveness outcome. Three of the four sites was associated with an increase in treatment engagement during the IF evaluation period, however for one site there was a decrease. To explore this further, a logistic regression model was fit assessing the site-by-evaluation period interaction. In this model, there was a statistically significant interaction (p=0.021) indicating that the differences observed across sites was statistically significant. Future work could explore these relationships further, including evaluation of differences in site characteristics that may explain the observed qualitative interaction.
