# Supplementary material for: Implementation Facilitation to Promote Emergency Department–Initiated Buprenorphine for Opioid Use Disorder
Source: JAMA Netw Open. 2023 Apr 5;6(4):e235439. doi: 10.1001/jamanetworkopen.2023.5439 (PMC10077107; doi:10.1001/jamanetworkopen.2023.5439)
Supplement: Supplement 3. — eFigure 1. Study Timeline eFigure 2. Time Series Analysis Plot Across All Sites eFigure 3. Time Series Analysis Plots for Each Study Site [file jamanetwopen-e235439-s003.pdf]

Supplemental Online Content

D'Onofrio G, Edelman EJ, Hawk KF, et al. Implementation facilitation to promote emergency department–initiated buprenorphine for opioid use disorder. *JAMA Netw Open*. 2023;6(4):e235439. doi:10.1001/jamanetworkopen.2023.5439

eFigure 1. Study Timeline

eFigure 2. Time Series Analysis Plot Across All Sites

eFigure 3. Time Series Analysis Plots for Each Study Site

This supplemental material has been provided by the authors to give readers additional information about their work.

© 2023 D'Onofrio G et al. *JAMA Network Open.*

eFigure 1. Study Timeline

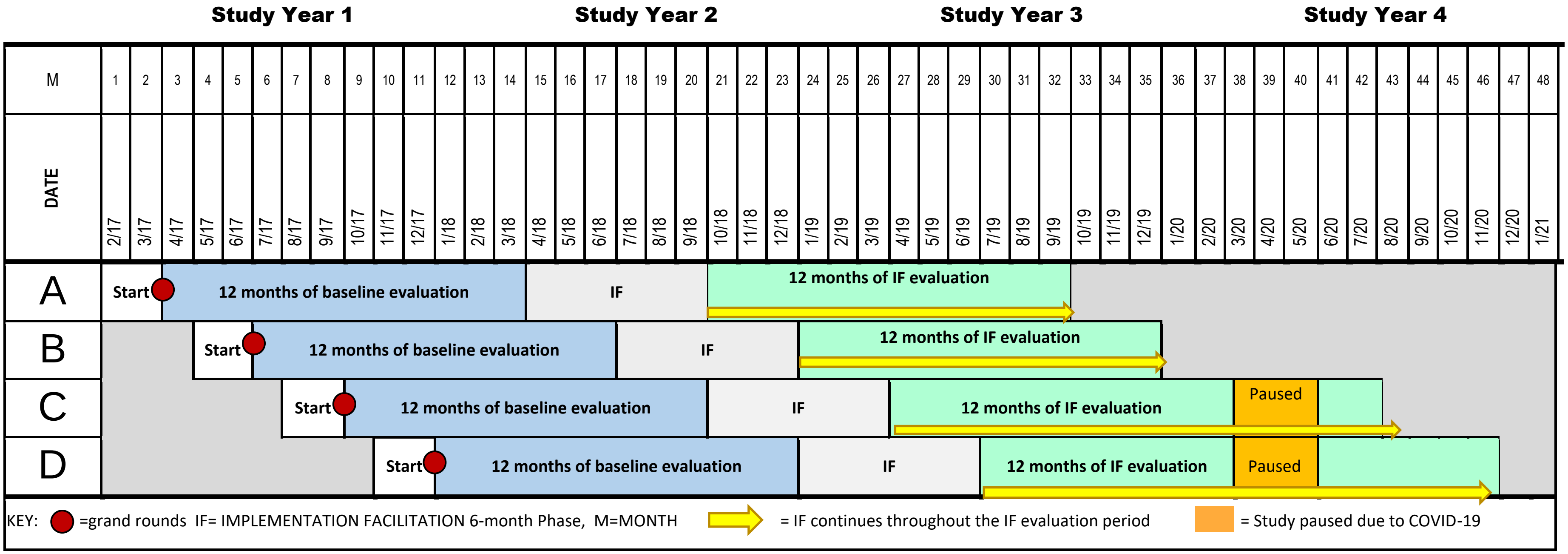

© 2023 D'Onofrio G et al. *JAMA Network Open.*

eFigure 2. Time Series Analysis Plot Across All Sites

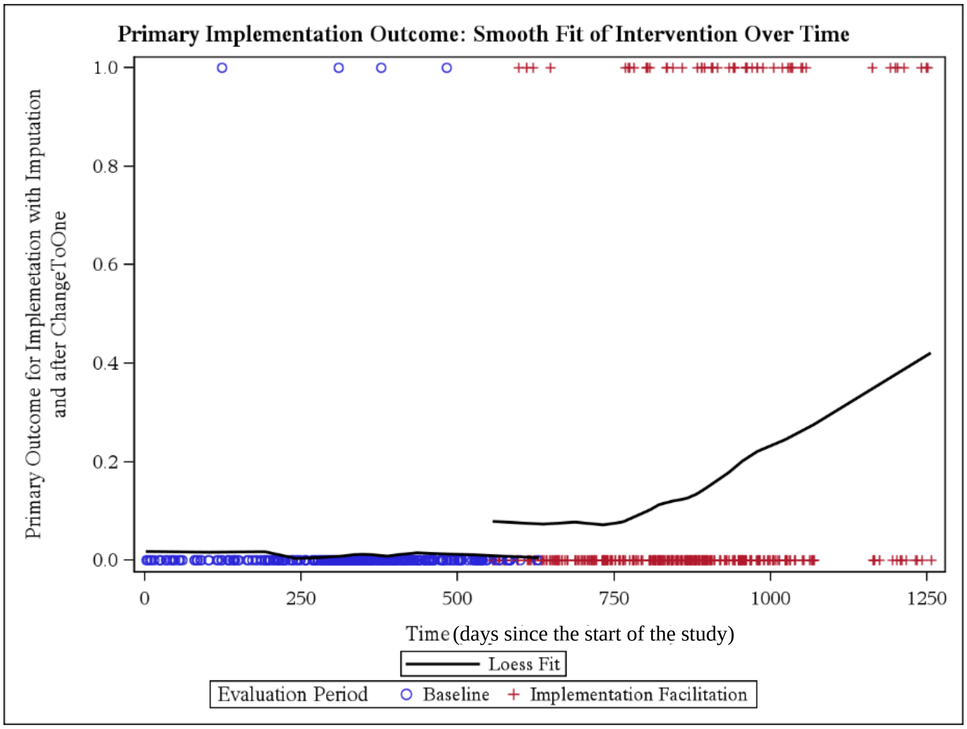

© 2023 D'Onofrio G et al. *JAMA Network Open.*

eFigure 3. Time Series Analysis Plots for Each Study Site

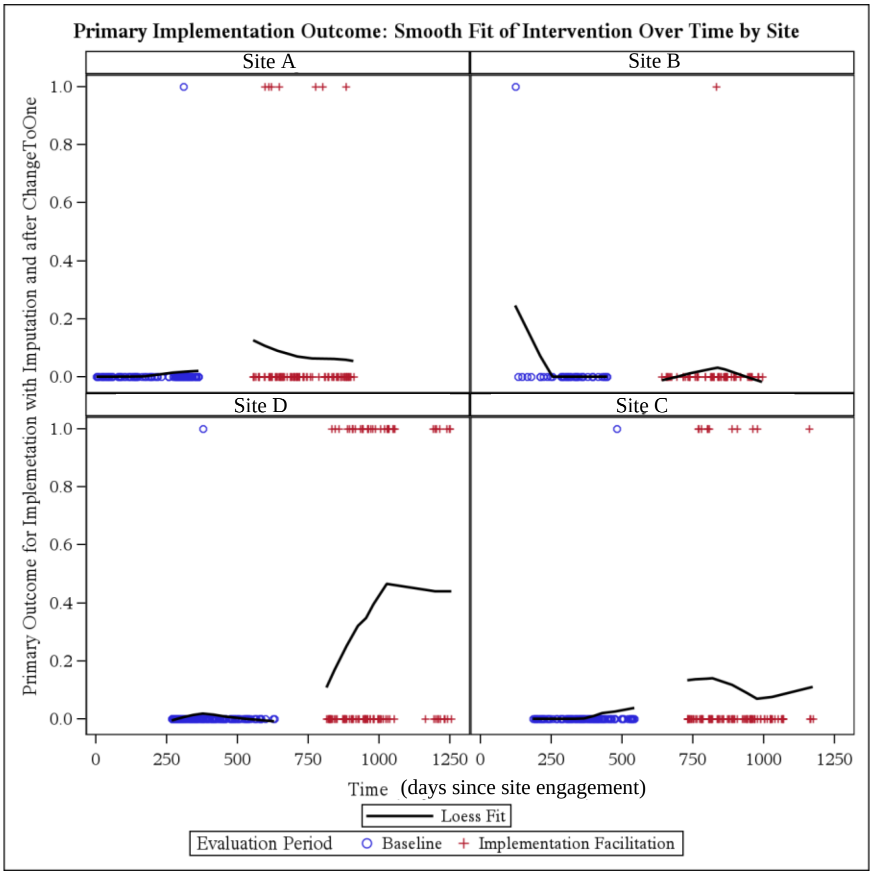

© 2023 D'Onofrio G et al. *JAMA Network Open.*
