# Supplementary material for: Implementation Facilitation to Promote Emergency Department–Initiated Buprenorphine for Opioid Use Disorder
Source: JAMA Netw Open. 2023 Apr 5;6(4):e235439. doi: 10.1001/jamanetworkopen.2023.5439 (PMC10077107; doi:10.1001/jamanetworkopen.2023.5439)
Supplement: Supplement 4. — Data Sharing Statement [file jamanetwopen-e235439-s004.pdf]

Data Sharing Statement

D'Onofrio. Implementation Facilitation to Promote Emergency Department-Initiated Buprenorphine for Opioid Use Disorder. *JAMA Netw Open*. Published April 05, 2023. doi:10.1001/jamanetworkopen.2023.5439

Data
Data available: Yes
Data types: Deidentified participant data, Data dictionary
How to access data: This study will comply with the NIH Data Sharing Policy and Implementation Guidance (https://grants.nih.gov/grants/policy/data_sharing/data_sharing_guidance.htm). Investigators will also register and report results of the trial in ClinicalTrials.gov, consistent with the requirements of the Policy on the Dissemination of NIH-Funded Clinical Trial Information and the Clinical Trials Registration (https://grants.nih.gov/policy/clinical-trials/reporting/understanding/nih-policy.htm). Primary data for this study will be available to the public in the NIDA data repository, per NIDA CTN policy. For more details on data sharing please visit https://datashare.nida.nih.gov/. No qualitative date will be shared.
When available: With publication

Supporting Documents
Document types: None

Additional Information
Who can access the data: Researchers whose proposed use of the data has been approved.
Types of analyses: Researchers with specified aims e.g. meta-analyses or new research questions with analytical approaches clearly defined.
Mechanisms of data availability: After approval of a proposal, and with a signed data access agreement. The original investigator team will not supply financial support.
